# Supplementary figures and images for: Referral to Slimming World in UK Stop Smoking Services (SWISSS) versus stop smoking support alone on body weight in quitters: results of a randomised controlled trial
Source: BMJ Open. 2020 Jan 26;10(1):e032271. doi: 10.1136/bmjopen-2019-032271 (PMC7045045; doi:10.1136/bmjopen-2019-032271)

**Supplementary Figure 1. Reasons for declining to take part in SWISS (more than 1 choice possible)**

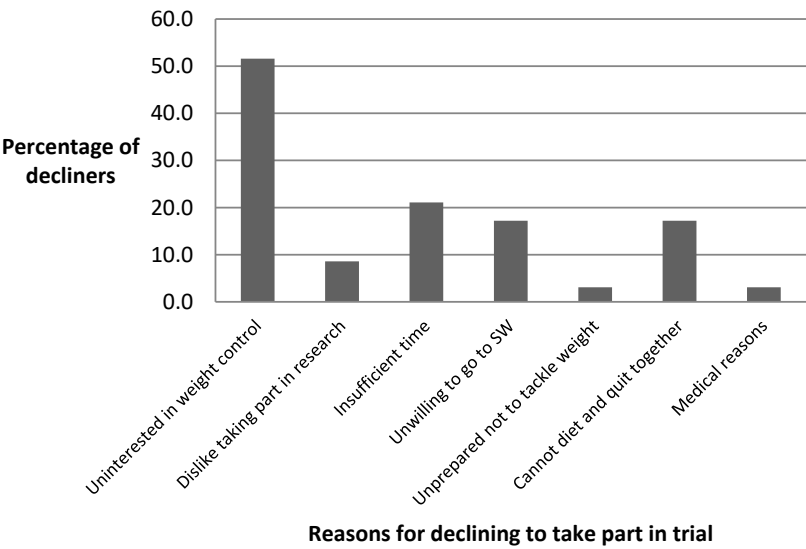

Supplement: Supplementary data [file bmjopen-2019-032271supp001.pdf]
