# Supplementary material for: Referral to Slimming World in UK Stop Smoking Services (SWISSS) versus stop smoking support alone on body weight in quitters: results of a randomised controlled trial
Source: BMJ Open. 2020 Jan 26;10(1):e032271. doi: 10.1136/bmjopen-2019-032271 (PMC7045045; doi:10.1136/bmjopen-2019-032271)
Supplement: Supplementary data [file bmjopen-2019-032271supp002.pdf]

Code (number of times featured in data)	Sub theme	Theme
Good trial (2)	Positive aspects	Aspects related to research processes
Easy to implement (4)		
Additional [paper] work (3)	Time and additional effort on part of practitioner and patient	
Patient time (2)		
Practitioner time (7)		
Discussion of research viewed negatively (1)	Engaging in research was viewed positively and negatively	
Discussion of research viewed positively (1)		
Patient disappointment with randomisation (3)	Disappointment for participants and practitioners (and misunderstanding for practitioner) regarding randomisation	
Practitioner disappointment with randomisation (2)		
Slimming World vouchers seen as an positive incentive (2)		
Discussion of weight viewed negatively (1)	Discussing weight was seen as a positive and negative thing	An opportunity to address the weight issues
Discussion of weight viewed positively (5)		
Lack of patient interest in weight (4)	Patient interest and attendance	Poor participant interest and attendance
Patient drop out, transient population (3)		
Few people wanting to quit (2)		

Supplementary Table 1. Content analysis of open comments from practitioners at the end of the trial
