# Supplementary material for: Referral to Slimming World in UK Stop Smoking Services (SWISSS) versus stop smoking support alone on body weight in quitters: results of a randomised controlled trial
Source: BMJ Open. 2020 Jan 26;10(1):e032271. doi: 10.1136/bmjopen-2019-032271 (PMC7045045; doi:10.1136/bmjopen-2019-032271)
Supplement: Author's manuscript [file bmjopen-2019-032271.draft_revisions.pdf]

BMJ Open is committed to open peer review. As part of this commitment we make the peer review history of every article we publish publicly available.

When an article is published we post the peer reviewers' comments and the authors' responses online. We also post the versions of the paper that were used during peer review. These are the versions that the peer review comments apply to.

The versions of the paper that follow are the versions that were submitted during the peer review process. They are not the versions of record or the final published versions. They should not be cited or distributed as the published version of this manuscript.

BMJ Open is an open access journal and the full, final, typeset and author-corrected version of record of the manuscript is available on our site with no access controls, subscription charges or pay-per-view fees (http://bmjopen.bmj.com).

If you have any questions on BMJ Open's open peer review process please email editorial.bmjopen@bmj.com

BMJ Open

Slimming World in Stop Smoking Services (SWISSS): Results of a randomized controlled trial

Journal:	*BMJ Open*
Manuscript ID	bmjopen-2019-032271
Article Type:	Research
Date Submitted by the Author:	11-Jun-2019
Complete List of Authors:	Lycett, Deborah; Coventry University<br>Aveyard, Paul; University of Oxford, Primary Care Health Sciences<br>Farmer, Andrew; University of Oxford, Department of Primary Care Health Sciences<br>Lewis, Amanda; University of Bristol, Population Health Sciences Bristol Medical School<br>Munafo, Marcus; University of Bristol, Experimental Psychology
Keywords:	weight, smoking, obesity

SCHOLARONE™
Manuscripts

Slimming World in Stop Smoking Services (SWISSS): Results of a randomized controlled trial

Deborah Lycett PhD[1*]

[*] Corresponding author

Email: deborah.lycett@coventry.ac.uk

Paul Aveyard PhD[2]

Email: paul.aveyard@phc.ox.ac.uk

Andrew Farmer DM[2]

Email: andrew.farmer@phc.ox.ac.uk

Amanda Lewis PhD[3]

Email: amanda.lewis@bristol.ac.uk

Marcus R. Munafò PhD[4]

Email: marcus.munafo@bristol.ac.uk

[1] Faculty of Health & Life Sciences, Coventry University, Priory Street, Coventry CV1 5FB, UK. [2] Nuffield Department of Primary Health Care Sciences, University of Oxford, Radcliffe Observatory Quarter, Woodstock Road, Oxford OX2 6GG, UK. [3]Population Health Sciences Bristol Medical School, University of Bristol, Canynge Hall (Room G.08a), 39 Whatley Road Bristol, BS8 2PS. [4]MRC Integrative Epidemiology Unit, UK Centre for Tobacco and Alcohol Studies, School of Experimental Psychology, University of Bristol, 12a Priory Road, Clifton, Bristol BS8 1TU, UK.

For peer review only

ABSTRACT

Introduction

Most people who stop smoking gain weight. Dietary modification may seem an obvious solution, but food restriction may increase cigarette craving and smoking relapse.

Trial Design

An unblinded parallel randomised controlled trial

Methods

Participants were adult smokers with a body mass index (BMI) greater or equal to 23 kg/m^2.

Setting was NHS commissioned Stop Smoking Services,

Interventions were referral to a commercial weight management programme, plus stop smoking support (treatment group), compared to stop smoking support alone (control group).

Objective was to compare weight change between interventions in smoking abstainers and not abstinent rates in all.

Primary outcome was change in weight (kg) at 12 weeks.

Randomisation sequence was computer generated and concealed until allocation.

Results

Seventy-six participants were recruited, 37 were randomized to the treatment group and 39 to the control group.

For peer review only

Change in weight was analysed in long-term abstainers (13 treatment, 14 control) only because the aim was to prevent weight gain associated with smoking cessation.

Abstinence was analysed on an intention-to-treat basis (37 treatment, 39 control).

At 12 weeks weight gain was less in the treatment than the control group with a mean difference (adjusted for baseline) of -1.9kg 95% CI [-3.4, -0.4]. Craving scores were lower (MPSS-C -1.4 [-2.4, -0.5]) and quit rates were higher (1.8 [0.7, 5.3]) in the treatment than the control group, although the trial was not powered to superiority in cravings and quit rates. Hunger ratings were similar in both groups across.

No adverse events or side effects were reported.

Conclusion

In people who are obese and want to quit smoking, these data provide modest encouragement that providing weight management at the time of quitting may be helpful. Those who are not obese, but who are informed about potential weight gain during their quit attempt, were uninterested in a weight management programme.

TRIAL REGISTRATION

Current Controlled Trials ISRCTN65705512

http://www.controlled-trials.com/ISRCTN65705512

For peer review only

ARTICLE SUMMARY

Strengths and limitations of this study

- The first study investigating referral to a commercial weight loss programme during smoking cessation.
- Computer generated randomisation with allocation concealment.
- Weight measured and abstinence verified by exhaled CO.
- Small study with poor recruitment but reverse power calculation showed greater than 80% power to detect change in primary outcome of weight.

INTRODUCTION

Weight gain is a well-known consequence of smoking cessation [1, 2]. It may deter smokers from attempting to quit and offsets some advantages of giving up smoking. Smoking cessation-related weight gain partly explains the finding that the incidence of type 2 diabetes is increased by up to 73% in the years after cessation [3-5], there is a 30% increased risk of hypertension [6], and a deterioration in glycaemic control in those with diabetes [7] who quit compared to those who continue to smoke. Despite these concerns weight gain in quitters does not negate the risk reduction of cardiovascular morbidity and mortality in those who successfully quit smoking [8]. There is some evidence to suggest the benefit may be mitigated [9] but the number of incident cases in higher weight gain categories are too small to draw conclusions. Nonetheless many people find this weight gain unacceptable and the greatest danger is it may deter individuals from quitting [10]. Yet, restricting food intake may

For peer review only

limit successful quitting as hunger increases urges to smoke [11, 12] and people who gain most weight quit more successfully [13].

An individually tailored plan to reduce energy intake and increase exercise, with regular monitoring and adaptation of individual goals, appears to be the most promising way to prevent cessation related weight gain with the least risk of impeding a quit attempt [14]. Commercial weight management providers (CWMPs) offer this type of support and are an effective way for many to lose weight through referral from National Health Service (NHS) primary care [15]. Therefore, referral to a CWMP on prescription from the NHS Stop Smoking Services may be an effective way to prevent smoking-related weight gain without a detrimental effect on quitting success.

METHODS

Design

This was a parallel group, individually randomized controlled trial (RCT) to compare standard stop smoking behavioural support with an intervention that, in addition to providing standard stop smoking support, included personalised weight management support, provided by Slimming World.

This was an open label trial, as blinding patients and smoking cessation advisors to allocation to intervention or control was impossible. The primary outcome was objectively measured weight and therefore the scope for bias is limited. Stop smoking advisors, who weighed participants, did not provide the weight control support and were unlikely to have a vested interest in interpreting weight change favourably. Full details of the methods for trial can be found in the protocol paper [16] but are summarized below.

For peer review only

Setting

NHS commissioned Stop Smoking Services were first recruited from Bristol and North Somerset Primary Care Trusts (PCTs). Stop smoking services were invited to take part by letter and a follow-up phone call 2 weeks later. Clinics were only eligible to participate if they had 2 new service users coming through their doors each week. Due to poor recruitment we invited more clinics (n=30) to take part in the study by widening our area further across the West Midlands and South-West England. Additionally we extended the planned duration of recruitment from 10 to 17 months, which was as far as our budgetary restraints would allow.

All individuals attending the stop smoking services were invited to take part in the trial at their first visit when they set their quit date. At the second visit those wanting to participate in the trial gave their consent and were randomised to the control or intervention group. Those randomised to the intervention were given a referral to Slimming World, a location and a start date as near to their quit date. Those who declined to take part were invited to provide a reason.

Baseline measures were taken after randomization and included age, gender, socioeconomic status, ethnicity, religion, Fagerström Test for Cigarette Dependence (FTCD), EQ-5D (EuroQol, Rotterdam, Netherlands) and measure of health-related quality of life.

For peer review only

Participants

Daily adult smokers with expired CO ≥10 ppm and a body mass index (BMI) greater or equal to 23 kg/m^2 were included. Pregnant smokers, those with a BMI < 23kg/m^2, those currently losing weight, or where weight loss was contraindicated, were excluded.

Randomization

A randomization sequence was generated using computer software. Stratified randomisation by stop smoking advisor with blocking within each stratum was used to ensure balance. The blocks were randomly ordered blocks of 2, 4 and 6. Participants were randomised 1:1 to usual care or Slimming World with usual care. Stop smoking advisors were unaware of the randomisation sequence; they opened sealed, numbered, opaque envelopes in turn.

Interventions and comparisons

Trial treatment providers

Trained NHS stop smoking advisors provided participants with standard smoking cessation support in both arms of the trial. Weight control support was provided by trained weight management counsellors employed by Slimming World. Slimming World is a CWMP which follows the National Institute for Health and Care Excellence (NICE) criteria for clinical practice. It is commissioned by the NHS to provide a weight loss service to patients. Slimming World was the CWMP of choice as it works with populations to prevent excessive weight gain rather than solely focusing on weight loss, this was pertinent to preventing cessation related weight gain.

For peer review only

Intervention

In addition to usual care, participants attended Slimming World for 12 weeks receiving support to lose weight or prevent weight gain. Slimming World consultants typically agree weight management targets with their members at the first appointment and then work with their members to achieve this through regular weight monitoring, controlled amounts of high energy foods and personal eating plans.

Control

Participants were encouraged to quit smoking first, before tackling weight. Stop smoking advisors advised satiating hunger with healthy foods, which is standard care, but did not provide detailed or personalized advice regarding weight management.

Outcome measures

Primary outcome measure:

Change in measured weight from baseline to 12 weeks post-quit day in abstinent smokers. Abstinence was prolonged abstinence, defined according to the Russell Standard [17].

Secondary outcome measures:

- Weight at 4 weeks post-quit date.
- Weight at 6 months from baseline.
- Abstinence at 4, 12 and 26 weeks
- Participant acceptability measured quantitatively by response, attrition rates and a decliner's reasons for not participating.

For peer review only

- Feasibility of running the trial within the Stop Smoking Services was measured by an open text questionnaire asking for comment.

Exploratory outcomes measures:

- Change in EQ-5D.
- Cigarette withdrawal symptom scores as measured by the mood and physical symptoms scale (MPSS) [18].
- Associations of change in religious engagement (adapted from CSI-MEMO [19]) and religious coping (religious coping index (RCI) [20]) as positive use of individual's spiritual reserve to change weight [21] and smoking behaviour [22] is increasingly evidenced.

Statistics and data analysis

Sample size

We did not reach our planned sample size but reverse power calculation showed that we had >80% power, at an alpha error rate of 0.05, to detect the observed differences in weight change in our sample at 12 weeks between the treatment and control groups. Given a large observed difference in quit rates between the control and treatment groups we also had 90% power at an alpha error rate of 0.05 to determine non-inferiority, using a non-inferiority limit of 19% [23].

Analysis

The change in weight was analysed in long-term abstainers only because only long-term abstainers gain more weight over time than the general population and because the aim was to prevent weight gain on smoking cessation [24]. Weight change in the intervention and the control arm of the study was presented descriptively using means and SDs and the mean

For peer review only

difference and 95% confidence intervals (CI) between the two arms calculated using ANCOVA, with baseline values used as a covariate. This accounts both for baseline differences and regression to the mean effects [25].

Abstinence was analysed on an intention-to-treat basis, assuming all those not present at follow-up had relapsed [17]. As we recruited fewer people than intended, we refrained from testing the significance of the differences.9

The quality of life (EQ-5D) was presented as index values calculated from interim scoring for the EQ-5D-5L spreadsheet for UK values [26], means and SD of the EQ-5D visual analogue scale (VAS) were also presented following the EQ-5D-5L reporting guide.

The change in exploratory measures were presented with descriptive statistics and analysed using ANCOVA as described for weight change. We also reported descriptive data about the acceptability of the intervention, supplemented by comments from questionnaires completed by stop smoking service practitioners. We analysed these using manifest content analysis of a broad surface structure reflecting what was literally said [27]. Open comments were broken down into individual codes of meaning then built up into sub themes and themes which summarised the data.

For peer review only

Data statement

Please contact the corresponding author if you would like to access the trial data.

Ethics approval

The study was approved by Cornwall and Plymouth NHS Research Ethics Committee. The trial was conducted in accordance with the recommendations for physicians involved in

research on human subjects adopted by the 18th World Medical Assembly, Helsinki, 1964, and later revisions. All participants gave their informed consent to participate in the study.

Patient and Public Involvement

A patient advisory group were asked to comment on the concept of the study and the perceived acceptability of the proposed intervention, feedback was positive and so study plans progressed.

Participant reimbursement for attending follow-up visits

Participants received £5 payment for travel and inconvenience at the 12 and 26 week follow-up visits.

RESULTS

Potential Participants

Thirty different stop smoking clinics in the West Midlands and South-West of England inclusion criterion of treating at least two new service users attending a clinic each week. Potentially over 4400 participants could have been accessed over our 17 month recruitment period and invited to take part in the trial. Of those who declined to take part 128 individuals provided us with a reason. Eighty-three people agreed to take part and were screened for eligibility, 7 were ineligible (4 were already losing weight through attending a commercial weight loss provider) and 76 individuals were randomised. Of these, 37 were randomized to the treatment group and 12 returned for the 26 week follow-up, while 39 were randomized to the control group of which 10 returned for the 26 week follow-up. The majority of those not responding to follow-up did so in the first four weeks of the trial and continued to ignore requests thereafter. Eight withdrew from the trial for various reasons, one individual in the

For peer review only

control group did so to join Slimming World and two individuals in the intervention group said it was too difficult to quit and diet at the same time, another reported returning to smoking (Figure 1).

Characteristics of 'decliners' and participants

Those who provided a reason for declining to take part (decliners) and those who agreed to participate were of a similar mean age and mostly white British people. Women were more likely than men to enrol in the trial, but the most striking difference related to baseline weight status. Practically everyone who was obese agreed to join the trial, while a minority in the other BMI groups did so (Table 1).

Over half those who declined to take part did so because they were not interested in weight control. (Proportionally more who gave this reason had a healthy weight rather than being overweight (data not shown).) Over 20% of reasons for declining were because people did not have time. 17% said they were not prepared to go to Slimming World, similar numbers said they could not quit smoking and diet at the same time. Medical reasons and reasons related to the research process such as the principle of taking part or being randomised to the control were not commonly reported (Supplementary Figure 1).

Baseline Characteristics of participants in the treatment and control group at baseline

Baseline characteristics of participants were similar in the control and treatment groups (Table 2).

Weight Change During Abstinence

Among abstinent participants, there was a steady mean weight gain over time in the control group, whereas weight reduced at each time point in the treatment group. In those who were quit at 4 week follow-up there was an adjusted mean difference of -1.2kg [-2.5, 0.5] weight

For peer review only

change between those in the intervention group and those in the control group. At 12 weeks the difference was greater -1.9kg [-3.4, -0.4] and some of this initial difference in weight loss between the trial arms was maintained at 26 week follow-up (-1.3kg [-2.7, 0.1]). (Table 3).

Change in Abstinence (Intention-to-treat analysis)

In the control group 13 of 39 participants (34%) were abstinent at 4 weeks and in the treatment group 14 of 37 (38%) were abstinent. By 12 weeks 8 (21%) participants in the control and 12 (32%) in the treatment group remained abstinent. At 26 week follow-up 5 (13%) participants in the control group remained abstinent and 8 (22%) in the treatment group.

Change in Exploratory Outcomes

Change in the MPSS craving domain (MPSS-C) at all time-points showed a greater reduction in the treatment than the control group, with mean adjusted differences ranging from 0.7 to 1.4 on a scale of 0-10. In the mood domain, MPSS-M, in the longer term (26 week follow-up) there was a greater reduction in negative affect in the treatment group than the control group but the adjusted mean difference was negligible -1.3 [-3.0, 0.6] on a scale between 7 and 35. The effect on hunger and MPSS-P within and between the trials arms was negligible at all time-points. There was negligible change in religious coping and the Index Values of EQ-5D between the trial arms. The visual analogue scale (VAS) for quality of life improved between 4-6% in both groups across all time points (Table 4).

Practitioners' comments on trial delivery

For peer review only

Three main themes came out of the content analysis of practitioners' comments, these were 'Aspects related to research processes', which included positive aspects such as ease of implementation, however additional time and paperwork particular on the part of the practitioner, and also the perceived burden for the participant, were viewed negatively. Randomisation to the control group disappointed both the participant and the practitioner; practitioners reported viewing Slimming World vouchers as an incentive and this suggested misunderstanding of the nature and value of randomization and the need to promote the control arm as a good alternative. Such attitudes may have fuelled disappointment and subsequent drop out. The second theme was 'An opportunity to address weight issues', this opportunity was predominantly seen as a positive aspect of the trial and practitioners anticipated this would generate great interest from participants. However, practitioners were somewhat surprised that participants did not seem to want to address weight, there was mention that fewer people at the time of this trial were seeking stop smoking services than expected, and high rates of drop out were also problematic, these comments led to the third theme 'Poor participant interest and attendance' which characterised experience of delivering the trial (Supplementary Table 1).

DISCUSSION

Summary of results and consistency with other findings

Referral to the 12-week Slimming World programme plus usual stop smoking support achieved significantly less weight gain than usual stop smoking support alone. This difference (-1.9kg[-3.4, -0.4]) was apparent at 12 weeks post-quit and is slightly greater than that seen in other trials comparing personalised weight management to usual care (-1.1kg [-1.9, -0.3]) [14]. This was partially sustained at 26 weeks post-quit (-1.3kg [-2.7, 0.1]). However, we were unable to recruit to our target sample size and these results lack precision. Percentage quit was no worse

For peer review only

in the treatment than the control group; it was slightly higher in the treatment group but the trial was underpowered to test superiority. MPSS craving scores dropped further in the treatment than the control group (>1point difference on 10point scale at 12 and 26 weeks). Hunger ratings were similar in both groups across all time points.

Comparing our findings with the meta-analyses of personalised weight management versus no weight management at 26 weeks [14], our study achieved a similar quit rate in our treatment group (22%) as in the control groups of other studies (21%). However the quit rates in our control group was lower than in these other studies (13% vs 19%) which may account for the difference in quit rates we saw here.

Taken together these results suggest that referral to a commercial weight loss provider, such as Slimming World, at the time of a quit attempt can prevent cessation-related weight gain without negatively impacting a quit attempt. However, we cannot draw firm conclusions given our small sample size.

Limitations

The greatest limitation to this study was the difficulty in recruiting which led to the small sample size. Anecdotally stop smoking advisors often cite weight gain is raised as a concern in clinics and were anticipating a high uptake for this trial, however we found that when given the opportunity participation was poor and more than 50% of people who gave a reason for declining reported it was because they were 'uninterested in weight control'.

While we do not know the reasons for everyone who declined, these results suggest that weight gain may not be as much as a barrier to quitting smoking as is commonly believed, particularly in those who are not obese. However all those who were invited to participate in the trial had already decided to quit smoking and were looking for support in that goal. In addition, people

For peer review only

who were already losing weight or attending a commercial weight loss programme were ineligible to take part (9% the 44 screened for eligibility). The Office for National Statistics reports weight gain only deters 5% of people from quitting and only 3% of people cite it as a reason for returning to smoking [28]. In light of this, and in view of the evidence that the cardiovascular benefits of quitting are evident despite weight gain [8, 9], we can reassure quitters who are worried about gaining weight that they need not be unduly concerned. In fact, evidence from a randomised control trial in the US (350 quitters at 6 months) suggests that counselling quitters to reduce their weight concerns (through CBT telephone counselling compared to standard quit advice) leads to similar rates of quitting and a significant difference in weight change in favour of the counselling group (-0.5kg vs 1.0kg). Such counselling involved identifying and addressing maladaptive thoughts about body shape and weight, discouraged restrictive dieting, educated quitters on the relationship between weight gain and smoking cessation and encouraged an acceptance of moderate weight gain after quitting [29].

Another limitation is the high rate of loss to follow-up, in smoking cessation trials this is usually due to participants returning to smoking and as such the standard approach in analysis is to assume this [17]. However, our trial showed higher loss to follow-up compared to other smoking cessation trials [22], this may have been due to staff time pressures reported by practitioners which may have limited their continued attempts to make contact with those who did not attend.

Our trial did show that a few people were interested in preventing weight gain by attending a behavioural weight loss programme, and those who wanted to take part in the trial were more likely to be female and more likely to be obese. This is consistent with other findings that suggest women who are obese are likely to be the most weight concerned [30].

For peer review only

For individuals who feel unable to make a quit attempt without concurrently controlling their weight, this trial suggests that referral to a behavioural weight loss programme may be a pragmatic option within stop smoking clinics.

Implications for future trials to prevent cessation related weight gain:

- Stop smoking service practitioners consider a relatively straightforward trial delivered with support to impose undue burdens on their time, limiting their willingness to approach potential participants. This is mostly due to the paperwork concerned with consent and follow-up. In low-intensity low-risk trials, we need to develop a method for achieving this that does not impose the same burdens as trials of much riskier interventions. In the meantime, triallists need to understand this and make fewer demands by redesigning the trials.
- Most people who are stopping smoking are uninterested in active behavioural support to prevent weight gain. However, where people already are overweight to the extent of being obese, most people were prepared to enter the trial and future trials may target this population.
- Practitioners and participants quickly become despondent if not randomised to their choice of intervention, almost always the intervention group. More intensive training for practitioners in how to present trials to create equipoise is likely to be needed. There was a suggestion that quit rates were higher in the intervention than the control group and it may be helpful to explore qualitatively whether unhappiness at weight gain explains this.

Conclusion

For peer review only

Referral to the 12-week Slimming World programme plus usual stop smoking support achieved weight loss compared with weight gain seen with usual smoking cessation support alone throughout the quit attempt as far as 26 weeks. Quit rates, cravings for cigarettes, feelings of hunger and quit rates did not seem to be adversely affected although the recruited sample was too small to confirm this.

Recruitment into a trial of stop smoking services referred into a behavioural weight loss programme was poor. They most common reason for declining to participate was no interest in controlling weight.

ABBREVIATIONS

BMI: body mass index; CI: confidence interval; CRF: case report form; CWMP: commercial weight management providers; FTCD: Fagerström Test for Cigarette Dependence; GCP: Good Clinical Practice; ICH GCP: International Conference on Harmonisation Good Clinical Practice; LES: local enhanced services; MPSS: mood and physical symptoms scale; NIHR: National Institute for Health Research; NHS: National Health Service; NICE: National Institute for Health and Care Excellence; PC-CRTU: Primary Care Clinical Research and Trials Unit; R&D: Research and Development; RCI: religious coping index; RCT: randomized controlled trial; REC: research ethics committee; SOP: standard operating procedure; SPCR: School for Primary Care Research; UKCTCS: UK Centre for Tobacco Control Studies.

COMPETING INTERESTS

DL has received hospitability from manufacturers of smoking cessation products, Pfizer Ltd, Tadworth, UK. DL's institution during the active phase of the trial has received smoking

For peer review only

cessation products for use in a clinical trial from Johnson & Johnson Ltd, New Brunswick, NJ, USA. DL has received Slimming World membership vouchers for use in this trial. DL has received expenses and consultancy fees from the NHS and Universities for teaching about cessation-related weight gain. DL has received grant funding from UKCTCS and the NIHR-SPCR for research relating to cessation-related weight gain. PA is an NIHR senior investigator and is funded by NIHR Biomedical Research Centre and the CLAHRC, Oxford. AF is an NIHR Senior Investigator and receives funding from NIHR Oxford Biomedical Research Centre. AL has received hospitality from Weight Watchers. In the last 5 years, MM has received grant funding from Pfizer Ltd and varenicline, for research purposes.

AUTHOR CONTRIBUTIONS

DL designed the study and drafted the manuscript. AP, AF, AL and MM contributed to study design and manuscript revision. All authors read and approved the final manuscript.

FUNDING

This work was supported by funding of the salary for DL, provided by a fellowship from the NIHR-SPCR during the active trial period. Additional funds to set up the trial and buy equipment were provided by the NIHR-SPCR and UKCTCS. The UKCTCS is one of five UK Public Health Research Centres of Excellence. We also gratefully acknowledge funding from the British Heart Foundation, Cancer Research UK, the Economic and Social Research Council, Medical Research Council and the Department of Health, under the auspices of the UK Clinical Research Collaboration. AL's salary was paid by NIHR-SPCR during the trial period, and the salaries of AF, MM and PA were paid by their respective institutions. PA is an NIHR senior investigator and funded by NIHR Oxford Biomedical Research Centre and CLAHRC. All funding is gratefully acknowledged.

For peer review only

ACKNOWLEDGEMENTS

We thank Carolyn Pallister and her colleagues at Slimming World for their cooperation and provision of the intervention free of charge to trial participants. We thank the Stop Smoking Services for their participation.

REFERENCES

1. Klesges RC, Winders SE, Meyers AW, et al: **How much weight gain occurs following smoking cessation? A comparison of weight gain using both continuous and point prevalence abstinence.** *J Consult Clin Psychol* 1997, **65**(2):286–291.

2. Klesges RC, Winders SE, Meyers AW, et al: **Smoking, body weight and their effects on smoking behavior: a comprehensive review of the literature.** *Psychol Bull* 1989, **106**:204–230.

3. Wannamethee SG, Shaper AG, Perry IJ: **Smoking as a modifiable risk factor for type 2 diabetes in middle-aged men.** *Diabetes Care* 2001, **24**(9):1590–1595.

4. Davey-Smith G, Bracha Y, Svendsen KH, Neaton JD, Haffner SM, Kuller LH; Multiple Risk Factor Intervention Trial Research Group: **Incidence of type 2 diabetes in the randomized multiple risk factor intervention trial.** *Ann Intern Med* 2005, **142**(5):313–322.

5. Yeh HC, Duncan BB, Schmidt MI, Wang NY, Brancati FL: **Smoking, smoking cessation, and risk for type 2 diabetes mellitus.** *Ann Intern Med* 2010, **152**(1):10–17.

6. Gerace TA, Hollis JF, Ockene JK, Svendsen KH: **Smoking cessation and change in diastolic blood pressure, body weight, and plasma lipids. MRFIT Research Group.** *Prev Med* 1991, **20**(5):602–620.

For peer review only

7. Lycett, D., Nichols, L., Ryan, R., et al. **The association between smoking cessation and glycaemic control in patients with type 2 diabetes: a THIN database cohort study**. *Lancet Diabetes Endocrinol* 2015. *3*(6), pp.423-430.

8. Clair, C., Rigotti, N.A., Porneala, B., et al. **Association of smoking cessation and weight change with cardiovascular disease among adults with and without diabetes.** *JAMA* 2013, 309(10), pp.1014-1021.

9. Luo J, Rossouw J, Margolis KL. **Smoking Cessation, Weight Change, and Coronary Heart Disease Among Postmenopausal Women With and Without Diabetes.** *JAMA*. 2013;310(1):94–96. doi:10.1001/jama.2013.6871

10. Brouwer RJN, Pomerleau CS: **"Prequit attrition" among weight-concerned women smokers.** *Eat Behav* 2000, **1**(2):145–151.

11. Cheskin LJ, Hess JM, Henningfield J, Gorelick DA: **Calorie restriction increases cigarette use in adult smokers.** *Psychopharmacology (Berl)* 2005, **179**(2):430–436.

12. Leeman RF, O'Malley SS, White MA, McKee SA: **Nicotine and food deprivation decrease the ability to resist smoking.** *Psychopharmacology (Berl)* 2010, **212**(1):25–32.

13. Hall SM, Tunstall CD, Vila KL, Duffy J: **Weight gain prevention and smoking cessation: cautionary findings.** *Am J Public Health* 1992, **82**(6):799–803.

14. Farley AC, Hajek P, Lycett D, Aveyard P: **Interventions for preventing weight gain after smoking cessation.** *Cochrane Database Syst Rev* 2012, **Jan 18**;1:CD006219.

For peer review only

15. Jolly K, Lewis A, Beach J, et al.: **Comparison of range of commercial or primary care led weight reduction programmes with minimal intervention control for weight loss in obesity: Lighten Up randomised controlled trial.** *BMJ* 2011, **343**:d7243.

16. Lycett, D., Aveyard, P., Farmer, A., Lewis, A. and Munafò, M. **Slimming World in Stop Smoking Services (SWISSS): study protocol for a randomized controlled trial**. *Trials*, 2013. *14*(1), p.182.

17. West R, Hajek P, Stead L, Stapleton J: **Outcome criteria in smoking cessation trials: proposal for a common standard.** Addiction 2005, 100(3):299–303.

18. West R, Hajek P: **Evaluation of the mood and physical symptoms scale (MPSS) to assess cigarette withdrawal.** Psychopharmacology 2004, 177:195–199.

19. Puchalski C, Romer AL: Taking a spiritual history allows clinicians to understand patients more fully. J Palliat Med 2000, 3:129–137.

20. Koenig HG, Cohen HJ, Blazer DG, et al.: Religious coping and depression among elderly, hospitalized medically ill men. Am J Psychiatry 1992, 149(12):1693–1700.

21. Lancaster, K.J., Carter ‐ Edwards, L., Grilo, S., Shen, C. and Schoenthaler, A.M.. Obesity interventions in African American faith ‐ based organizations: a systematic review. Obesity Reviews 2014, 15(S4), pp.159-176.

22. Gonzales D, Redtomahawk D, Pizacani B, et al.: Support for spirituality in smoking cessation: results form a pilot survey. Nicotine Tob Res 2007, 9(2):299–303.

For peer review only

23. Lindson-Hawley, N., Banting, M., West, R., Michie, S., Shinkins, B. and Aveyard, P., 2016. **Gradual versus abrupt smoking cessation: a randomized, controlled noninferiority trial.** Annals of internal medicine, 164(9), pp.585-592.

24. Lycett D, Munafò M, Johnstone E, Murphy M, Aveyard P. Associations between weight change over 8 years and baseline body mass index in a cohort of continuing and quitting smokers. Addiction. 2011 Jan;106(1):188-96.

25. Vickers AJ, Altman DG. Analysing controlled trials with baseline and follow up measurements. BMJ. 2001 Nov 10;323(7321):1123-4

26. Preloading Investigators. **Effects on abstinence of nicotine patch treatment before quitting smoking: parallel, two arm, pragmatic randomised trial**. *BMJ*, 2018. *361*, p.k2164.

27. Bengtsson M. How to plan and perform a qualitative study using content analysis, NursingPlus Open 2016, Volume 2, Pages 8-14, ISSN 2352-9008, https://doi.org/10.1016/j.npls.2016.01.001.

28. Lader D: **A report on research using the National Statistics Opinions Survey produced on behalf of the NHS Information Centre for health and social care**. Office for National Statistics 2009.

29. Bush T, Levine MD, Beebe LA, et al. **Addressing weight gain in smoking cessation treatment: a randomized controlled trial.** Am J Health Promot. 2012 Nov-Dec;27(2):94-102. doi: 10.4278/ajhp.110603-QUAN-238.

For peer review only

30. Levine MD, Bush T, Magnusson B, Cheng Y, Chen X; **Smoking-Related Weight Concerns and Obesity: Differences Among Normal Weight, Overweight, and Obese Smokers Using a Telephone Tobacco Quitline**, Nicotine Tob Res, 2013. Volume 15, Issue 6, 1 June 2013, Pages 1136–1140, https://doi.org/10.1093/ntr/nts226

For peer review only

Characteristic (mean (SD)/% frequency)	Control (n=39)	Treatment (n=37)
**Age (years)**	50 (15)	44 (12)
**BMI (kg/m^2)**	31.6 (6.3)	29.3 (4.3)
**FTND (scores range: 1-10)**	5.0 (2.2)	6.4 (5.7)
**MPSSM (scores range 7-35)**	12.7 (5.7)	12.8 (4.1)
**MPSSC (scores range 0-10)**	6.9 (1.7)	7.3 (1.1)
**MPSSP (scores range 3-15)**	4.2 (1.8)	3.9 (1.1)
**MPSST (scores range 10-60)**	23.8 (7.3)	24.1 (4.9)
**EQ-5D Index**	0.759 (0.268)	0.848 (0.105)
**Value**	61 (23)	67 (15)
**VAS (100 point scale)**		
**Using religion to cope score (scores range 1-10)**	(n=23) 2.7 (2.6)	(n=21) 3.7 (2.9)
**Gender**	(n=38)	(n=33)
**Male**	29%	44%
**Female**	71%	56%
**Ethnicity White**	92%	84%
**British**	5%	
**White & Asian**		3%
**Indian**	3%	13%
**Unknown**		
**Highest Qualification**	(n=38)	(n=32)
**No formal qualification**	18%	25%
**GCSEs/O levels**	21%	28%
**A levels**	42%	31%
**Degree**	13%	9%
**Higher Degree**	6%	7%
**Employment**	(n=38)	(n=32)
**Employed**	55%	63%
**Long term sick**	11%	0%
**Looking after others**	3%	6%
**Retired**	21%	13%
**Unemployed**	8%	13%
**Other**	2%	5%
**Religious**	(n=37)	(n=30)
**No**	95%	87%
**Yes**	5%	13%
**Supportiveness of religious community**	(n=20)	(n=14)
**A lot**	5%	35%
**A little**	15%	25%
**Not at all**	80%	30%
**Impact of religious belief**	(n=11)	(n=10)
**Comfort**	91%	100%
**Stress**	9%	0%
**Family history of diabetes**	(n=36)	(n=31)
**Yes**	25%	26%
**No**	75%	74%
**Impaired glucose tolerance**	16%	19%
**Yes**	84%	81%
**No**		
**Absolute Diabetes Prevalence**	(n=3)	(n=32)
**Yes**	14%	13%
**No**	86%	87%

GCSE: General Certificate of Secondary Education, O level: Ordinary level exams, A level: Advanced level exams.

Table 1. Baseline characteristics by randomisation

For peer review only

Characteristics		Decliners (n=128)	Participants (n=76)
**Age**		45.3 (14.1)	46.7 (13.5)
**Ethnicity (%)**			
**White British**		93% (n=119)	88% (n=67)
**White Irish**		1% (n=1)	
**White other**		2% (n=3)	
**Indian**		2% (n=2)	1% (n=1)
**White & Asian**			3% (n=2)
**Black – other**		1% (n=1)	8% (n=6)
**Unknown**		1% (n=2)	
**Gender**			(n=71)
	**Female**	55% (n=70)	65% (n=46)
	**Male**	45% (n=58)	35% (n=25)
**BMI category**		(self-report) (n=123)	(measured) (n=73)
	**Underweight**	7% (n=8)	0% (n=0)
	**Healthy Weight**	58% (n=71)	12% (n=9)
	**Overweight**	35% (n=43)	34% (n=25)
	**Obese**	1% (n=1)	53% (n=39)

Table 2. Characteristics of those who declined to take part and those who participated in the SWISSS trial

Change in weight (kg) from baseline in quitters at:	Control	Treatment	Difference in weight change (treatment – control) mean [95% CI] ANCOVA adjusted for baseline weight as covariate
**4 weeks post quit**	(n=13)<br>0.7 (1.5)	(n=14)<br>-0.7 (1.8)	-1.2 [-2.5, 0.5]
**12 weeks post quit**	(n=8)<br>2.0 (1.9)	(n=12)<br>-1.3 (3.3)	-1.9 [-3.4, -0.4]
**26 weeks post quit**	(n=5)<br>2.3 (2.2)	(n=8)<br>-0.9 (2.5)	-1.3 [-2.7, 0.1]

Table 3. Change in weight in quitters at 4 weeks post quit

For peer review only

Change in Outcome from baseline (ITT-BOCF i.e. assumed no change)	At 4 weeks			At 12 weeks			At 26 weeks		
	**Control**	**Treatment**	**Difference in change (treatment – control) OR / mean [95% CI]***	**Control**	**Treatment**	**Difference in change (treatment – control) % / mean [95% CI]***	**Control**	**Treatment**	**Difference in change (treatment – control) % / mean [95% CI]***
**Abstinence rate**	13/39 (34%)	14/37 (38%)	1.2 [0.5, 3.1]	8/39 (21%)	12/37(32%)	1.8 [0.7, 5.3]	5/39 (13%)	8/37 (22%)	1.9 [0.6, 6.4]
**MPSS – C (range 0-10)**	-0.8 (1.5)	-1.5 (2.1)	-0.7 [-1.6, 0.2]	-0.7 (1.8)	-2.2 (2.7)	-1.4 [-2.4, -0.5]	-0.7 (1.5)	-1.9 (2.5)	-1.2 [-2.2, -0.3]
**MPSS – P (range 3-15)**	0.2 (1.3)	0.1 (1.0)	-0.2 [-0.7, 0.3]	<0.1 (1.6)	-0.1 (1.2)	-0.1 [-0.7, 0.5]	<0.1 (1.4)	-0.1 (1.1)	-0.3 [-0.9, 0.3]
**MPSS- M (range 7-35)**	1.1 (3.8)	0.9 (4.9)	-0.1 [-2.1, 1.8]	-0.5 (3.7)	-1.4 (4.8)	-0.2[-2.0, 1.6]	-0.6 (3.6)	-1.7 (5.0)	-1.3 [-3.0, 0.6]
**Hunger (range 1-5)**	0.2 (0.8)	0.1 (0.9)	<0.1 [-0.4, 0.4	0.1 (0.6)	0.1 (0.9)	<-0.1 [-0.4, 0.3]	0.1 (0.5)	0.1 (0.9)	<0.1 [-0.3, 0.4]
**MPSS- T (range 10-60)**	0.5 (4.4)	-0.6 (6.0)	-1.1[-3.6, 1.4]	-1.3 (5.0)	-3.7 (7.4)	-1.8 [-4.4, 0.9]	-7.1 (13.4)	-7.8 (14.5)	-2.8 [-5.5, -0.1]
**EQ-5D Index Value**	0.008(0.065)	0.009(0.112)	0.001 [-0.046, 0.047]	0.0165(0.086)	0.023(0.099)	0.006 [-0.042, 0.054]	0.001 (0.069)	0.023(0.078)	0.022 [-0.016, 0.060]
**VAS (100 point scale)**	5.5 (20.9)	4.4 (16.2)	-0.1 [-6.8, 6.7]	4.8 (12.7)	5.6 (12.7)	-1.8 [-4.7, 8.2]	5.7 (15.2)	4.8 (11.8)	1.5 [-5.1, 8.0]
**Using religion to cope score (range 1-10)**	-0.3 (1.2)	-0.8 (1.9)	-0.5 [-1.6, 0.6]	0.2 (0.5)	-0.3 (0.9)	-0.4[-1.6, 0.8]	0.5 (1.2)	<0.1 (1.8)	-0.4 [-1.6, 0.8]

*ANCOVA adjusted for baseline weight as covariate

Table 4. Change in abstinence and exploratory outcomes within and between the control and intervention group

Figure 1. Flow of participants through the trial

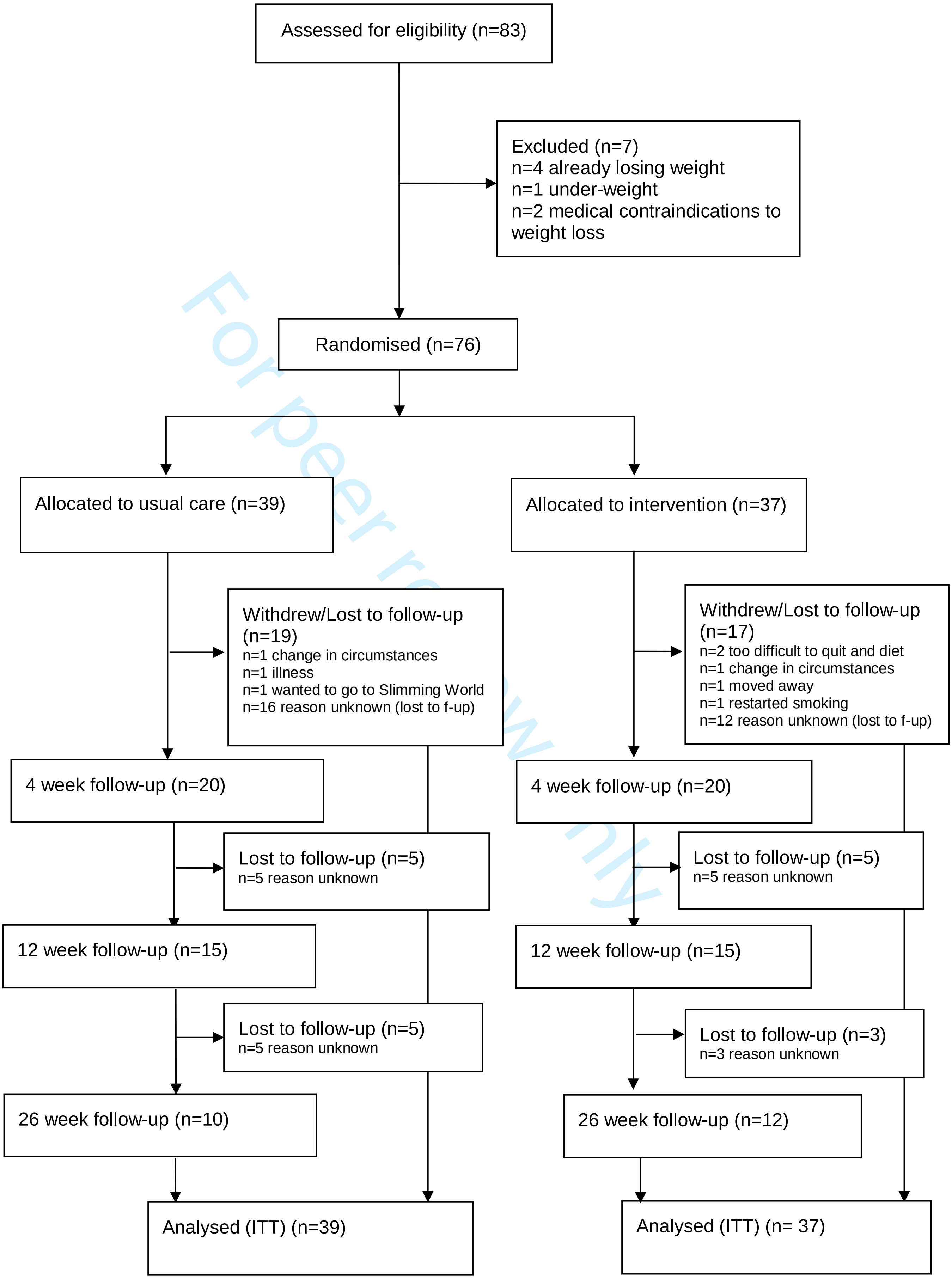

For peer review only

Supplementary Figure 1. Reasons for declining to take part in SWISSS (more than 1 choice possible)

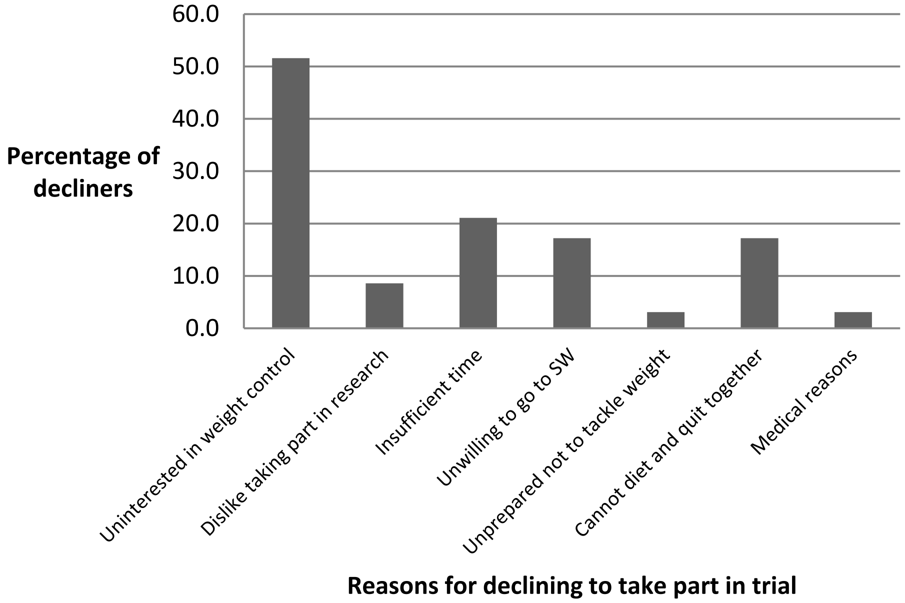

For peer review only

Code (number of times featured in data)	Sub theme	Theme
Good trial (2)	Positive aspects	Aspects related to research processes
Easy to implement (4)		
Additional [paper] work (3)	Time and additional effort on part of practitioner and patient	
Patient time (2)		
Practitioner time (7)		
Discussion of research viewed negatively (1)	Engaging in research was viewed positively and negatively	
Discussion of research viewed positively (1)		
Patient disappointment with randomisation (3)	Disappointment for participants and practitioners (and misunderstanding for practitioner) regarding randomisation	
Practitioner disappointment with randomisation (2)		
Slimming World vouchers seen as an positive incentive (2)		
Discussion of weight viewed negatively (1)	Discussing weight was seen as a positive and negative thing	An opportunity to address the weight issues
Discussion of weight viewed positively (5)		
Lack of patient interest in weight (4)	Patient interest and attendance	Poor participant interest and attendance
Patient drop out, transient population (3)		
Few people wanting to quit (2)		

Supplementary Table 1. Content analysis of open comments from practitioners at the end of the trial

For peer review only

CONSORT 2010 checklist of information to include when reporting a randomised trial*

Section/Topic	Item No	Checklist item	Reported on page No
**Title and abstract**			
	1a	Identification as a randomised trial in the title	1
	1b	Structured summary of trial design, methods, results, and conclusions (for specific guidance see CONSORT for abstracts)	2-3
**Introduction**			
Background and objectives	2a	Scientific background and explanation of rationale	4
	2b	Specific objectives or hypotheses	5
**Methods**			
Trial design	3a	Description of trial design (such as parallel, factorial) including allocation ratio	5
	3b	Important changes to methods after trial commencement (such as eligibility criteria), with reasons	N/A
Participants	4a	Eligibility criteria for participants	6
	4b	Settings and locations where the data were collected	5
Interventions	5	The interventions for each group with sufficient details to allow replication, including how and when they were actually administered	6-7
Outcomes	6a	Completely defined pre-specified primary and secondary outcome measures, including how and when they were assessed	7
	6b	Any changes to trial outcomes after the trial commenced, with reasons	N/A
Sample size	7a	How sample size was determined	9
	7b	When applicable, explanation of any interim analyses and stopping guidelines	9
Randomisation:			
Sequence generation	8a	Method used to generate the random allocation sequence	7
	8b	Type of randomisation; details of any restriction (such as blocking and block size)	7
Allocation concealment mechanism	9	Mechanism used to implement the random allocation sequence (such as sequentially numbered containers), describing any steps taken to conceal the sequence until interventions were assigned	7
Implementation	10	Who generated the random allocation sequence, who enrolled participants, and who assigned participants to interventions	7
Blinding	11a	If done, who was blinded after assignment to interventions (for example, participants, care providers, those	N/A

For peer review only - http://bmjopen.bmj.com/site/about/guidelines.xhtml

		assessing outcomes) and how	
	11b	If relevant, description of the similarity of interventions	N/A
Statistical methods	12a	Statistical methods used to compare groups for primary and secondary outcomes	9
	12b	Methods for additional analyses, such as subgroup analyses and adjusted analyses	9
**Results**			
Participant flow (a diagram is strongly recommended)	13a	For each group, the numbers of participants who were randomly assigned, received intended treatment, and were analysed for the primary outcome	Figure 1
	13b	For each group, losses and exclusions after randomisation, together with reasons	Figure 1
Recruitment	14a	Dates defining the periods of recruitment and follow-up	8
	14b	Why the trial ended or was stopped	9
Baseline data	15	A table showing baseline demographic and clinical characteristics for each group	Table 2
Numbers analysed	16	For each group, number of participants (denominator) included in each analysis and whether the analysis was by original assigned groups	Figure 1
Outcomes and estimation	17a	For each primary and secondary outcome, results for each group, and the estimated effect size and its precision (such as 95% confidence interval)	Table 3 and table 4
	17b	For binary outcomes, presentation of both absolute and relative effect sizes is recommended	Table 4
Ancillary analyses	18	Results of any other analyses performed, including subgroup analyses and adjusted analyses, distinguishing pre-specified from exploratory	9
Harms	19	All important harms or unintended effects in each group (for specific guidance see CONSORT for harms)	3
**Discussion**			
Limitations	20	Trial limitations, addressing sources of potential bias, imprecision, and, if relevant, multiplicity of analyses	15
Generalisability	21	Generalisability (external validity, applicability) of the trial findings	16
Interpretation	22	Interpretation consistent with results, balancing benefits and harms, and considering other relevant evidence	16
**Other information**			
Registration	23	Registration number and name of trial registry	3
Protocol	24	Where the full trial protocol can be accessed, if available	5
Funding	25	Sources of funding and other support (such as supply of drugs), role of funders	18

*We strongly recommend reading this statement in conjunction with the CONSORT 2010 Explanation and Elaboration for important clarifications on all the items. If relevant, we also recommend reading CONSORT extensions for cluster randomised trials, non-inferiority and equivalence trials, non-pharmacological treatments, herbal interventions, and pragmatic trials. Additional extensions are forthcoming: for those and for up to date references relevant to this checklist, see www.consort-statement.org.

For peer review only - http://bmjopen.bmj.com/site/about/guidelines.xhtml

Lycett et al. Trials 2013, **14**:182
http://www.trialsjournal.com/content/14/1/182

TRIALS

STUDY PROTOCOL Open Access

Slimming World in Stop Smoking Services (SWISSS): study protocol for a randomized controlled trial

Deborah Lycett[1*], Paul Aveyard[2], Andrew Farmer[2], Amanda Lewis[2] and Marcus Munafò[3]

Abstract

Background: Quitting smokers gain weight. This deters some from trying to stop smoking and may explain the increased incidence of type 2 diabetes after cessation. Dieting when stopping smoking may be counterproductive. Hunger increases cravings for smoking and tackling two behaviours together may undermine quitting success.
A meta-analysis of randomized controlled trials (RCTs) showed individualized dietary support may prevent weight gain, although there is insufficient evidence whether it undermines smoking cessation. Commercial weight management providers (CWMPs), such as Slimming World, provide individualized dietary support for National Health Service (NHS) patients; however, there is no evidence that they can prevent cessation-related weight gain. Our objective is to determine whether attending Slimming World from quit date, through referral from NHS Stop Smoking Services, is more effective than usual care at preventing cessation-related weight gain.

Methods: This RCT will examine the effectiveness of usual cessation support plus referral to Slimming World compared to usual cessation support alone. Healthy weight, overweight and obese adult smokers attending Stop Smoking Services will be included. The primary outcome is weight change in quitters 12 weeks post-randomization. Multivariable linear regression analysis will compare weight change between trial arms and adjust for known predictors of cessation-related weight gain.
We will recruit 320 participants, with 160 participants in each arm. An alpha error rate of 5% and 90% power will detect a 2 kg (SD = 2.5) difference in weight gain at 12 weeks, assuming 20% remain abstinent by then.

Discussion: This trial will establish whether referral to the 12-week Slimming World programme plus usual care is an effective intervention to prevent cessation-related weight gain. If so, we will seek to establish whether weight control comes at the expense of a successful quit attempt in a further non-inferiority trial.
Positive results from both these trials would provide a potential solution to cessation-related weight gain, which could be rolled out across England within Stop Smoking Services to better meet the needs of 0.75 million smokers stopping with NHS support every year.

Trial registration: Current Controlled Trials ISRCTN65705512

Keywords: Weight gain, Smoking cessation, Prevention, Obesity, Commercial weight management provider

* Correspondence: deborah.lycett@coventry.ac.uk
[1]Department of Health Professions, Faculty of Health and Life Sciences RC131, Coventry University, Priory Street, Coventry CV1 5FB, UK
Full list of author information is available at the end of the article

BioMed Central

© 2013 Lycett et al.; licensee BioMed Central Ltd. This is an Open Access article distributed under the terms of the Creative Commons Attribution License (http://creativecommons.org/licenses/by/2.0), which permits unrestricted use, distribution, and reproduction in any medium, provided the original work is properly cited.

For peer review only

Lycett *et al. Trials* 2013, **14**:182
http://www.trialsjournal.com/content/14/1/182

Background

Weight gain is a well known consequence of smoking cessation [1,2]. It may deter smokers from attempting to quit [3] and offsets some advantages of giving up smoking. Smoking cessation-related weight gain partly explains the finding that the incidence of type 2 diabetes is increased by up to 73% in the years after cessation [4-6] and there is a 30% increased risk of hypertension [7] compared to continuing smoking. Quitters who gain weight have less improvement in lung function than those who avoid weight gain [8].

An ideal intervention to prevent weight gain would begin at the time of quitting, because weight gain is most rapid initially and then the rate of gain slows [9,10]. Qualitative findings suggest that, in the overweight population, at least, waiting until a quit attempt is established before addressing weight is unacceptable [11].

However, preventing weight gain early in cessation is controversial. The main reason quit attempts fail is due to quitters succumbing to their cravings to smoke. Evidence suggests that hunger increases urges to smoke [12,13] and people who gain most weight are more likely to succeed in quitting smoking [14]. This suggests that avoiding hunger and ameliorating cigarette cravings with food may enhance smoking abstinence and this has become standard advice on how to quit, with healthy food choices often advocated.

We investigated the effectiveness of different interventions to prevent weight gain during cessation in a recent Cochrane review update [15]. General dietary education to reduce energy intake through eating a low fat, healthy diet and increasing exercise did not prevent weight gain compared to standard smoking cessation behavioral support. Furthermore, there was a statistically significant reduction in abstinence at 12 months (Table 1). However, an individually tailored plan to reduce energy intake and increase exercise, with regular monitoring and adaptation of individual goals, reduced weight gain at 6 and 12 months without a statistically significant reduction in abstinence rates (Table 1), although the sample size is too small to be conclusive.

Commercial weight management providers (CWMPs) offer this type of individually tailored dietary and exercise plan. They have already been shown to be an effective way for people to achieve successful weight loss through referral from NHS primary care [16]. Therefore referral to a CWMP on prescription from the National Health Service (NHS) Stop Smoking Services may be an effective way to prevent smoking-related weight gain without a detrimental effect on quitting success. The first step to investigating this is to run a trial to assess whether this is an effective way to prevent weight gain during smoking cessation.

We will use the results of such a trial to inform a larger trial powered to investigate non-inferiority on smoking cessation rates. This will assess whether weight control comes at the expense of a successful quit attempt. This trial would require over 1,100 participants and is premature without first showing that the intervention can reduce weight gain, which is what we propose here. We will seek further funding for the larger trial to investigate effects on abstinence if the intervention reduces weight gain.

Methods

Design

This is an open label, phase II, randomized controlled trial (RCT) to compare standard stop smoking behavioral support with an intervention that will, in addition to providing standard stop smoking support, include personalized weight management support, provided by Slimming World.

This is an open label trial, so blinding patients and smoking cessation advisors to allocation to intervention or control is impossible. The primary outcome is objectively measured weight and therefore the scope for bias is limited. Stop smoking advisors, who will weigh participants, will not provide the weight control support and are unlikely to have a vested interest in interpreting weight change favorably. In addition, they will be required to follow a protocol that specifies outdoor coats and shoes only are removed before weighing.

Setting

We propose recruiting participants from NHS commissioned Stop Smoking Services within Bristol and North Somerset in the first instance. Participating services located within pharmacies, general practices and community centers will display an A3 poster advertising the trial.

Participants

Inclusion criteria

- Daily smokers with expired CO >10 ppm.
- Aged 18 years or over.

Table 1 Effects of dietary interventions on weight change and abstinence during smoking cessation

Intervention compared to standard smoking cessation	Mean difference in weight change (kg (95% CI))		Abstinence (RR (95% CI))	
	End of treatment	At 12 months	End of treatment	At 12 months
General lifestyle and calorie reducing dietary advice	−0.04 (−0.57, 0.50)	−0.21 (−2.28, 1.86)	0.90 (0.76, 1.06)	0.66 (0.48, 0.90)
Individually tailored dietary and lifestyle advice	−1.11 (−1.93, −0.29)	−2.58 (−5.11, −0.05)	1.11 (0.84, 1.46)	0.74 (0.39, 1.43)

CI, confidence interval; RR, risk ratio.

For peer review only - http://bmjopen.bmj.com/site/about/guidelines.xhtml

- Body mass index (BMI) greater or equal to 23 kg/m^2. We included participants with an ideal BMI, as well as those who are overweight in the trial. This is because we are not only seeking to help people who are overweight to manage their weight, but also to enable those who are a healthy weight to prevent the weight gain that is associated with smoking cessation. Without engaging in weight management strategies to counteract the physiological and behavioral responses to smoking cessation, 85% of people who quit smoking will gain weight, regardless of their starting BMI [17].
- Willing to be randomized to either the control or intervention arm, and willing and able to comply with the intervention and all study procedures.
- Able to understand and consent to study procedures, assessed at first appointment with the stop smoking advisor.

Exclusion criteria

- Pregnant smokers.
- BMI <23 kg/m^2. Mortality has been shown to be lowest in people with a 22 >BMI <25 [18], therefore preventing weight gain in those with lower BMIs may not lead to health gain.
- Any medical condition in which weight loss would be contraindicated, for example current course of chemotherapy.
- Currently losing weight, intentionally or unintentionally.
- Participating in any other intervention trial.

Withdrawal criteria

Participant decision to withdraw from treatment and follow-up

Participants may choose to withdraw from treatment and/or follow-up at any time. Failure to attend treatment visits will not be considered as withdrawal from the whole trial, and these participants will still be contacted for follow-up appointments unless they have requested otherwise. We expect that participants who actively request to withdraw from the trial and no longer be contacted will be fewer than 5% of participants, and as such we will not replace these individuals.

Research team decision to withdraw

The intervention is based on the principles of healthy eating and we do not expect any adverse effects. Standard practice within the intervention is to monitor weight and provide feedback, and therefore an unhealthy rate of weight loss is unlikely to occur for long without correction. In the event that a participant develops a condition in which dietary energy restriction is ill-advised, for example embarking on a course of chemotherapy, they will be withdrawn from treatment. Data up to the time of withdrawal from treatment will be used unless the participant requests otherwise. Before beginning the trial, participants will have to consent to notifying the research team of such a change in medical history which is detailed on the consent form. We will ask the participant's general practitioner to notify the research team of such changes in medical history.

Randomization

A randomization sequence will be generated using computer software. Stratified randomization by stop smoking advisor with blocking within each stratum will be used to ensure balance. The blocks will be randomly ordered blocks of 2, 4 and 6. Participants will be randomized 1:1 to usual care or Slimming World with usual care after they have consented to take part in the study. Stop smoking advisors, who are unaware of the randomization sequence, will open sealed, numbered, opaque envelopes in turn, after consent and initial procedures to determine allocation. Online or telephone randomization is not practical in many smoking cessation clinics because they do not have access to computers and operate outside of normal office hours.

Interventions and comparisons

Trial treatment providers

Trained NHS stop smoking advisors will provide participants with standard smoking cessation support, which is intended to be identical in both arms of the trial. Weight control support will be provided by trained weight management counselors employed by Slimming World. Slimming World is a CWMP which follows the National Institute for Health and Care Excellence (NICE) criteria for clinical practice. It is commissioned by the NHS to provide a weight loss service to patients.

Provision of standard NHS stop smoking support

All participants will receive standard NHS stop smoking support. This will be the withdrawal oriented behavioral support provided by NHS Stop Smoking Services, which increases the chance of a successful quit attempt by four-fold [19]. This consists of weekly behavioral support typically for 2 weeks before and until 4 weeks after quit day, focusing on key behavioral change techniques, and nicotine replacement, varenicline or bupropion are prescribed to relieve withdrawal symptoms.

Standard stop smoking treatment consists of six sessions [20]. These are pre-booked when participants contact the Stop Smoking Service. Our trial will be nested within this usual practice as described below and shown in Figure 1.

For peer review only

For peer review only - http://bmjopen.bmj.com/site/about/guidelines.xhtml

Lycett et al. Trials 2013, 14:182
http://www.trialsjournal.com/content/14/1/182

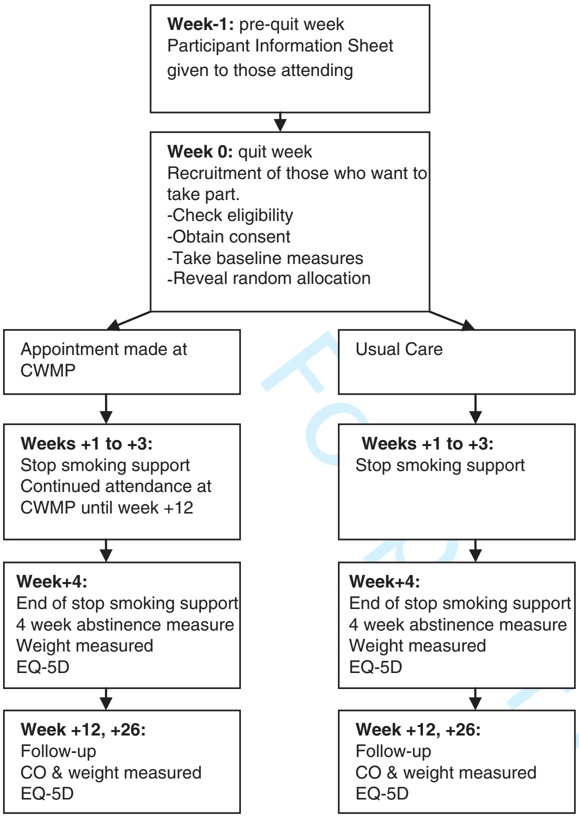

Figure 1 Flow diagram showing flow of participants through trial and main procedures. –, weeks before quitting; 0, quit week; +, weeks after quitting.

Trial procedures

Session 1: pre-quit session (week –1 prior to quit day)

During the first visit, as is usual practice, all smokers will set a quit date for 1 to 2 weeks in advance and their supply of medication to help them quit will be discussed. Smokers will be given the participant information sheet explaining the trial, providing potential participants enough time for consideration between this and the next visit. The information sheet states the opportunity to take part is entirely optional and that choosing not to participate will have no bearing on receiving usual stop smoking support.

Session 2: quit week (week 0)

At the second visit, smokers are usually ready to quit the next day and they will be either already taking varenicline or bupropion, or provided with nicotine replacement therapy at this visit.

Smokers will be asked whether they would like to participate in the trial. Before doing so, the stop smoking advisor will go through the trial information to make sure they have understood what is involved; for example they have to agree to attend Slimming World if randomized to it and be prepared not to go to a CWMP of their own volition if they are not randomized to it.

Those who decline to take part will be invited to give a reason. Those who agree to take part in the trial will provide written evidence of consent and will then be screened for eligibility by the stop smoking advisor. Baseline measures will be taken and they will be randomized into either usual care or Slimming World with usual care. The stop smoking advisor will provide those who are randomized to the intervention with a referral to Slimming World. This is to be taken and used at the local Slimming World group to coincide as near as possible with their quit date.

Baseline measures include a questionnaire asking for each participant’s age, gender, socioeconomic status, ethnicity, religion, daily cigarette consumption, and Fagerström test for nicotine dependence (FTND) score as usually measured by Stop Smoking Services. They will also be asked to complete the EQ-5D (EuroQol, Rotterdam, Netherlands) measure of health status and report whether they suffer from type 2 diabetes mellitus.

Sessions 3 to 5: weeks 1 to 3 post-quit (week +1, +2, +3)

The next three sessions will provide behavioral support for quitting in both the intervention and usual care group. The intervention group will also attend Slimming World.

Session 6: week post-quit (week +4)

This is typically the last visit to the Stop Smoking Service and when 4-week abstinence will be assessed. Weight will be measured and the EQ-5D will be completed by all participants at this visit.

Session 7: 12-week follow-up (week +12)

This corresponds with the end of weight management treatment. Weight and smoking abstinence will be measured. This session is important for determining the sample size needed for the non-inferiority trial which, as is standard in stop smoking trials [21], will require a 6-month follow-up to assess long-term smoking abstinence.

Session 8: 26-week follow-up (+26)

If data from 12-week follow-up shows a difference in mean weight, we will include a 26-week follow-up. The numbers of individuals who will return at this point will be recorded, and weight and smoking abstinence will be measured.

Intervention

CWMPs are available ‘on prescription’ through general practices and provide 12 weeks of free membership of the group-based weight management class. There are three main providers offering services to the NHS: Rosemary Conley, Slimming World and Weight Watchers;

For peer review only - http://bmjopen.bmj.com/site/about/guidelines.xhtml

the cost of a 12-week course to the NHS is about £60. Each of these providers are potentially suitable and their interventions have been described elsewhere [16]. In brief, each provides goal setting for weight, regular monitoring, and a tailored energy restriction using a either a food points system (Weight Watchers), food portion measures (Rosemary Conley), or controlled amounts of high energy foods and personal eating plans (Slimming World).

For this trial, we are using Slimming World, since this provider has been successful at improving health and reducing health risks associated with specific medical conditions through referral schemes within particular patient populations, some of which are designed to prevent excessive weight gain rather than focus on weight loss, for example during pregnancy [22,23]. Such an approach is particularly pertinent to quitting smokers who need to prevent cessation-related weight gain.

In addition to usual care, participants will be given a referral form for Slimming World when they attend their pre-quit visit. The stop smoking advisor will discuss times and locations of local group meetings, and attendance at a convenient group will be agreed. The participant will attend weight management sessions from their quit day (or as near to that date as possible). They will attend Slimming World for 12 weeks receiving support to lose weight or prevent weight gain. The choice of modest weight loss or weight gain prevention will depend upon whether a participant wants to lose weight or not, and whether or not the participant is overweight or obese at baseline.

Typically, Slimming World members lose 0.3 kg/week (or 0.4 kg/week in people who complete 10 of the 12 weeks) [24]. We know that on average 0.2 kg/week is gained with smoking during the first 12 weeks of quitting [10]. Factoring in this expected change in weight resulting from quitting, we would expect a mean weight loss of 0.2 kg/week. For participants who are overweight, a theoretical minimum weight loss goal may therefore be 2.4 kg or 3/4 stone over 12 weeks, although trials to date to prevent smoking cessation weight gain have not achieved as much as this (Table 1). Slimming World consultants typically agree weight loss targets with their members at the first appointment, so appropriate targets will be negotiated on an individual level within realistic margins. Slimming World consultants then work with their members to achieve this.

For participants whose starting BMI is 23 kg/m^2, for example a 60 kg woman with a height of 1.62 m, the weight loss of 2.4 kg described above would result in a BMI of no less than 22 kg/m^2, which is in the optimum range for longevity; this is why we have chosen a BMI less than 23 kg/m^2 for our exclusion criteria.

Most people attending Slimming World do so with weight loss as their goal. For participants in this study who are only seeking to prevent further weight gain, they will be reassured that weight maintenance (+/−0.5 kg over 12 weeks (see section on sample size)) is an equally worthwhile target during smoking cessation and the participant information sheets for the trial will clearly explain this.

Control

Participants are encouraged to quit smoking first, before tackling weight. Stop smoking advisors can advise satiating hunger with healthy foods, but they will not provide detailed or personalized advice on weight and will not set weight targets.

Outcome measures

Primary outcome measure

Change in weight from baseline to 12 weeks post-quit day in abstinent smokers. Abstinence is prolonged abstinence, defined according to the Russell Standard as self-report of no more than five cigarettes since 2 weeks following quit day and expired CO less than 10 ppm [21]. Portable validated scales will be provided to participating Stop Smoking Services. Training in measurement procedures and the trial protocol will be provided for all advisors. The same scales will be used at each clinic at all time points. All measures will be recorded on paper case report forms (CRFs) and later entered into a trial database.

Secondary outcome measures

- Weight at 4 weeks post-quit date.
- Weight at 6 months from baseline.
- Abstinence at 4, 12 and 26 weeks. Abstinence will be defined as not smoking more than five cigarettes since 2 weeks after the quit day verified by expired CO <10 ppm at these visits (Russell Standard criteria). Abstinence will be recorded on the CRF and in the trials database.
- Participant acceptability.

Participant acceptability

Participant acceptability will be measured quantitatively by response, attrition rates and a questionnaire as described below:

- Rates of response to verbal invitation to take part by stop smoking advisors, including reasons for declining to participate.
- Rates of attendance at the commercial weight management meetings by participants randomized to this intervention.

For peer review only - http://bmjopen.bmj.com/site/about/guidelines.xhtml

- Rates of drop out from Stop Smoking Services in intervention compared to usual care.
- Rates of attendance at Stop Smoking Services for follow-up at 12 and 26 weeks.
- The above measures of acceptability according to starting BMI; for example are participants who are obese or overweight more accepting of the intervention which refers to Slimming World than those of a healthy weight?
- A questionnaire will be given to participants who decline to take a part, which will ask them to provide their age, gender, ethnicity, weight status, and reason(s) for declining using a series of closed answer responses (for example: 'I do not want to take part in research,' 'I do not think the intervention will work,' 'I do not want to lose weight') and one open 'other' category.
- All participants who drop out of treatment or follow-up will be asked, if they are willing, the reason for discontinuing using a series of closed answer responses (for example: 'it was too hard to quit smoking,' 'it was too hard to lose weight,' 'I did not like the allocation I received') and one open 'other' category.

Feasibility

Feasibility of running the trial within the Stop Smoking Services will be assessed by questionnaire. Participating services will be asked to rate the ease of adhering to the research procedures and describing any difficulties encountered, such as problems with recording data.

Exploratory outcomes measures

- Self-reported incidence of diabetes.
- Change in EQ-5D.
- Cigarette withdrawal symptom scores as measured by the mood and physical symptoms scale (MPSS) [25].
- Associations of change in religious engagement (adapted from CSI-MEMO [26]) and religious coping (religious coping index (RCI) [27]) at baseline, and their change over time with tobacco withdrawal, quitting success and weight maintenance outcomes.

A recent systematic review of observational studies shows less smoking is associated with religion. Also, despite a higher BMI, religious involvement is associated with better physical and psychological outcomes [28]. The use of religion as a means to cope with health problems is a potential mechanism behind this association [27].

Clinical trials comparing weight loss interventions with a religious element to those without have shown increased physical activity [29], improved dietary quality [30] and greater mean weight loss [31,32] in participants receiving the additional religious element. Survey and qualitative data suggest smokers [33] and those who overeat [34] are receptive to incorporating a spiritual element into treatment programmes.

Although this evidence suggests a beneficial association and an openness to addressing spiritual needs in those who need to lose weight and stop smoking, many of these studies have been carried out in the Bible Belt, USA, and their applicability to the UK population needs to be determined.

Statistics and data analysis

Sample size

A meta-analysis of studies shows that smokers who achieve 12 weeks of abstinence gain around 2.5 kg (SD = 2.5) [10]. A Cochrane review to prevent weight gain when people stop smoking [15] showed that at 4 and 52 weeks post-quit there was a difference of 1.1 kg and 2.6 kg, respectively, between usual care and individualized dietary intervention (Table 1). This means that individualized dietary interventions prevented most of the expected weight gain at 4 weeks post-quit and half of it at 1 year post-quit. A realistic estimate is therefore that we may be able to prevent 80% (2 kg) of the expected weight gain at 12 weeks post-quit, that is the intervention group might gain only 0.5 kg on average. This would require about 32 participants per arm, 64 participants in total, with an alpha error rate of 5% and 90% power. Most smokers who attempt abstinence will relapse to smoking, probably lose any weight gained [9,31] and will not be included in the primary outcome assessment. Considering that approximately only 20% will remain abstinent at 12 weeks, we will need to randomize 160 participants in each trial arm and 320 participants overall.

Analysis plan

Primary analysis

Change in weight between intervention and the control arm of the study will be compared using a *t*-test and then adjusted for known predictors of weight gain after cessation, including stop-smoking medication use, using linear regression analysis. Weight will be analysed in long-term abstainers because only long-term abstainers gain more weight over time than the rest of the population.

Secondary analysis

Abstinence will be analysed on an intention-to-treat basis. This phase II trial is underpowered to detect the difference between abstinence rates in the control and intervention arm. We will calculate the proportion of participants abstinent in each trial arm. Analysis of abstinence rates will assume participants who do not

For peer review only - http://bmjopen.bmj.com/site/about/guidelines.xhtml

Lycett et al. Trials 2013, 14:182
http://www.trialsjournal.com/content/14/1/182

report for follow-up have reverted to smoking, which is standard practice. Quantitative measures of acceptability will be presented as descriptive statistics.

Exploratory outcomes

The association of exploratory outcomes with weight control and quitting success will be examined using regression modeling and on an intention-to-treat basis. We will calculate cost of intervention, but will not carry out any specific health economic modeling in this phase II trial, since it would be premature to do so. We need first to establish whether this weight management can prevent weight gain, before assessing the combined effects of the weight gain prevented and the effects on cessation rates in a phase III trial.

Loss to follow-up

In accordance with the Russell Standard, we will conduct an intention-to-treat analysis and assume participants lost to follow-up are smokers. We will attempt to contact participants who do not attend. Reminder phone calls will also be made before 12-week and 6-month follow-up appointments.

Trial closure

The end of the trial will be the date of the last follow-up of the last participant. Six-month follow-up is contingent upon data at 12-week follow-up.

Study procedures

Training

Stop smoking advisors will be trained in the research procedures for this study, in line with Good Clinical Practice (GCP) principles. Training will include how to screen participants, explain randomization, obtain consent and document data. Stop smoking advisors will be given the opportunity to practice via role play and will be observed doing so. In addition, stop smoking advisors will be provided with a 'script' and a prompt sheet for data recording for consultations that are beyond usual care.

In the case of any uncertainty (regarding suitability for inclusion), advisors will be able to contact the research team for clarification.

Data handling, quality assurance, record keeping and retention

Data management will be undertaken according with the standard operating procedures (SOPs) of the Primary Care Clinical Research and Trials Unit (PC-CRTU) at the University of Birmingham, Edgbaston, UK. The PC-CRTU is fully compliant with the Data Protection Act 1998 and the International Conference on Harmonisation Good Clinical Practice (ICH GCP). The trial will be registered with the Data Protection Act website at the University of Birmingham. Participant identifiable data will be shared only within the clinical team on a need-to-know basis to provide clinical care, and to ensure good and appropriate follow-up. Patient identifiable data may also be shared with approved auditors from the research ethics committee (REC), the general practitioner, and NHS Research and Development (R&D). Otherwise, confidentiality will be maintained and no one outside the clinical or trial team will have access to either the case report forms or the database. On completion of the trial, data will be transferred to Modern Records, a secure archiving facility at the University of Birmingham, where they will be held for a minimum of 10 years and then destroyed.

Case report forms (CRFs)

Data will be collected on a paper CRF and later entered into an anonymized database for analysis at the University of Birmingham. The database will contain agreed data codes, for example for missing data.

Risk assessment

Healthy dietary and lifestyle advice will be individually tailored to create a mild energy deficit and gentle increase in activity. This advice will be given by appropriately trained Slimming World consultants. It is usual practice and considered very safe. Participants will be given the contact details of the trial team to report and discuss any concerns.

Monitoring at study site

The principal investigator will assess adherence to the trial protocol through observation in clinics, for example to check advisors are not coercing smokers to take part, check the implications of randomization are adequately explained and to check the correct randomization procedure is followed. Record keeping will also be monitored, for example recording of weights and exhaled CO readings with random checks of the CRFs. Exhaled CO readings and participant existence will be verified by comparison to the source documents, that is stop smoking advisors records. Any deviations will be recorded, discussed with the stop smoking advisor concerned and corrected either immediately or at following clinics. The trial is potentially subject to audit by the appropriate regulatory authorities and monitoring by the lead Comprehensive Clinical Research Network, and therefore participants will be asked to provide consent to allow their records to be viewed by these authorities.

Ethics approval

The study co-ordination centre has obtained approval from the Cornwall and Plymouth Research Ethics Committee. The trial will be conducted in accordance with the recommendations for physicians involved in

For peer review only - http://bmjopen.bmj.com/site/about/guidelines.xhtml

research on human subjects adopted by the 18th World Medical Assembly, Helsinki, 1964, and later revisions.

Participant consent

The process for obtaining participant informed consent will be in accordance with GCP. The stop smoking advisor and the participant shall both sign and date the consent form before any trial procedures begin. A copy of the signed form will be kept by the participant and the original will be retained at the trial site. A second copy will be forwarded to the general practitioner to file in the participant's medical notes. The participant's decision to take part in the trial is entirely voluntary. It will be explained to potential participants that they can withdraw consent at any time without penalty or affecting the quality or quantity of their future medical care.

Participants will be informed of any relevant information that becomes available that affects their participation in the study. Revised consent forms will be used if applicable and amended forms will be submitted to the main REC for favourable opinion prior to use. Revised informed consent forms will be signed by the parties specified above.

Confidentiality

Participants' identification data will be required for the registration process. The study co-ordination centre will preserve the confidentiality of participants taking part in the trial and is registered under the Data Protection Act 1998. Consent will be sought from patients for members of the study team, regulatory authorities and the relevant health provider to access patient medical records.

Indemnity

The University of Birmingham holds public liability (negligent harm) and clinical trial (non-negligent harm) insurance policies, which apply to this trial.

Sponsor

The University of Birmingham will act as the sponsor for this trial.

Funding

Pump priming funds from the UK Centre for Tobacco Control Studies (UKCTCS) are funding this trial and the National Institute for Health Research School for Primary Care Research (NIHR-SPCR) paid the fellowship of the chief investigator while at the University of Birmingham.

Audits

The trial may be subject to inspection and audit by The University of Birmingham, under their remit as sponsor, the study co-ordination centre and other relevant bodies, for example National Research Ethics Service.

Participant payments

Participants will receive payment for travel and inconvenience at the following visits:

- 12-week follow-up: £5 voucher.
- 6-month follow-up: £5 voucher.
- Payments to NHS Stop Smoking Service.

The NHS Stop Smoking Service will be reimbursed for their time as appropriate from service support funding, for example to obtain participant consent.

Treatment costs

Excess treatment costs of a 12-week membership at Slimming World will be provided to the trial free of charge in the form of vouchers by Slimming World.

Discussion

This trial will establish whether referral to the 12-week Slimming World programme plus usual care is an effective intervention to prevent cessation-related weight gain. If so, we will seek to establish whether weight control comes at the expense of a successful quit attempt in a further non-inferiority trial.

Positive results from both these trials would provide a potential solution to cessation-related weight gain, which could be rolled out across England within Stop Smoking Services to better meet the needs of 0.75 million smokers stopping with NHS support every year.

Trial status

Participant recruitment began in October 2012.

Abbreviations

BMI: Body mass index; CI: Confidence interval; CRF: Case report form; CWMP: Commercial weight management providers; FTND: Fagerström test for nicotine dependence; GCP: Good Clinical Practice; ICH GCP: International Conference on Harmonisation Good Clinical Practice; LES: Local enhanced services; MPSS: Mood and physical symptoms scale; NIHR: National Institute for Health Research; NHS: National Health Service; NICE: National Institute for Health and Care Excellence; PC-CRTU: Primary Care Clinical Research and Trials Unit; R&D: Research and Development; RCI: Religious coping index; RCT: Randomized controlled trial; REC: Research ethics committee; SOP: Standard operating procedure; SPCR: School for Primary Care Research; UKCTCS: UK Centre for Tobacco Control Studies.

Competing interests

DL has received hospitability from manufacturers of smoking cessation products, Pfizer Ltd, Tadworth, UK. DL's institution has received smoking cessation products for use in a clinical trial from Johnson & Johnson Ltd, New Brunswick, NJ, USA. DL has received Slimming World membership vouchers for use in this trial. DL has received expenses and consultancy fees from the NHS and Universities for teaching about cessation-related weight gain. DL has received grant funding from UKCTCS and the NIHR-SPCR for research relating to cessation-related weight gain. PA has undertaken consultancy and research for manufacturers of smoking cessation medications in the past 5 years. AF receives funding from NIHR Oxford Biomedical Research Centre and his institution has received funding in respect of consulting work undertaken with Pfizer Ltd, manufacturers of varenicline, a stop smoking drug. AL has received hospitality from Weight Watchers. In the last 5 years, MM has received grant funding from Pfizer Ltd and varenicline, for research purposes.

For peer review only

Authors' contributions

DL designed the study and drafted the manuscript. AP, AF, AL and MM contributed to study design and manuscript revision. All authors read and approved the final manuscript.

Acknowledgements

We gratefully acknowledge the funding of the salary for DL, provided by a fellowship from the NIHR-SPCR at the University of Birmingham. Additional funds to set up the trial and buy equipment were provided by the NIHR-SPCR and UKCTCS. The UKCTCS is one of five UK Public Health Research Centres of Excellence. We also gratefully acknowledge funding from the British Heart Foundation, Cancer Research UK, the Economic and Social Research Council, Medical Research Council and the Department of Health, under the auspices of the UK Clinical Research Collaboration. AL's salary is paid by NIHR-SPCR, and the salaries of PA, AF and MM are paid by their respective institutions. We thank Celia Hall and her colleagues at EMO Ltd for their help facilitating this research project. We thank Carolyn Pallister and her colleagues at Slimming World for their cooperation and provision of the intervention free of charge to trial participants. We thank Bristol and North Somerset Stop Smoking Services for their participation.

Author details

[1]Department of Health Professions, Faculty of Health and Life Sciences RC131, Coventry University, Priory Street, Coventry CV1 5FB, UK. [2]Department of Primary Health Care Sciences, University of Oxford, Radcliffe Observatory Quarter, Woodstock Road, Oxford OX2 6GG, UK. [3]School of Experimental Psychology, University of Bristol, 12a Priory Road, Clifton, Bristol BS8 1TU, UK.

Received: 21 November 2012 Accepted: 28 May 2013
Published: 19 June 2013

References

1. Klesges RC, Winders SE, Meyers AW, Eck LH, Ward KD, Hultquist CM, Ray JW, Shadish WR: **How much weight gain occurs following smoking cessation? A comparison of weight gain using both continuous and point prevalence abstinence.** *J Consult Clin Psychol* 1997, **65**(2):286–291.
2. Klesges RC, Winders SE, Meyers AW, Eck LH, Ward KD, Hultquist CM, Ray JW, Shadish WR: **Smoking, body weight and their effects on smoking behavior: a comprehensive review of the literature.** *Psychol Bull* 1989, **106**:204–230.
3. Brouwer RJN, Pomerleau CS: **"Prequit attrition" among weight-concerned women smokers.** *Eat Behav* 2000, **1**(2):145–151.
4. Wannamethee SG, Shaper AG, Perry IJ: **Smoking as a modifiable risk factor for type 2 diabetes in middle-aged men.** *Diabetes Care* 2001, **24**(9):1590–1595.
5. Davey-Smith G, Bracha Y, Svendsen KH, Neaton JD, Haffner SM, Kuller LH, Multiple Risk Factor Intervention Trial Research Group: **Incidence of type 2 diabetes in the randomized multiple risk factor intervention trial.** *Ann Intern Med* 2005, **142**(5):313–322.
6. Yeh HC, Duncan BB, Schmidt MI, Wang NY, Brancati FL: **Smoking, smoking cessation, and risk for type 2 diabetes mellitus.** *Ann Intern Med* 2010, **152**(1):10–17.
7. Gerace TA, Hollis JF, Ockene JK, Svendsen KH: **Smoking cessation and change in diastolic blood pressure, body weight, and plasma lipids. MRFIT Research Group.** *Prev Med* 1991, **20**(5):602–620.
8. Chinn S, Jarvis D, Melotti R: **Smoking cessation, lung function, and weight gain: a follow-up study.** *Lancet* 2005, **365**(9471):1629–1635.
9. O'Hara P, Connett JE, Lee WW, Nides M, Murray R, Wise R: **Early and late weight gain following smoking cessation in the Lung Health Study.** *Am J Epidemiol* 1998, **148**(9):821–830.
10. Aubin HJ, Farley A, Lycett D, Lahmek P, Aveyard P: **Weight gain in smokers after quitting cigarettes: meta-analysis.** *BMJ* 2012, **345**:e4439.
11. Lycett D, Aveyard P: *Poster 58: Acceptability of dietary interventions to prevent weight gain during smoking cessation*, UK Society for Behavioural Medicine, 7th Annual Scientific Meeting: Conference Proceedings. Newcastle upon Tyne: UK Society for Behavioural Medicine; 2011:81 [http://uksbm.org.uk/wp-content/uploads/2012/03/Combined-final-conference-proceedings-UKSBM-ASM-2011.pdf]
12. Cheskin LJ, Hess JM, Henningfield J, Gorelick DA: **Calorie restriction increases cigarette use in adult smokers.** *Psychopharmacology (Berl)* 2005, **179**(2):430–436.
13. Leeman RF, O'Malley SS, White MA, McKee SA: **Nicotine and food deprivation decrease the ability to resist smoking.** *Psychopharmacology (Berl)* 2010, **212**(1):25–32.
14. Hall SM, Tunstall CD, Vila KL, Duffy J: **Weight gain prevention and smoking cessation: cautionary findings.** *Am J Public Health* 1992, **82**(6):799–803.
15. Farley AC, Hajek P, Lycett D, Aveyard P: **Interventions for preventing weight gain after smoking cessation.** *Cochrane Database Syst Rev* 2012, **1**, CD006219.
16. Jolly K, Lewis A, Beach J, Denley J, Adab P, Deeks JJ, Daley A, Aveyard P: **Comparison of range of commercial or primary care led weight reduction programmes with minimal intervention control for weight loss in obesity: Lighten Up randomised controlled trial.** *BMJ* 2011, **343**:d7243.
17. Lycett D, Munafò M, Johnstone E, Murphy M, Aveyard P: **Associations between weight change over 8 years and baseline body mass index in a cohort of continuing and quitting smokers.** *Addiction* 2011, **106**:188–196.
18. Prospective Studies Collaboration, Whitlock G, Lewington S, Sherliker P, Clarke R, Emberson J, Halsey J, Qizilbash N, Collins R, Peto R, Lewington S, MacMahon S, Aromaa A, Baigent C, Carstensen J, Chen Z, Clarke R, Collins R, Duffy S, Kromhout D, Neaton J, Qizilbash N, Rodgers A, Tominaga S, Törnberg S, Tunstall-Pedoe H, Chambless L, De Backer G, De Bacquer D, Kornitzer M, *et al*: **Body-mass index and cause-specific mortality in 900 000 adults: collaborative analyses of 57 prospective studies.** *Lancet* 2009, **373**:1083–1096.
19. West R: *How to best motivate and help smokers to stop.* London: University College London; 2010 [www.rjwest.co.uk/slides.php]
20. McEwen A: *Standard Treatment Programme: One-to-one smoking cessation support.* 2nd edition. London: NHS Centre for Smoking Cessation and Training; 2011.
21. West R, Hajek P, Stead L, Stapleton J: **Outcome criteria in smoking cessation trials: proposal for a common standard.** *Addiction* 2005, **100**(3):299–303.
22. Lavin J, Bye C, Avery A, Stubbs J: *Development of Slimming World on referral for specific patient groups.* Presented at Obesity and Health Conference; 2008.
23. Pallister C, Allan J, Lavin J, Avery A: **Changes in well-being, diet and activity habits of pregnant women attending a commercial weight management organisation.** *J Hum Nutr Diet* 2010, **23**(4):459.
24. Stubbs J, Pallister C, Whybrow S, Avery A, Lavin J: **Weight outcomes audit for 34,271 adults referred to a primary care/ commercial weight management partnership scheme.** *Obes Facts* 2011, **4**(2):113–120.
25. West R, Hajek P: **Evaluation of the mood and physical symptoms scale (MPSS) to assess cigarette withdrawal.** *Psychopharmacol* 2004, **177**:195–199.
26. Puchalski C, Romer AL: **Taking a spiritual history allows clinicians to understand patients more fully.** *J Palliat Med* 2000, **3**:129–137.
27. Koenig HG, Cohen HJ, Blazer DG, Pieper C, Meador KG, Shelp F, Goli V, DiPasquale B: **Religious coping and depression among elderly, hospitalized medically ill men.** *Am J Psychiatry* 1992, **149**(12):1693–1700.
28. Koenig H, King D, Carson VB: *Handbook of Religion and Health.* 2nd edition. New York: Oxford University Press; 2012.
29. Duro OK, Sarkisin CA, Leng M, Mangione CM: **Sisters in motion: a randomised controlled trial of a faith based physical activity intervention.** *J Am Geriatr Soc* 2010, **58**(10):1863–1869.
30. Djuric Z, Mirasolo J, Kimbrough L, Brown DR, Heilbrun LK, Canar L, Venkatranamamoorthy R, Simon MS: **A pilot trial of spirituality counselling for weight loss maintenance in African American breast cancer survivors.** *J Natl Med Assoc* 2009, **101**(6):552–564.
31. Kim KH, Linnan L, Campbell MK, Brooks C, Koenig HG, Wiesen C: **The WORD (Wholeness, oneness, righteousness, deliverance): a faith-based weight-loss programme utilising a community-based participatory research approach.** *Health Educ Behav* 2008, **35**(5):634–650.
32. Parker VG, Coles C, Logan BN, Davis L: **The LIFE project: a community based weight loss intervention program for rural African-American women.** *Fam Community Health* 2010, **33**(2):133–143.
33. Gonzales D, Redtomahawk D, Pizacani B, Bjornson WG, Spradley J, Allen E, Lees P: **Support for spirituality in smoking cessation: results form a pilot survey.** *Nicotine Tob Res* 2007, **9**(2):299–303.
34. Natti R, Galit L: **Eating disorders and recovery: lessons from overeaters anonymous.** *Clin Soc Work J* 2003, **31**(2):155–171.

doi:10.1186/1745-6215-14-182
Cite this article as: Lycett *et al.*: Slimming World in Stop Smoking Services (SWISSS): study protocol for a randomized controlled trial. *Trials* 2013 **14**:182.

For peer review only

BMJ Open

Referral to Slimming World in UK Stop Smoking Services (SWISSS) versus Stop Smoking Support Alone on Body Weight in Quitters: Results of a Randomized Controlled Trial

Journal:	*BMJ Open*
Manuscript ID	bmjopen-2019-032271.R1
Article Type:	Original research
Date Submitted by the Author:	24-Oct-2019
Complete List of Authors:	Lycett, Deborah; Coventry University<br>Aveyard, Paul; University of Oxford, Primary Care Health Sciences<br>Farmer, Andrew; University of Oxford, Department of Primary Care Health Sciences<br>Lewis, Amanda; University of Bristol, Population Health Sciences Bristol Medical School<br>Munafo, Marcus; University of Bristol, Experimental Psychology
**Primary Subject Heading**:	Public health
Secondary Subject Heading:	Smoking and tobacco
Keywords:	weight, smoking, obesity

SCHOLARONE™
Manuscripts

Referral to Slimming World in UK Stop Smoking Services (SWISSS) versus Stop Smoking Support Alone on Body Weight in Quitters: Results of a Randomized Controlled Trial

Deborah Lycett PhD[1*]

[*] Corresponding author

Email: deborah.lycett@coventry.ac.uk

Paul Aveyard PhD[2]

Email: paul.aveyard@phc.ox.ac.uk

Andrew Farmer DM[2]

Email: andrew.farmer@phc.ox.ac.uk

Amanda Lewis PhD[3]

Email: amanda.lewis@bristol.ac.uk

Marcus R. Munafò PhD[4]

Email: marcus.munafo@bristol.ac.uk

[1] Faculty of Health & Life Sciences, Coventry University, Priory Street, Coventry CV1 5FB, UK. [2] Nuffield Department of Primary Health Care Sciences, University of Oxford, Radcliffe Observatory Quarter, Woodstock Road, Oxford OX2 6GG, UK. [3]Population Health Sciences Bristol Medical School, University of Bristol, Canynge Hall (Room G.08a), 39 Whatley Road

For peer review only

Bristol, BS8 2PS. [4]MRC Integrative Epidemiology Unit, UK Centre for Tobacco and Alcohol Studies, School of Experimental Psychology, University of Bristol, 12a Priory Road, Clifton, Bristol BS8 1TU, UK.

ABSTRACT

Introduction

Most people who stop smoking gain weight. Dietary modification may seem an obvious solution, but food restriction may increase cigarette craving and smoking relapse.

Trial Design

An unblinded parallel randomised controlled trial

Methods

Participants were adult smokers with a body mass index (BMI) greater or equal to 23 kg/m^2.

Setting was NHS commissioned Stop Smoking Services,

Interventions were referral to a commercial weight management programme, plus stop smoking support (treatment group), compared to stop smoking support alone (control group).

Objective was to compare weight change between interventions in smoking abstainers and not abstinent rates in all.

Primary outcome was change in weight (kg) at 12 weeks.

Randomisation sequence was computer generated and concealed until allocation.

For peer review only

Results

Seventy-six participants were recruited, 37 were randomized to the treatment group and 39 to the control group.

Change in weight was analysed in long-term abstainers (13 treatment, 14 control) only because the aim was to prevent weight gain associated with smoking cessation.

Abstinence was analysed on an intention-to-treat basis (37 treatment, 39 control).

At 12 weeks weight gain was less in the treatment than the control group with an adjusted mean difference of -2.3kg 95% CI [-4.4, -0.1]. Craving scores were lower (MPSS-C -1.6 [-2.7, -0.5]) and quit rates were higher in the treatment than the control group (32% vs 21%), although the trial was not powered to superiority in cravings and quit rates..

No adverse events or side effects were reported.

Conclusion

In people who are obese and want to quit smoking, these data provide modest encouragement that providing weight management at the time of quitting may be helpful. Those who are not obese, but who are informed about potential weight gain during their quit attempt, were uninterested in a weight management programme.

TRIAL REGISTRATION

Current Controlled Trials ISRCTN65705512

http://www.controlled-trials.com/ISRCTN65705512

For peer review only

ARTICLE SUMMARY

Strengths and limitations of this study

- The first study investigating referral to a commercial weight loss programme during smoking cessation.
- Computer generated randomisation with allocation concealment.
- Weight measured and abstinence verified by exhaled CO.

- Small study with poor recruitment but reverse power calculation showed greater than 80% power to detect change in primary outcome of weight.

INTRODUCTION

Weight gain is a well-known consequence of smoking cessation [1, 2]. A meta-analysis of weight data, from randomised controlled trials, at one year post quit date showed that in untreated quitters there is a mean increase of 4-5kg. However, variation in weight change is large, with about 16% of quitters losing weight and 13% gaining more than 10kg [3].Nonetheless with the majority of people gaining weight smokers may be deterred from attempting to quit and this weight gain may offset some advantages of giving up smoking. Smoking cessation-related weight gain partly explains the finding that the incidence of type 2 diabetes is increased by up to 73% in the years after cessation [4-6], there is a 30% increased risk of hypertension [7], and a deterioration in glycaemic control in those with diabetes [8] who quit compared to those who continue to smoke. Despite these concerns weight gain in quitters does not negate the risk reduction of cardiovascular morbidity and mortality in those who successfully quit smoking [9]. There is some evidence to suggest the benefit may be

For peer review only

mitigated [10] but the number of incident cases in higher weight gain categories are too small to draw conclusions. Nonetheless, many people find this weight gain unacceptable and the greatest danger is it may deter individuals from quitting [11]. Yet, restricting food intake may limit successful quitting as hunger increases urges to smoke [12, 13] and people who gain most weight quit more successfully [14].

An individually tailored plan to reduce energy intake and increase exercise, with regular monitoring and adaptation of individual goals, appears to be the most promising way to prevent cessation related weight gain with the least risk of impeding a quit attempt [15]. Commercial weight management providers (CWMPs) offer this type of support and are an effective way for many to lose weight through referral from National Health Service (NHS) primary care [16]. Therefore, referral to a CWMP on prescription from the NHS Stop Smoking Services may be an effective way to prevent smoking-related weight gain without a detrimental effect on quitting success.

This study compares referral to a CWMP as part of stop smoking support with stop smoking support alone on changes in body weight in quitting smokers.

METHODS

Design

This was a parallel group, individually randomized controlled trial (RCT) to compare standard stop smoking behavioural support with an intervention that, in addition to providing standard stop smoking support, included personalised weight management support, provided by Slimming World.

For peer review only

This was an open label trial, as blinding patients and smoking cessation advisors to allocation to intervention or control was impossible. The primary outcome was objectively measured weight and therefore the scope for bias is limited. Stop smoking advisors, who weighed participants, did not provide the weight control support and were unlikely to have a vested interest in interpreting weight change favourably. Full details of the methods for this trial can be found in the protocol paper [17] but are summarized below.

Setting

NHS commissioned Stop Smoking Services were first recruited from Bristol and North Somerset Primary Care Trusts (PCTs). Stop smoking services were invited to take part by letter and a follow-up phone call 2 weeks later. Clinics were only eligible to participate if they had 2 new service users coming through their doors each week. Due to poor recruitment we invited more clinics (n=30) to take part in the study by widening our area further across the West Midlands and South-West England. Additionally we extended the planned duration of recruitment from 10 to 17 months, which was as far as our budgetary restraints would allow.

All individuals attending the stop smoking services were invited to take part in the trial at their first visit when they set their quit date. At the second visit those wanting to participate in the trial gave their consent and were randomised to the control or intervention group. Those randomised to the intervention were given a referral to Slimming World, a location and a start date as near to their quit date. Those who declined to take part were invited to provide a reason.

Baseline measures were taken after randomization and included age, gender, socioeconomic status, ethnicity, religion, Fagerström Test for Nicotine Dependence (FTND) [18] and all dimensions of EQ-5D (EuroQol, Rotterdam, Netherlands) as a measure of health-related quality of life [19] presented as index values.

For peer review only

Participants

Daily adult smokers with expired CO ≥10 ppm and a body mass index (BMI) greater or equal to 23 kg/m^2 were included. Pregnant smokers, those with a BMI < 23kg/m^2, those currently losing weight, or where weight loss was contraindicated, were excluded.

Participants with an ideal BMI, as well as those who are overweight, were included in the trial. This is because help is needed not only for quitting smokers who are overweight, but also for those of a healthy weight to prevent the weight gain that is associated with smoking cessation. Without engaging in weight management strategies to counteract the physiological and behavioural responses to smoking cessation, 85% of people who quit smoking will gain weight, regardless of their starting BMI [20].

Randomization

A randomization sequence was generated using computer software. Stratified randomisation by stop smoking advisor with blocking within each stratum was used to ensure balance. The blocks were randomly ordered blocks of 2, 4 and 6. Participants were randomised 1:1 to usual care or Slimming World with usual care. Stop smoking advisors were unaware of the randomisation sequence; they opened sealed, numbered, opaque envelopes in turn.

Interventions and comparisons

Trial treatment providers

Trained NHS stop smoking advisors provided participants with standard smoking cessation support in both arms of the trial. This was withdrawal oriented behavioural support focusing

For peer review only

on key behavioural change techniques, and a prescription of nicotine replacement or varenicline to relieve withdrawal symptoms. Support was on a weekly basis beginning two weeks before and until four weeks after quit day. Weight control support was provided by trained weight management counsellors employed by Slimming World. Slimming World is a CWMP which follows the National Institute for Health and Care Excellence (NICE) criteria for clinical practice. It is commissioned by the NHS to provide a weight loss service to patients. Slimming World was the CWMP of choice as it works with populations to prevent excessive weight gain rather than solely focusing on weight loss, this was pertinent to preventing cessation related weight gain.

Intervention

In addition to usual care, participants attended Slimming World for 12 weeks receiving support to lose weight or prevent weight gain. Slimming World consultants typically agree weight management targets with their members at the first appointment and then work with their members to achieve this through regular weight monitoring, controlled amounts of high energy foods and personal eating plans.

Control

Participants were encouraged to quit smoking first, before tackling weight. Stop smoking advisors advised satiating hunger with healthy foods, which is standard care, but did not provide detailed or personalized advice regarding weight management.

Outcome measures

Primary outcome measure:

For peer review only

Change in measured weight from baseline to 12 weeks post-quit day in abstinent smokers. Abstinence was prolonged abstinence, defined according to the Russell Standard [21].

Secondary outcome measures:

- Weight at 4 weeks post-quit date.
- Weight at 6 months from baseline.
- Abstinence at 4, 12 and 26 weeks
- Participant acceptability measured quantitatively by response, attrition rates and a decliner's reasons for not participating.
- Feasibility of running the trial within the Stop Smoking Services was measured by an open text questionnaire asking for comment.

Exploratory outcomes measures:

- Change in EQ-5D index values.
- Cigarette withdrawal symptom scores as measured by the mood and physical symptoms scale (MPSS) [22].
- Associations of change in religious engagement (adapted from CSI-MEMO [23]) and religious coping (religious coping index (RCI) [24]) as positive use of individual's spiritual reserve to change weight [25] and smoking behaviour [26] is increasingly evidenced.

For peer review only

Statistics and data analysis

Sample size

Difficulty in recruiting resulted in it being economically unaffordable to continue the trial; therefore, the decision was made to stop the trial early.

Following the full analysis of our outcome data we conducted reverse power calculations. This showed that we had >80% power, at an alpha error rate of 0.05, to detect the observed differences in weight change in our sample at 12 weeks between the treatment and control groups. Given a large observed difference in quit rates between the control and treatment groups we also had 90% power at an alpha error rate of 0.05 to determine non-inferiority, using a non-inferiority limit of 19% [27].

Our original sample size had been conservative assuming we would see 20% weight gain in the treatment group, rather than the weight loss we actually saw. This would have required 32 quitters in each trial arm at 12 weeks; instead, we achieved 8-10 quitters at that time point. To achieve 32 quitters in each trial arm, based on a conservative estimate that only 20% of those recruited would be quit at 12 weeks, we wanted to recruit 160 per trial arm. Instead, we only recruited 76 smokers in total, but the quit rates in the treatment group were higher than expected.

Analysis

The change in weight was analysed in long-term abstainers only because only long-term abstainers gain more weight over time than the general population and because the aim was to prevent weight gain on smoking cessation [20]. Therefore, as this comparison was made in abstainers only, and not those randomised to control or treatment groups, investigation of

For peer review only

baseline differences was conducted between these groups. To identify likely confounding variables selected baseline characteristics of age, weight, BMI, FTND, gender, ethnicity and pharmacotherapy were compared, between quitters at 4, 12 and 26 weeks post quit in the control and treatment groups, using t-tests and chi-squared statistics. Weight change in the intervention and the control arm of the study was presented descriptively using means and SDs and the mean difference and 95% confidence intervals (CI) between the two arms calculated using multiple linear regression with adjustment for baseline differences.

Abstinence was analysed on an intention-to-treat basis, assuming all those not present at follow-up had relapsed [21]. As we recruited fewer people than intended, we could not assume effective randomisation and so investigated whether there were any differences in baseline characteristics between the control and treatment groups. Adjustment for these was then made using logistic multiple regression to obtain an odds ratios for becoming abstinent.

The quality of life (EQ-5D) was presented as index values calculated from interim scoring for the EQ-5D-5L spreadsheet for UK values [28], means and SD of the EQ-5D visual analogue scale (VAS) were also presented following the EQ-5D-5L reporting guide.

The change in exploratory measures were presented with descriptive statistics and analysed using multiple linear regression. We also reported descriptive data about the acceptability of the intervention, supplemented by comments from questionnaires completed by stop smoking service practitioners. We analysed these using manifest content analysis of a broad surface structure reflecting what was literally said [29]. Open comments were broken down into individual codes of meaning then built up into sub themes and themes which summarised the data.

For peer review only

Ethics approval

The study was approved by Cornwall and Plymouth NHS Research Ethics Committee. The trial was conducted in accordance with the recommendations for physicians involved in research on human subjects adopted by the 18th World Medical Assembly, Helsinki, 1964, and later revisions. All participants gave their written informed consent to participate in the study.

Patient and Public Involvement

A patient advisory group were asked to comment on the concept of the study and the perceived acceptability of the proposed intervention, feedback was positive and so study plans progressed.

Participant reimbursement for attending follow-up visits

Participants received £5 payment for travel and inconvenience at the 12 and 26 week follow-up visits.

RESULTS

Potential Participants

Thirty different stop smoking clinics in the West Midlands and South-West of England inclusion criterion of treating at least two new service users attending a clinic each week. Potentially over 4400 participants could have been accessed over our 17 month recruitment period and invited to take part in the trial. Of those who declined to take part 128 individuals provided us with a reason. Eighty-three people agreed to take part and were screened for eligibility, 7 were ineligible (4 were already losing weight through attending a commercial weight loss provider) and 76 individuals were randomised. Of these, 37 were randomized to the treatment group and 12 returned for the 26 week follow-up, while 39 were randomized to

For peer review only

the control group of which 10 returned for the 26 week follow-up. The majority of those not responding to follow-up did so in the first four weeks of the trial and continued to ignore requests thereafter. Eight withdrew from the trial for various reasons, one individual in the control group did so to join Slimming World and two individuals in the intervention group said it was too difficult to quit and diet at the same time, another reported returning to smoking (Figure 1).

Characteristics of 'decliners' and participants

Those who provided a reason for declining to take part (decliners) and those who agreed to participate were of a similar mean age and mostly white British people. Women were more likely than men to enrol in the trial, but the most striking difference related to baseline weight status. Practically everyone who was obese agreed to join the trial, while a minority in the other BMI groups did so (Table 1).

Over half those who declined to take part did so because they were not interested in weight control. (Proportionally more who gave this reason had a healthy weight rather than being overweight (data not shown).) Over 20% of reasons for declining were because people did not have time. 17% said they were not prepared to go to Slimming World, similar numbers said they could not quit smoking and diet at the same time. Medical reasons and reasons related to the research process such as the principle of taking part or being randomised to the control were not commonly reported (Supplementary Figure 1).

Baseline Characteristics of participants in the treatment and control group at baseline

Baseline characteristics of participants were investigated between the control and treatment groups as failure to recruit to target sample size could have resulted in an imbalance. Those in the control group were significantly younger and with a higher BMI than the treatment group

For peer review only

(Table 2). and adjustment was made for this in the analysis of outcomes.

Weight Change During Abstinence

Among abstinent participants, there was a steady mean weight gain over time in the control group, whereas weight reduced at each time point in the treatment group. In those who were quit at 4 week follow-up there was an adjusted mean difference of -1.2kg [-2.5, 0.1] weight change between those in the intervention group and those in the control group. At 12 weeks the difference was greater --2.3kg [-4.4, -0.1] and some of this initial difference in weight loss between the trial arms was maintained at 26 week follow-up (-3.1kg [-6.9, 0.8]). (Table 3). No confounding variables were identified in the quitters between treatment and control groups at week 4 and 12 post-quit (results no shown), age was a potential confounding variable for those who remained quit at 26 weeks and adjustment for age was included in the analysis of difference in weight between the trial arms.

Change in Abstinence (Intention-to-treat analysis)

In the control group 13 of 39 participants (34%) were abstinent at 4 weeks and in the treatment group 14 of 37 (38%) were abstinent. By 12 weeks 8 (21%) participants in the control and 12 (32%) in the treatment group remained abstinent. At 26 week follow-up 5 (13%) participants in the control group remained abstinent and 8 (22%) in the treatment group.

Change in Exploratory Outcomes

Change in the MPSS craving domain (MPSS-C) at all time-points showed a greater reduction in the treatment than the control group, with mean adjusted differences ranging from 0.7 to 1.9 on a scale of 0-10. In the mood domain, MPSS-M, in the longer term (26 week follow-up)

For peer review only

there was a greater reduction in negative affect in the treatment group than the control group but the adjusted mean difference was negligible -1.3 [-3.7, 1.2] on a scale between 7 and 35. The effect on hunger and MPSS-P within and between the trials arms was negligible at all time-points. There was negligible change in religious coping and the Index Values of EQ-5D between the trial arms. The visual analogue scale (VAS) for quality of life improved between 4-6% in both groups across all time points (Table 4).

Practitioners' comments on trial delivery

Three main themes came out of the content analysis of practitioners' comments, these were 'Aspects related to research processes', which included positive aspects such as ease of implementation, however additional time and paperwork particular on the part of the practitioner, and also the perceived burden for the participant, were viewed negatively. Randomisation to the control group disappointed both the participant and the practitioner; practitioners reported viewing Slimming World vouchers as an incentive and this suggested misunderstanding of the nature and value of randomization and the need to promote the control arm as a good alternative. Such attitudes may have fuelled disappointment and subsequent drop out. The second theme was 'An opportunity to address weight issues', this opportunity was predominantly seen as a positive aspect of the trial and practitioners anticipated this would generate great interest from participants. However, practitioners were somewhat surprised that participants did not seem to want to address weight, there was mention that fewer people at the time of this trial were seeking stop smoking services than expected, and high rates of drop out were also problematic. These comments led to the third theme 'Poor participant interest and attendance' which characterised experience of delivering the trial (Supplementary Table 1).

For peer review only

DISCUSSION

Summary of results and consistency with other findings

The aim of the study was to prevent cessation related weight gain – which as discussed in the introduction has both clinical and psychological consequences, the most harmful of which may be considered as relapse to smoking. The trajectory of cessation related weight gain was seen by a consistent weight increase in the control group. In comparison, the treatment group lost weight at each of these time points. Referral to the 12-week Slimming World programme plus usual stop smoking support achieved significantly less weight gain than usual stop smoking support alone. This adjusted difference (-2.3kg[-4.4, -0.1]) was apparent at 12 weeks post-quit and is slightly greater than that seen in other trials comparing personalised weight management to usual care (-1.1kg [-1.9, -0.3]) [14]. This was partially sustained at 26 weeks post-quit (-3.2kg [-6.9, 0.8]).

However, we were unable to recruit to our target sample size and these results lack precision.

Percentage quit was no worse in the treatment than the control group; it was slightly higher in the treatment group but the trial was underpowered to test superiority. MPSS craving scores dropped further in the treatment than the control group (>1point difference on 10point scale at 12 and 26 weeks). Hunger ratings were similar in both groups across all time points.

Comparing our findings with the meta-analyses of personalised weight management versus no weight management at 26 weeks [15], our study achieved a similar quit rate in our treatment group (22%) as in the control groups of other studies (21%). However the quit rates in our

For peer review only

control group was lower than in these other studies (13% vs 19%) which may account for the difference in quit rates we saw here.

Taken together these results suggest that referral to a commercial weight loss provider, such as Slimming World, at the time of a quit attempt can prevent cessation-related weight gain without negatively impacting a quit attempt. However, we cannot draw firm conclusions given our small sample size.

Limitations

The greatest limitation to this study was the difficulty in recruiting which led to the small sample size. Anecdotally stop smoking advisors often cite weight gain is raised as a concern in clinics and were anticipating a high uptake for this trial, however we found that when given the opportunity participation was poor and more than 50% of people who gave a reason for declining reported it was because they were 'uninterested in weight control'.

While we do not know the reasons for everyone who declined, these results suggest that weight gain may not be as much as a barrier to quitting smoking as is commonly believed, particularly in those who are not obese. However, all those who were invited to participate in the trial had already decided to quit smoking and were looking for support in that goal. In addition, people who were already losing weight or attending a commercial weight loss programme were ineligible to take part (9% the 44 screened for eligibility). The Office for National Statistics reports weight gain only deters 5% of people from quitting and only 3% of people cite it as a reason for returning to smoking [30]. In light of this, and in view of the evidence that the cardiovascular benefits of quitting are evident despite weight gain [9, 10], we can reassure quitters who are worried about gaining weight that they need not be unduly concerned. In fact, evidence from a randomised control trial in the US (350 quitters at 6 months) suggests that

For peer review only

counselling quitters to reduce their weight concerns (through CBT telephone counselling compared to standard quit advice) leads to similar rates of quitting and a significant difference in weight change in favour of the counselling group (-0.5kg vs 1.0kg). Such counselling involved identifying and addressing maladaptive thoughts about body shape and weight, discouraged restrictive dieting, educated quitters on the relationship between weight gain and smoking cessation and encouraged an acceptance of moderate weight gain after quitting [31].

The small sample size also means that we cannot assume effective randomisation, therefore we cannot account for any unmeasured of residual confounding which may have otherwise been explained the results we found.

The trial was also limited by a short-term follow-up at 6 months post-quit (3 months post treatment intervention), therefore the long term consequences of the effect on weight gain prevention are unknown. However, meta-analysis data from randomised controlled trials suggests that 17% of weight lost is maintained 5 years after the end of moderate weight management interventions [32] so we might expect a similar trajectory here. This intervention in stop smoking services may therefore have limited impact in the long-term, nonetheless the short term increase in diabetes risk associated with smoking cessation that is partly explained by weight gain [33] may be reduced, however this needs to be investigated in a larger trial, with a longer period of follow-up.

Another limitation is the high rate of loss to follow-up in both the control and treatment arms of the study. The provision of free weight management advice did not deter attrition. High attrition is common in smoking cessation trials and is usually due to participants returning to smoking and as such the standard approach in analysis is to assume this [21]. However, our trial showed higher loss to follow-up compared to other smoking cessation trials [32], this may

For peer review only

have been due to staff time pressures reported by practitioners which may have limited their continued attempts to make contact with those who did not attend.

Additionally, not all data was fully completed at baseline, there were disproportionally more missing values in the treatment than the control arm but we have no reason why this might be the case.

Our trial did show that a few people were interested in preventing weight gain by attending a behavioural weight loss programme, and those who wanted to take part in the trial were more likely to be female and more likely to be obese. This is consistent with other findings that suggest women who are obese are likely to be the most weight concerned [34]. Therefore, the generalisability of our findings are largely limited to these who are already overweight or obese.

For individuals who feel unable to make a quit attempt without concurrently controlling their weight, this trial suggests that referral to a behavioural weight loss programme may be a pragmatic option within stop smoking clinics.

Implications for future trials to prevent cessation related weight gain:

- Stop smoking service practitioners consider a relatively straightforward trial delivered with support to impose undue burdens on their time, limiting their willingness to approach potential participants. This is mostly due to the paperwork concerned with consent and follow-up. In low-intensity low-risk trials, we need to develop a method for achieving this that does not impose the same burdens as trials of much riskier interventions. In the meantime, triallists need to understand this and make fewer demands by redesigning the trials.

For peer review only

- Most people who are stopping smoking are uninterested in active behavioural support to prevent weight gain. However, where people already are overweight to the extent of being obese, most people were prepared to enter the trial and future trials may target this population.
- Practitioners and participants quickly become despondent if not randomised to their choice of intervention, almost always the intervention group. More intensive training for practitioners in how to present trials to create equipoise is likely to be needed. There was a suggestion that quit rates were higher in the intervention than the control group and it may be helpful to explore qualitatively whether unhappiness at weight gain explains this.

Conclusion

Referral to the 12-week Slimming World programme plus usual stop smoking support achieved weight loss compared with weight gain seen with usual smoking cessation support alone throughout the quit attempt as far as 26 weeks. Quit rates, cravings for cigarettes, feelings of hunger and quit rates did not seem to be adversely affected although the recruited sample was too small to confirm this.

Recruitment into a trial of stop smoking services referred into a behavioural weight loss programme was poor. They most common reason for declining to participate was no interest in controlling weight.

ABBREVIATIONS

BMI: body mass index; CI: confidence interval; CRF: case report form; CWMP: commercial weight management providers; FTND: Fagerström Test for Nicotine Dependence; GCP: Good Clinical Practice; ICH GCP: International Conference on Harmonisation Good Clinical

For peer review only

Practice; LES: local enhanced services; MPSS: mood and physical symptoms scale; NIHR: National Institute for Health Research; NHS: National Health Service; NICE: National Institute for Health and Care Excellence; PC-CRTU: Primary Care Clinical Research and Trials Unit; R&D: Research and Development; RCI: religious coping index; RCT: randomized controlled trial; REC: research ethics committee; SOP: standard operating procedure; SPCR: School for Primary Care Research; UKCTCS: UK Centre for Tobacco Control Studies.

COMPETING INTERESTS

DL has received hospitability from manufacturers of smoking cessation products, Pfizer Ltd, Tadworth, UK. DL's institution during the active phase of the trial has received smoking cessation products for use in a clinical trial from Johnson & Johnson Ltd, New Brunswick, NJ, USA. DL has received Slimming World membership vouchers for use in this trial. DL has received expenses and consultancy fees from the NHS and Universities for teaching about cessation-related weight gain. DL has received grant funding from UKCTCS and the NIHR-SPCR for research relating to cessation-related weight gain. PA is an NIHR senior investigator and is funded by NIHR Biomedical Research Centre and the CLAHRC, Oxford. AF is an NIHR Senior Investigator and receives funding from NIHR Oxford Biomedical Research Centre. AL has received hospitality from Weight Watchers. In the last 5 years, MM has received grant funding from Pfizer Ltd and varenicline, for research purposes.

For peer review only

AUTHOR CONTRIBUTIONS

DL, PA, AF, AL and MM contributed to the design of the trial and interpretation of the data. DL conducted the analysis and drafted the manuscript. DL, PA, AF, AL and MM provided critical review on the drafts and are accountable for the work.

FUNDING

This work was supported by funding of the salary for DL, provided by a fellowship from the NIHR-SPCR during the active trial period. Additional funds to set up the trial and buy equipment were provided by the NIHR-SPCR and UKCTCS. The UKCTCS is one of five UK Public Health Research Centres of Excellence. We also gratefully acknowledge funding from the British Heart Foundation, Cancer Research UK, the Economic and Social Research Council, Medical Research Council and the Department of Health, under the auspices of the UK Clinical Research Collaboration. AL's salary was paid by NIHR-SPCR during the trial period, and the salaries of AF, MM and PA were paid by their respective institutions. PA is an NIHR senior investigator and funded by NIHR Oxford Biomedical Research Centre and CLAHRC. All funding is gratefully acknowledged.

For peer review only

ACKNOWLEDGEMENTS

We thank Carolyn Pallister and her colleagues at Slimming World for their cooperation and provision of the intervention free of charge to trial participants. We thank the Stop Smoking Services for their participation.

DATA STATEMENT

Please contact the corresponding author if you would like to access the trial data.

Figure 1: Flow of Participants through trial

Supplementary Figure 1. Reasons for declining to take part in SWISSS (more than 1 choice possible)

REFERENCES

1. Klesges RC, Winders SE, Meyers AW, et al: **How much weight gain occurs following smoking cessation? A comparison of weight gain using both continuous and point prevalence abstinence.** *J Consult Clin Psychol* 1997, **65**(2):286–291.

2. Klesges RC, Winders SE, Meyers AW, et al: **Smoking, body weight and their effects on smoking behavior: a comprehensive review of the literature.** *Psychol Bull* 1989, **106**:204–230.

3. Aubin H-J, Farley A, Lycett D, Lahmek P, Aveyard P. Weight gain in smokers after quitting cigarettes: meta-analysis BMJ 2012; 345 :e4439

4. Wannamethee SG, Shaper AG, Perry IJ: **Smoking as a modifiable risk factor for type 2 diabetes in middle-aged men.** *Diabetes Care* 2001, **24**(9):1590–1595.

5. Davey-Smith G, Bracha Y, Svendsen KH, Neaton JD, Haffner SM, Kuller LH; Multiple Risk Factor Intervention Trial Research Group: **Incidence of type 2 diabetes in the randomized multiple risk factor intervention trial.** *Ann Intern Med* 2005, **142**(5):313–322.

6. Yeh HC, Duncan BB, Schmidt MI, Wang NY, Brancati FL: **Smoking, smoking cessation, and risk for type 2 diabetes mellitus.** *Ann Intern Med* 2010, **152**(1):10–17.

7. Gerace TA, Hollis JF, Ockene JK, Svendsen KH: **Smoking cessation and change in diastolic blood pressure, body weight, and plasma lipids. MRFIT Research Group.** *Prev Med* 1991, **20**(5):602–620.

For peer review only

8. Lycett, D., Nichols, L., Ryan, R., et al. **The association between smoking cessation and glycaemic control in patients with type 2 diabetes: a THIN database cohort study**. *Lancet Diabetes Endocrinol* 2015. *3*(6), pp.423-430.

9. Clair, C., Rigotti, N.A., Porneala, B., et al. **Association of smoking cessation and weight change with cardiovascular disease among adults with and without diabetes.** *JAMA* 2013, 309(10), pp.1014-1021.

10. Luo J, Rossouw J, Margolis KL. **Smoking Cessation, Weight Change, and Coronary Heart Disease Among Postmenopausal Women With and Without Diabetes.** *JAMA*. 2013;310(1):94–96. doi:10.1001/jama.2013.6871

11. Brouwer RJN, Pomerleau CS: **"Prequit attrition" among weight-concerned women smokers.** *Eat Behav* 2000, **1**(2):145–151.

12. Cheskin LJ, Hess JM, Henningfield J, Gorelick DA: **Calorie restriction increases cigarette use in adult smokers.** *Psychopharmacology (Berl)* 2005, **179**(2):430–436.

13. Leeman RF, O'Malley SS, White MA, McKee SA: **Nicotine and food deprivation decrease the ability to resist smoking.** *Psychopharmacology (Berl)* 2010, **212**(1):25–32.

14. Hall SM, Tunstall CD, Vila KL, Duffy J: **Weight gain prevention and smoking cessation: cautionary findings.** *Am J Public Health* 1992, **82**(6):799–803.

15. Farley AC, Hajek P, Lycett D, Aveyard P: **Interventions for preventing weight gain after smoking cessation.** *Cochrane Database Syst Rev* 2012, **Jan 18**;1:CD006219.

For peer review only

16. Jolly K, Lewis A, Beach J, et al.: **Comparison of range of commercial or primary care led weight reduction programmes with minimal intervention control for weight loss in obesity: Lighten Up randomised controlled trial.** *BMJ* 2011, **343**:d7243.

17. Lycett, D., Aveyard, P., Farmer, A., Lewis, A. and Munafò, M. **Slimming World in Stop Smoking Services (SWISSS): study protocol for a randomized controlled trial**. *Trials*, 2013. *14*(1), p.182.

18. Kozlowski LT, Porter CQ, Orleans CT, Pope MA, Heatherton T. **Predicting smoking cessation with self-reported measures of nicotine dependence: FTQ, FTND, and HSI.** Drug and alcohol dependence. 1994 Feb 1;34(3):211-6.

19. Herdman M, Gudex C, Lloyd A, Janssen MF, Kind P, Parkin D, Bonsel G, Badia X. **Development and preliminary testing of the new five-level version of EQ-5D (EQ-5D-5L).** Quality of life research. 2011 Dec 1;20(10):1727-36.

20. Lycett D, Munafò M, Johnstone E, Murphy M, Aveyard P: **Associations between weight change over 8 years and baseline body mass index in a cohort of continuing and quitting smokers.** Addiction. 2011, 106: 188-196. 10.1111/j.1360-0443.2010.03136.x.

21. West R, Hajek P, Stead L, Stapleton J: **Outcome criteria in smoking cessation trials: proposal for a common standard.** Addiction 2005, 100(3):299–303.

22. West R, Hajek P: **Evaluation of the mood and physical symptoms scale (MPSS) to assess cigarette withdrawal.** Psychopharmacology 2004, 177:195–199.

23. Puchalski C, Romer AL: **Taking a spiritual history allows clinicians to understand patients more fully.** J Palliat Med 2000, 3:129–137.

For peer review only

24. Koenig HG, Cohen HJ, Blazer DG, et al.: **Religious coping and depression among elderly, hospitalized medically ill men.** Am J Psychiatry 1992, 149(12):1693–1700.

25. Lancaster, K.J., Carter‐Edwards, L., Grilo, S., Shen, C. and Schoenthaler, A.M.. **Obesity interventions in African American faith‐based organizations: a systematic review.** Obesity Reviews 2014, 15(S4), pp.159-176.

26. Gonzales D, Redtomahawk D, Pizacani B, et al.: **Support for spirituality in smoking cessation: results form a pilot survey.** Nicotine Tob Res 2007, 9(2):299–303.

27. Lindson-Hawley, N., Banting, M., West, R., Michie, S., Shinkins, B. and Aveyard, P., 2016. **Gradual versus abrupt smoking cessation: a randomized, controlled noninferiority trial.** Annals of internal medicine, 164(9), pp.585-592.

28. Devlin N, Shah K, Feng Y, Mulhern B, van Hout B. **Valuing health-related quality of Life: An EQ-5D-5L Value Set for England.** Health Economics. 2017;1-16

29. Bengtsson M. **How to plan and perform a qualitative study using content analysis,** NursingPlus Open 2016, Volume 2, Pages 8-14, ISSN 2352-9008, https://doi.org/10.1016/j.npls.2016.01.001.

30. Lader D: **A report on research using the National Statistics Opinions Survey produced on behalf of the NHS Information Centre for health and social care**. Office for National Statistics 2009.

31. Bush T, Levine MD, Beebe LA, et al. **Addressing weight gain in smoking cessation treatment: a randomized controlled trial.** Am J Health Promot. 2012 Nov-Dec;27(2):94-102. doi: 10.4278/ajhp.110603-QUAN-238.

32. Preloading Investigators. **Effects on abstinence of nicotine patch treatment before quitting smoking: parallel, two arm, pragmatic randomised trial**. *BMJ*, 2018. *361*, p.k2164.

32. Anderson JW, Konz EC, Frederich RC, Wood CL. **Long-term weight-loss maintenance: a meta-analysis of US studies**. The American journal of clinical nutrition. 2001 Nov 1;74(5):579-84.

33. Pan A, Wang Y, Talaei M, Hu FB, Wu T. **Relation of active, passive, and quitting smoking with incident type 2 diabetes: a systematic review and meta-analysis**. The lancet Diabetes & endocrinology. 2015 Dec 1;3(12):958-67.

34. Levine MD, Bush T, Magnusson B, Cheng Y, Chen X; **Smoking-Related Weight Concerns and Obesity: Differences Among Normal Weight, Overweight, and Obese Smokers Using a Telephone Tobacco Quitline**, Nicotine Tob Res, 2013. Volume 15, Issue 6, 1 June 2013, Pages 1136–1140, https://doi.org/10.1093/ntr/nts226

For peer review only

Characteristics	Decliners (n=128)	Participants (n=76)
**Age**	45.3 (14.1)	46.7 (13.5)
**Ethnicity (%)**		
**White British**	93% (n=119)	88% (n=67)
**White Irish**	1% (n=1)	
**White other**	2% (n=3)	
**Indian**	2% (n=2)	1% (n=1)
**White & Asian**		3% (n=2)
**Black – other**	1% (n=1)	8% (n=6)
**Unknown**	1% (n=2)	
**Gender**		(n=71)
**Female**	55% (n=70)	65% (n=46)
**Male**	45% (n=58)	35% (n=25)
**BMI category**	(self-report) (n=123)	(measured) (n=73)
**Underweight**	7% (n=8)	0% (n=0)
**Healthy Weight**	58% (n=71)	12% (n=9)
**Overweight**	35% (n=43)	34% (n=25)
**Obese**	1% (n=1)	53% (n=39)

Table 1. Characteristics of those who declined to take part and those who participated in the SWISSS trial

For peer review only

Characteristic (mean (SD)/% frequency)		Control (n=39)	Treatment (n=37)
**Age (years)**		50 (15)*	44 (12)*
**BMI (kg/m²)**		31.6 (6.3)*	29.3 (4.3)*
**FTND (scores range: 1-10)**		5.7 (2.2)	5.8 (2.1)
**MPSSM (scores range 7-35)**		12.7 (5.7)	12.8 (4.1)
**MPSSC (scores range 0-10)**		6.9 (1.7)	7.3 (1.1)
**MPSSP (scores range 3-15)**		4.2 (1.8)	3.9 (1.1)
**MPSST (scores range 10-60)**		23.8 (7.3)	24.1 (4.9)
**EQ-5D**	**Index**	0.759 (0.268)	0.848 (0.105)
**Value**		61 (23)	67 (15)
	**VAS (100 point scale)**		
**Using religion to cope score (scores range 1-10)**		(n=23)	(n=21)
		2.7 (2.6)	3.7 (2.9)
**Gender**		(n=38)	(n=33)
	**Male**	29%	44%
	**Female**	71%	56%
**Ethnicity**	**White**	92%	84%
**British**		5%	
	**White & Asian**		3%
	**Indian**	3%	13%
	**Unknown**		
**Highest Qualification**		(n=38)	(n=32)
	**No formal qualification**	18%	25%
	**GCSEs/O levels**	21%	28%
	**A levels**	42%	31%
	**Degree**	13%	9%
	**Higher Degree**	6%	7%
**Employment**		(n=38)	(n=32)
	**Employed**	55%	63%
	**Long term sick**	11%	0%
	**Looking after others**	3%	6%
	**Retired**	21%	13%
	**Unemployed**	8%	13%
	**Other**	2%	5%
**Religious**		(n=37)	(n=30)
	**No**	95%	87%
	**Yes**	5%	13%
**Supportiveness of religious community**		(n=20)	(n=14)
	**A lot**	5%	35%
	**A little**	15%	25%
	**Not at all**	80%	30%
**Impact of religious belief**		(n=11)	(n=10)
	**Comfort**	91%	100%
	**Stress**	9%	0%
**Pharmacotherapy used**		(n=16)	(n=15)
	**Nicotine Replacement Therapy**	38%	40%
**(NRT)**		62%	60%
	**Varenicline**		
**Family history of diabetes**		(n=36)	(n=31)
	**Yes**	25%	26%
	**No**	75%	74%
**Impaired glucose tolerance**		16%	19%
**Yes**		84%	81%
	**No**		
**Absolute Diabetes Prevalence**		(n=32)	(n=32)
	**Yes**	14%	13%
	**No**	86%	87%

GCSE: General Certificate of Secondary Education, O level: Ordinary level exams, A level: Advanced level exams.
*p<0.1 between control and treatment groups

Table2. Baseline characteristics by randomisation

Change in weight (kg) from baseline in quitters at:	Control	Treatment	Difference in weight change (treatment – control) mean [95% CI] adjusted for baseline weight as covariate
**4 weeks post quit**	(n=13)<br>0.7 (1.5)	(n=14)<br>-0.7 (1.8)	-1.2 [-2.5, 0.1]
**12 weeks post quit**	(n=8)<br>2.0 (1.9)	(n=12)<br>-1.3 (3.3)	-2.3 [-4.4, -0.1]
**26 weeks post quit**	(n=5)<br>2.3 (2.2)	(n=8)<br>-0.9 (2.5)	-3.1 [-6.9, 0.8]*

*Also adjusted for age (p<0.1 between treatment and control in 26 week quitters)

Table 3. Change in weight in quitters at 4, 12 & 26 weeks post quit

For peer review only

Change in Outcome from baseline (ITT-BOCF i.e. assumed no change)	At 4 weeks post quit			At 12 weeks post quit			At 26 weeks post quit		
	**Control**	**Treatment**	**Difference in change (treatment – control) OR / mean [95% CI]***	**Control**	**Treatment**	**Difference in change (treatment – control) OR / mean [95% CI]***	**Control**	**Treatment**	**Difference in change (treatment – control) % / mean [95% CI]***
**Abstinence rate**	13/39 (34%)	14/37 (38%)	1.2 [0.5, 3.1][1]<br>1.3 [0.4, 3.7][2]	8/39 (21%)	12/37(32%)	1.9 [0.7, 5.3][1]<br>2.9 [0.8, 10.1][2]	5/39 (13%)	8/37 (22%)	1.9 [0.6, 6.4][1]<br>3.2 [0.7, 14.4][2]
**MPSS – C (range 0-10)**	-0.8 (1.5)	-1.5 (2.1)	-0.7 [-1.6, 0.2][1]<br>-0.8 [-1.8, 0.1][2]	-0.7 (1.8)	-2.2 (2.7)	-1.5 [-2.6, -0.4][1]<br>-1.6 [-2.7, -0.5][2]	-0.7 (1.5)	-1.9 (2.5)	-1.3 [-2.2, -0.3][1]<br>-1.5 [-2.5, -0.4][2]
**MPSS – P (range 3-15)**	0.2 (1.3)	0.1 (1.0)	-0.1 [-0.7, 0.4][1]<br><0.1 [-0.6, 0.7][2]	<0.1 (1.6)	-0.1 (1.2)	-0.1 [-0.8, 0.5][1]<br><0.1[-0.7, 0.7][2]-	<0.1 (1.4)	-0.1 (1.1)	0.2 [-1.0, 1.4][1]<br>0.4 [-0.9, 1.7][2]
**MPSS- M (range 7-35)**	1.1 (3.8)	0.9 (4.9)	-0.2 [-2.4, 1.9][1]<br>0.1 [-2.2, 2.4][2]	-0.5 (3.7)	-1.4 (4.8)	1.0 [-3.0, 1.1][1]<br>-0.5 [-2.7, 1.7][2]	-0.6 (3.6)	-1.7 (5.0)	-1.3 [-3.6, 0.9][1]<br>-1.3 [-3.7, 1.2][2]
**Hunger (range 1-5)**	0.2 (0.8)	0.1 (0.9)	-0.1 [-0.5, 0.3][1]<br><-0.1[-0.5, 0.4][2]	0.1 (0.6)	0.1 (0.9)	<-0.1[-0.4, 0.3][1]<br><-0.1[-0.4, 0.3][2]	0.1 (0.5)	0.1 (0.9)	<-0.1[-0.4, 0.3][1]<br><-0.1[-0.4, 0.4][2]
**MPSS- T (range 10-60)**	0.5 (4.4)	-0.6 (6.0)	-1.1 [-3.6, 1.4][1]<br>-0.8 [-3.5, 1.9][2]	-1.3 (5.0)	-3.7 (7.4)	-2.6 [-5.6, 0.4][1]<br>-2.1 [-5.2, 1.1][2]	-7.1 (13.4)	-7.8 (14.5)	-0.8 [-7.5, 5.8][1]<br>-2.5 [-9.4, 4.3][2]
**EQ-5D Index Value**	0.008(0.065)	0.009(0.112)	0.006<br>[-0.053, 0.041][1]<br>-0.014<br>[-0.065, 0.037][2]	0.0165(0.086)	0.023(0.099)	0.006<br>[-0.039, 0.052][1]<br>0.008<br>[-0.040, 0.057][2]-	0.001 (0.069)	0.023(0.078)	0.003<br>[-0.062, 0.071][1]<br>0.006<br>[-0.059, 0.071][2]
**VAS (100 point scale)**	5.5 (20.9)	4.4 (16.2)	-2.1 [-9.4, 5.3][1]<br>-3.2 [-11.2, 4.7][2]	4.8 (12.7)	5.6 (12.7)	0.5 [7.5, 6.6][1]<br>-0.9 [-8.5, 6.7][2]	5.7 (15.2)	4.8 (11.8)	-1.1 [-7.7, 5.5][1]<br>-2.0 [--9.0, 5.0][2]
**Using religion to cope score (range 1-10)**	-0.3 (1.2)	-0.8 (1.9)	-0.6 [-1.7, 0.6][1]<br>-1.0 [-2.4, 0.4][2]	0.2 (0.5)	-0.3 (0.9)	-1.3[-2.3, -0.4][1]<br>-1.5 [-2.8, -0.3][2]	0.5 (1.2)	<0.1 (1.8)	-0.2 [-0.6, 0.2][1]<br>-0.2 [-0.7, 0.2][2]

[1] Unadjusted regression analysis

[2]Regression analysis adjusted for baseline BMI and age

Table 4. Change in abstinence and exploratory outcomes within and between the control and intervention group

Figure 1. Flow of participants through the trial

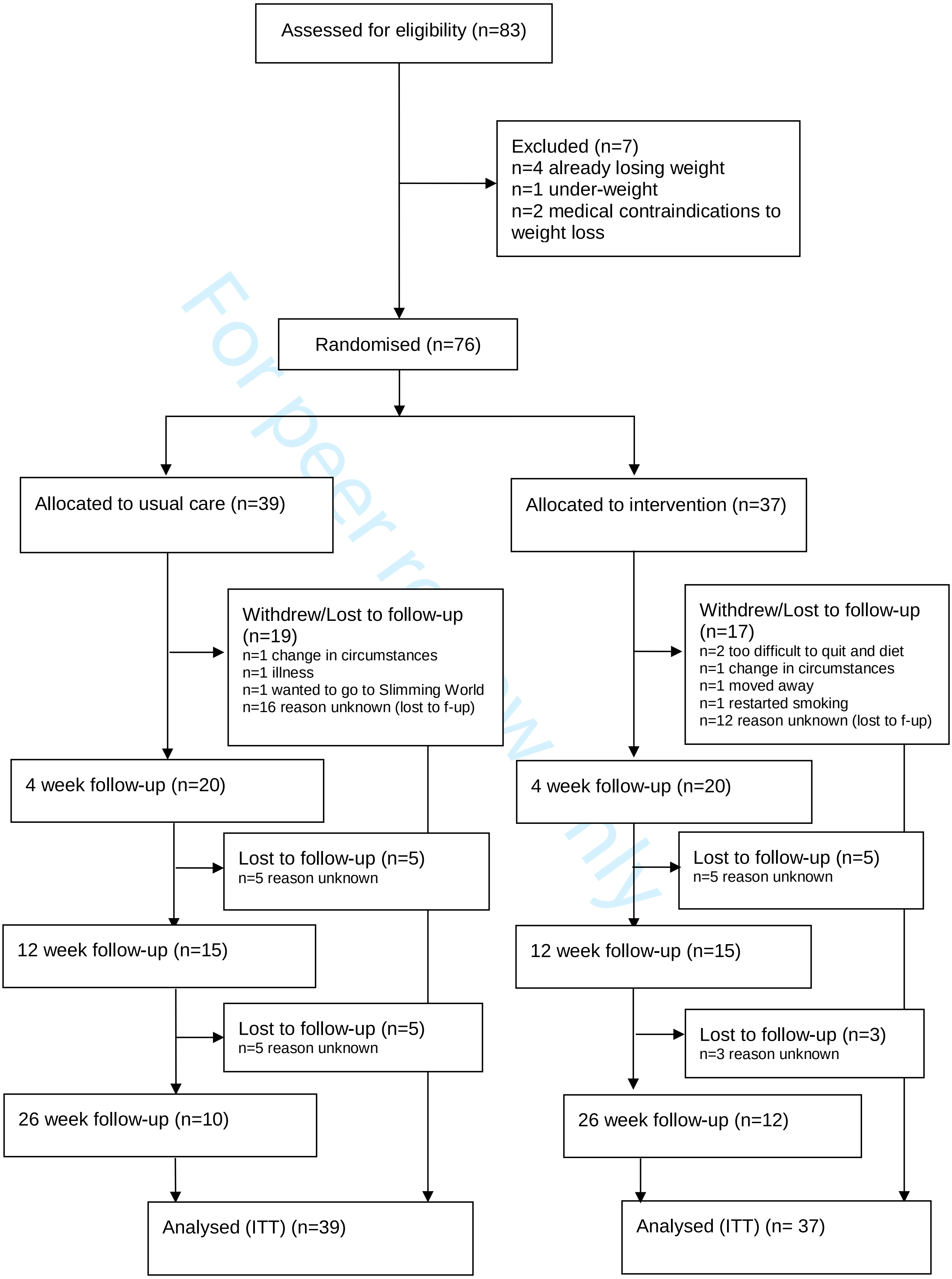

For peer review only

Code (number of times featured in data)	Sub theme	Theme
Good trial (2)	Positive aspects	Aspects related to research processes
Easy to implement (4)		
Additional [paper] work (3)	Time and additional effort on part of practitioner and patient	
Patient time (2)		
Practitioner time (7)		
Discussion of research viewed negatively (1)	Engaging in research was viewed positively and negatively	
Discussion of research viewed positively (1)		
Patient disappointment with randomisation (3)	Disappointment for participants and practitioners (and misunderstanding for practitioner) regarding randomisation	
Practitioner disappointment with randomisation (2)		
Slimming World vouchers seen as an positive incentive (2)		
Discussion of weight viewed negatively (1)	Discussing weight was seen as a positive and negative thing	An opportunity to address the weight issues
Discussion of weight viewed positively (5)		
Lack of patient interest in weight (4)	Patient interest and attendance	Poor participant interest and attendance
Patient drop out, transient population (3)		
Few people wanting to quit (2)		

Supplementary Table 1. Content analysis of open comments from practitioners at the end of the trial

For peer review only

Supplementary Figure 1. Reasons for declining to take part in SWISSS (more than 1 choice possible)

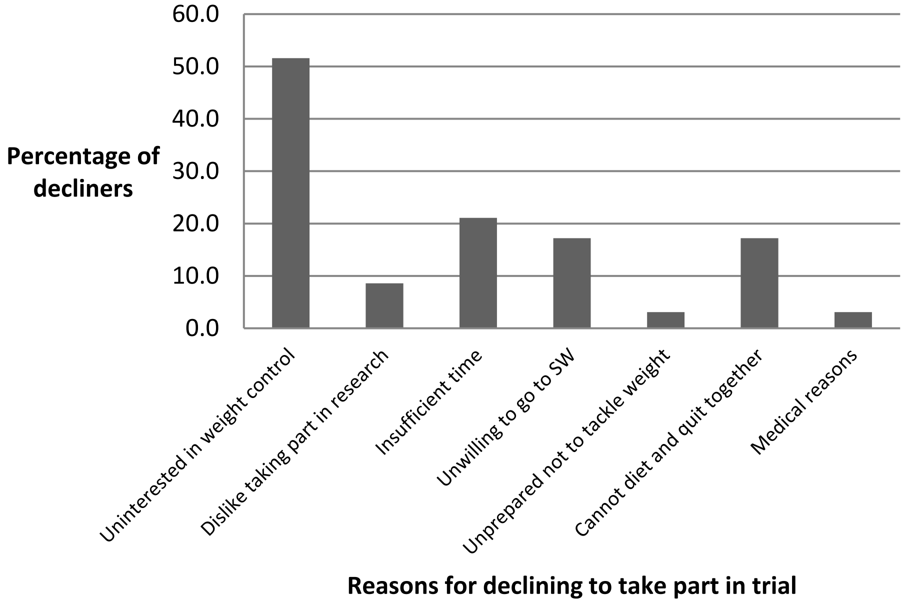

For peer review only

CONSORT 2010 checklist of information to include when reporting a randomised trial*

Section/Topic	Item No	Checklist item	Reported on page No
**Title and abstract**			
	1a	Identification as a randomised trial in the title	1
	1b	Structured summary of trial design, methods, results, and conclusions (for specific guidance see CONSORT for abstracts)	2-3
**Introduction**			
Background and objectives	2a	Scientific background and explanation of rationale	4
	2b	Specific objectives or hypotheses	5
**Methods**			
Trial design	3a	Description of trial design (such as parallel, factorial) including allocation ratio	5
	3b	Important changes to methods after trial commencement (such as eligibility criteria), with reasons	N/A
Participants	4a	Eligibility criteria for participants	6
	4b	Settings and locations where the data were collected	5
Interventions	5	The interventions for each group with sufficient details to allow replication, including how and when they were actually administered	6-7
Outcomes	6a	Completely defined pre-specified primary and secondary outcome measures, including how and when they were assessed	7
	6b	Any changes to trial outcomes after the trial commenced, with reasons	N/A
Sample size	7a	How sample size was determined	9
	7b	When applicable, explanation of any interim analyses and stopping guidelines	9
Randomisation:			
Sequence generation	8a	Method used to generate the random allocation sequence	7
	8b	Type of randomisation; details of any restriction (such as blocking and block size)	7
Allocation concealment mechanism	9	Mechanism used to implement the random allocation sequence (such as sequentially numbered containers), describing any steps taken to conceal the sequence until interventions were assigned	7
Implementation	10	Who generated the random allocation sequence, who enrolled participants, and who assigned participants to interventions	7
Blinding	11a	If done, who was blinded after assignment to interventions (for example, participants, care providers, those	N/A

For peer review only

For peer review only - http://bmjopen.bmj.com/site/about/guidelines.xhtml

		assessing outcomes) and how	
	11b	If relevant, description of the similarity of interventions	N/A
Statistical methods	12a	Statistical methods used to compare groups for primary and secondary outcomes	9
	12b	Methods for additional analyses, such as subgroup analyses and adjusted analyses	9
**Results**			
Participant flow (a diagram is strongly recommended)	13a	For each group, the numbers of participants who were randomly assigned, received intended treatment, and were analysed for the primary outcome	Figure 1
	13b	For each group, losses and exclusions after randomisation, together with reasons	Figure 1
Recruitment	14a	Dates defining the periods of recruitment and follow-up	8
	14b	Why the trial ended or was stopped	9
Baseline data	15	A table showing baseline demographic and clinical characteristics for each group	Table 2
Numbers analysed	16	For each group, number of participants (denominator) included in each analysis and whether the analysis was by original assigned groups	Figure 1
Outcomes and estimation	17a	For each primary and secondary outcome, results for each group, and the estimated effect size and its precision (such as 95% confidence interval)	Table 3 and table 4
	17b	For binary outcomes, presentation of both absolute and relative effect sizes is recommended	Table 4
Ancillary analyses	18	Results of any other analyses performed, including subgroup analyses and adjusted analyses, distinguishing pre-specified from exploratory	9
Harms	19	All important harms or unintended effects in each group (for specific guidance see CONSORT for harms)	3
**Discussion**			
Limitations	20	Trial limitations, addressing sources of potential bias, imprecision, and, if relevant, multiplicity of analyses	15
Generalisability	21	Generalisability (external validity, applicability) of the trial findings	16
Interpretation	22	Interpretation consistent with results, balancing benefits and harms, and considering other relevant evidence	16
**Other information**			
Registration	23	Registration number and name of trial registry	3
Protocol	24	Where the full trial protocol can be accessed, if available	5
Funding	25	Sources of funding and other support (such as supply of drugs), role of funders	18

*We strongly recommend reading this statement in conjunction with the CONSORT 2010 Explanation and Elaboration for important clarifications on all the items. If relevant, we also recommend reading CONSORT extensions for cluster randomised trials, non-inferiority and equivalence trials, non-pharmacological treatments, herbal interventions, and pragmatic trials. Additional extensions are forthcoming: for those and for up to date references relevant to this checklist, see www.consort-statement.org.

For peer review only - http://bmjopen.bmj.com/site/about/guidelines.xhtml

BMJ Open

Referral to Slimming World in UK Stop Smoking Services (SWISSS) versus Stop Smoking Support Alone on Body Weight in Quitters: Results of a Randomized Controlled Trial

Journal:	*BMJ Open*
Manuscript ID	bmjopen-2019-032271.R2
Article Type:	Original research
Date Submitted by the Author:	01-Dec-2019
Complete List of Authors:	Lycett, Deborah; Coventry University<br>Aveyard, Paul; University of Oxford, Primary Care Health Sciences<br>Farmer, Andrew; University of Oxford, Department of Primary Care Health Sciences<br>Lewis, Amanda; University of Bristol, Population Health Sciences Bristol Medical School<br>Munafo, Marcus; University of Bristol, Experimental Psychology
**Primary Subject Heading**:	Public health
Secondary Subject Heading:	Smoking and tobacco
Keywords:	weight, smoking, obesity

SCHOLARONE™
Manuscripts

Referral to Slimming World in UK Stop Smoking Services (SWISSS) versus Stop Smoking Support Alone on Body Weight in Quitters: Results of a Randomized Controlled Trial

For peer review only

Deborah Lycett PhD[1*]

[*] Corresponding author

Email: deborah.lycett@coventry.ac.uk

Paul Aveyard PhD[2]

Email: paul.aveyard@phc.ox.ac.uk

Andrew Farmer DM[2]

Email: andrew.farmer@phc.ox.ac.uk

Amanda Lewis PhD[3]

Email: amanda.lewis@bristol.ac.uk

Marcus R. Munafò PhD[4]

Email: marcus.munafo@bristol.ac.uk

[1] Faculty of Health & Life Sciences, Coventry University, Priory Street, Coventry CV1 5FB, UK. [2] Nuffield Department of Primary Health Care Sciences, University of Oxford, Radcliffe Observatory Quarter, Woodstock Road, Oxford OX2 6GG, UK. [3]Population Health Sciences Bristol Medical School, University of Bristol, Canynge Hall (Room G.08a), 39 Whatley Road

Bristol, BS8 2PS. [4]MRC Integrative Epidemiology Unit, UK Centre for Tobacco and Alcohol Studies, School of Experimental Psychology, University of Bristol, 12a Priory Road, Clifton, Bristol BS8 1TU, UK.

ABSTRACT

Introduction

Most people who stop smoking gain weight. Dietary modification may seem an obvious solution, but food restriction may increase cigarette craving and smoking relapse.

Trial Design

An unblinded parallel randomised controlled trial

Methods

Participants were adult smokers with a body mass index (BMI) greater or equal to 23 kg/m^2.

Setting was NHS commissioned Stop Smoking Services,

Interventions were referral to a commercial weight management programme, plus stop smoking support (treatment group), compared to stop smoking support alone (control group).

Objective was to compare weight change between interventions in smoking abstainers and not abstinent rates in all.

Primary outcome was change in weight (kg) at 12 weeks.

Randomisation sequence was computer generated and concealed until allocation.

For peer review only

Results

Seventy-six participants were recruited, 37 were randomized to the treatment group and 39 to the control group.

Change in weight was analysed in long-term abstainers (13 treatment, 14 control) only because the aim was to prevent weight gain associated with smoking cessation.

Abstinence was analysed on an intention-to-treat basis (37 treatment, 39 control).

At 12 weeks weight gain was less in the treatment than the control group with an adjusted mean difference of -2.3kg 95% CI [-4.4, -0.1]. Craving scores were lower (MPSS-C -1.6 [-2.7, -0.5]) and quit rates were higher in the treatment than the control group (32% vs 21%), although the trial was not powered to superiority in cravings and quit rates..

No adverse events or side effects were reported.

Conclusion

In people who are obese and want to quit smoking, these data provide modest encouragement that providing weight management at the time of quitting may be helpful. Those who are not obese, but who are informed about potential weight gain during their quit attempt, were uninterested in a weight management programme.

TRIAL REGISTRATION

Current Controlled Trials ISRCTN65705512

http://www.controlled-trials.com/ISRCTN65705512

For peer review only

ARTICLE SUMMARY

Strengths and limitations of this study

- The first study investigating referral to a commercial weight loss programme during smoking cessation.
- Computer generated randomisation with allocation concealment.
- Weight measured and abstinence verified by exhaled CO.
- Small study with poor recruitment but reverse power calculation showed greater than 80% power to detect change in primary outcome of weight.

INTRODUCTION

Weight gain is a well-known consequence of smoking cessation [1, 2]. A meta-analysis of weight data, from randomised controlled trials, at one year post quit date showed that in untreated quitters there is a mean increase of 4-5kg. However, variation in weight change is large, with about 16% of quitters losing weight and 13% gaining more than 10kg [3].Nonetheless with the majority of people gaining weight smokers may be deterred from attempting to quit and this weight gain may offset some advantages of giving up smoking. Smoking cessation-related weight gain partly explains the finding that the incidence of type 2 diabetes is increased by up to 73% in the years after cessation [4-6], there is a 30% increased risk of hypertension [7], and a deterioration in glycaemic control in those with diabetes [8] who quit compared to those who continue to smoke. Despite these concerns weight gain in quitters does not negate the risk reduction of cardiovascular morbidity and mortality in those who successfully quit smoking [9]. There is some evidence to suggest the benefit may be

For peer review only

mitigated [10] but the number of incident cases in higher weight gain categories are too small to draw conclusions. Nonetheless, many people find this weight gain unacceptable and the greatest danger is it may deter individuals from quitting [11]. Yet, restricting food intake may limit successful quitting as hunger increases urges to smoke [12, 13] and people who gain most weight quit more successfully [14].

An individually tailored plan to reduce energy intake and increase exercise, with regular monitoring and adaptation of individual goals, appears to be the most promising way to prevent cessation related weight gain with the least risk of impeding a quit attempt [15]. Commercial weight management providers (CWMPs) offer this type of support and are an effective way for many to lose weight through referral from National Health Service (NHS) primary care [16]. Therefore, referral to a CWMP on prescription from the NHS Stop Smoking Services may be an effective way to prevent smoking-related weight gain without a detrimental effect on quitting success.

This study compares referral to a CWMP as part of stop smoking support with stop smoking support alone on changes in body weight in quitting smokers.

METHODS

Design

This was a parallel group, individually randomized controlled trial (RCT) to compare standard stop smoking behavioural support with an intervention that, in addition to providing standard stop smoking support, included personalised weight management support, provided by Slimming World.

For peer review only

This was an open label trial, as blinding patients and smoking cessation advisors to allocation to intervention or control was impossible. The primary outcome was objectively measured weight and therefore the scope for bias is limited. Stop smoking advisors, who weighed participants, did not provide the weight control support and were unlikely to have a vested interest in interpreting weight change favourably. Full details of the methods for this trial can be found in the protocol paper [17] but are summarized below.

Setting

NHS commissioned Stop Smoking Services were first recruited from Bristol and North Somerset Primary Care Trusts (PCTs). Stop smoking services were invited to take part by letter and a follow-up phone call 2 weeks later. Clinics were only eligible to participate if they had 2 new service users coming through their doors each week. Due to poor recruitment we invited more clinics (n=30) to take part in the study by widening our area further across the West Midlands and South-West England. Additionally we extended the planned duration of recruitment from 10 to 17 months, which was as far as our budgetary restraints would allow.

All individuals attending the stop smoking services were invited to take part in the trial at their first visit when they set their quit date. At the second visit those wanting to participate in the trial gave their consent and were randomised to the control or intervention group. Those randomised to the intervention were given a referral to Slimming World, a location and a start date as near to their quit date. Those who declined to take part were invited to provide a reason.

Baseline measures were taken after randomization and included age, gender, socioeconomic status, ethnicity, religion, Fagerström Test for Nicotine Dependence (FTND) [18] and all dimensions of EQ-5D (EuroQol, Rotterdam, Netherlands) as a measure of health-related quality of life [19] presented as index values.

For peer review only

Participants

Daily adult smokers with expired CO ≥10 ppm and a body mass index (BMI) greater or equal to 23 kg/m^2 were included. Pregnant smokers, those with a BMI < 23kg/m^2, those currently losing weight, or where weight loss was contraindicated, were excluded.

Participants with an ideal BMI, as well as those who are overweight, were included in the trial. This is because help is needed not only for quitting smokers who are overweight, but also for those of a healthy weight to prevent the weight gain that is associated with smoking cessation. Without engaging in weight management strategies to counteract the physiological and behavioural responses to smoking cessation, 85% of people who quit smoking will gain weight, regardless of their starting BMI [20].

Randomization

A randomization sequence was generated using computer software. Stratified randomisation by stop smoking advisor with blocking within each stratum was used to ensure balance. The blocks were randomly ordered blocks of 2, 4 and 6. Participants were randomised 1:1 to usual care or Slimming World with usual care. Stop smoking advisors were unaware of the randomisation sequence; they opened sealed, numbered, opaque envelopes in turn.

Interventions and comparisons

Trial treatment providers

Trained NHS stop smoking advisors provided participants with standard smoking cessation support in both arms of the trial. This was withdrawal oriented behavioural support focusing

For peer review only

on key behavioural change techniques, and a prescription of nicotine replacement or varenicline to relieve withdrawal symptoms. Support was on a weekly basis beginning two weeks before and until four weeks after quit day. Weight control support was provided by trained weight management counsellors employed by Slimming World. Slimming World is a CWMP which follows the National Institute for Health and Care Excellence (NICE) criteria for clinical practice. It is commissioned by the NHS to provide a weight loss service to patients. Slimming World was the CWMP of choice as it works with populations to prevent excessive weight gain rather than solely focusing on weight loss, this was pertinent to preventing cessation related weight gain.

Intervention

In addition to usual care, participants attended Slimming World for 12 weeks receiving support to lose weight or prevent weight gain. Slimming World consultants typically agree weight management targets with their members at the first appointment and then work with their members to achieve this through regular weight monitoring, controlled amounts of high energy foods and personal eating plans.

Control

Participants were encouraged to quit smoking first, before tackling weight. Stop smoking advisors advised satiating hunger with healthy foods, which is standard care, but did not provide detailed or personalized advice regarding weight management.

Outcome measures

Primary outcome measure:

For peer review only

Change in measured weight from baseline to 12 weeks post-quit day in abstinent smokers. Abstinence was prolonged abstinence, defined according to the Russell Standard [21].

Secondary outcome measures:

- Weight at 4 weeks post-quit date.
- Weight at 6 months from baseline.
- Abstinence at 4, 12 and 26 weeks
- Participant acceptability measured quantitatively by response, attrition rates and a decliner's reasons for not participating.
- Feasibility of running the trial within the Stop Smoking Services was measured by an open text questionnaire asking for comment.

Exploratory outcomes measures:

- Change in EQ-5D index values.
- Cigarette withdrawal symptom scores as measured by the mood and physical symptoms scale (MPSS) [22].
- Associations of change in religious engagement (adapted from CSI-MEMO [23]) and religious coping (religious coping index (RCI) [24]) as positive use of individual's spiritual reserve to change weight [25] and smoking behaviour [26] is increasingly evidenced.

For peer review only

Statistics and data analysis

Sample size

Difficulty in recruiting resulted in it being economically unaffordable to continue the trial; therefore, the decision was made to stop the trial early.

Following the full analysis of our outcome data we conducted reverse power calculations. This showed that we had >80% power, at an alpha error rate of 0.05, to detect the observed differences in weight change in our sample at 12 weeks between the treatment and control groups. Given a large observed difference in quit rates between the control and treatment groups we also had 90% power at an alpha error rate of 0.05 to determine non-inferiority, using a non-inferiority limit of 19% [27].

Our original sample size had been conservative assuming we would see 20% weight gain in the treatment group, rather than the weight loss we actually saw. This would have required 32 quitters in each trial arm at 12 weeks; instead, we achieved 8-10 quitters at that time point. To achieve 32 quitters in each trial arm, based on a conservative estimate that only 20% of those recruited would be quit at 12 weeks, we wanted to recruit 160 per trial arm. Instead, we only recruited 76 smokers in total, but the quit rates in the treatment group were higher than expected.

Analysis

The change in weight was analysed in long-term abstainers only because only long-term abstainers gain more weight over time than the general population and because the aim was to prevent weight gain on smoking cessation [20]. Therefore, as this comparison was made in abstainers only, and not those randomised to control or treatment groups, investigation of

For peer review only

baseline differences was conducted between these groups. To identify likely confounding variables selected baseline characteristics of age, weight, BMI, FTND, gender, ethnicity and pharmacotherapy were compared, between quitters at 4, 12 and 26 weeks post quit in the control and treatment groups, using t-tests and chi-squared statistics. Weight change in the intervention and the control arm of the study was presented descriptively using means and SDs and the mean difference and 95% confidence intervals (CI) between the two arms calculated using multiple linear regression with adjustment for baseline differences.

Abstinence was analysed on an intention-to-treat basis, assuming all those not present at follow-up had relapsed [21]. As we recruited fewer people than intended, we could not assume effective randomisation and so investigated whether there were any differences in baseline characteristics between the control and treatment groups. Adjustment for these was then made using logistic multiple regression to obtain an odds ratios for becoming abstinent.

The quality of life (EQ-5D) was presented as index values calculated from interim scoring for the EQ-5D-5L spreadsheet for UK values [28], means and SD of the EQ-5D visual analogue scale (VAS) were also presented following the EQ-5D-5L reporting guide.

The change in exploratory measures were presented with descriptive statistics and analysed using multiple linear regression. We also reported descriptive data about the acceptability of the intervention, supplemented by comments from questionnaires completed by stop smoking service practitioners. We analysed these using manifest content analysis of a broad surface structure reflecting what was literally said [29]. Open comments were broken down into individual codes of meaning then built up into sub themes and themes which summarised the data.

For peer review only

Ethics approval

The study was approved by Cornwall and Plymouth NHS Research Ethics Committee. The trial was conducted in accordance with the recommendations for physicians involved in research on human subjects adopted by the 18th World Medical Assembly, Helsinki, 1964, and later revisions. All participants gave their written informed consent to participate in the study.

Patient and Public Involvement

A patient advisory group were asked to comment on the concept of the study and the perceived acceptability of the proposed intervention, feedback was positive and so study plans progressed.

Participant reimbursement for attending follow-up visits

Participants received £5 payment for travel and inconvenience at the 12 and 26 week follow-up visits.

RESULTS

Potential Participants

Thirty different stop smoking clinics in the West Midlands and South-West of England inclusion criterion of treating at least two new service users attending a clinic each week. Potentially over 4400 participants could have been accessed over our 17 month recruitment period and invited to take part in the trial. Of those who declined to take part 128 individuals provided us with a reason. Eighty-three people agreed to take part and were screened for eligibility, 7 were ineligible (4 were already losing weight through attending a commercial weight loss provider) and 76 individuals were randomised. Of these, 37 were randomized to the treatment group and 12 returned for the 26 week follow-up, while 39 were randomized to

For peer review only

the control group of which 10 returned for the 26 week follow-up. The majority of those not responding to follow-up did so in the first four weeks of the trial and continued to ignore requests thereafter. Eight withdrew from the trial for various reasons, one individual in the control group did so to join Slimming World and two individuals in the intervention group said it was too difficult to quit and diet at the same time, another reported returning to smoking (Figure 1).

Characteristics of 'decliners' and participants

Those who provided a reason for declining to take part (decliners) and those who agreed to participate were of a similar mean age and mostly white British people. Women were more likely than men to enrol in the trial, but the most striking difference related to baseline weight status. Practically everyone who was obese agreed to join the trial, while a minority in the other BMI groups did so (Table 1).

Over half those who declined to take part did so because they were not interested in weight control. (Proportionally more who gave this reason had a healthy weight rather than being overweight (data not shown).) Over 20% of reasons for declining were because people did not have time. 17% said they were not prepared to go to Slimming World, similar numbers said they could not quit smoking and diet at the same time. Medical reasons and reasons related to the research process such as the principle of taking part or being randomised to the control were not commonly reported (Supplementary Figure 1).

Baseline Characteristics of participants in the treatment and control group at baseline

Baseline characteristics of participants were investigated between the control and treatment groups as failure to recruit to target sample size could have resulted in an imbalance. Those in the control group were significantly younger and with a higher BMI than the treatment group

For peer review only

(Table 2). and adjustment was made for this in the analysis of outcomes.

Weight Change During Abstinence

Among abstinent participants, there was a steady mean weight gain over time in the control group, whereas weight reduced at each time point in the treatment group. In those who were quit at 4 week follow-up there was an adjusted mean difference of -1.2kg [-2.5, 0.1] weight change between those in the intervention group and those in the control group. At 12 weeks the difference was greater --2.3kg [-4.4, -0.1] and some of this initial difference in weight loss between the trial arms was maintained at 26 week follow-up (-3.1kg [-6.9, 0.8]). (Table 3). No confounding variables were identified in the quitters between treatment and control groups at week 4 and 12 post-quit (results no shown), age was a potential confounding variable for those who remained quit at 26 weeks and adjustment for age was included in the analysis of difference in weight between the trial arms.

Change in Abstinence (Intention-to-treat analysis)

In the control group 13 of 39 participants (34%) were abstinent at 4 weeks and in the treatment group 14 of 37 (38%) were abstinent. By 12 weeks 8 (21%) participants in the control and 12 (32%) in the treatment group remained abstinent. At 26 week follow-up 5 (13%) participants in the control group remained abstinent and 8 (22%) in the treatment group.

Change in Exploratory Outcomes

Change in the MPSS craving domain (MPSS-C) at all time-points showed a greater reduction in the treatment than the control group, with mean adjusted differences ranging from 0.7 to 1.9 on a scale of 0-10. In the mood domain, MPSS-M, in the longer term (26 week follow-up)

For peer review only

there was a greater reduction in negative affect in the treatment group than the control group but the adjusted mean difference was negligible -1.3 [-3.7, 1.2] on a scale between 7 and 35. The effect on hunger and MPSS-P within and between the trials arms was negligible at all time-points. There was negligible change in religious coping and the Index Values of EQ-5D between the trial arms. The visual analogue scale (VAS) for quality of life improved between 4-6% in both groups across all time points (Table 4).

Practitioners' comments on trial delivery

Three main themes came out of the content analysis of practitioners' comments, these were 'Aspects related to research processes', which included positive aspects such as ease of implementation, however additional time and paperwork particular on the part of the practitioner, and also the perceived burden for the participant, were viewed negatively. Randomisation to the control group disappointed both the participant and the practitioner; practitioners reported viewing Slimming World vouchers as an incentive and this suggested misunderstanding of the nature and value of randomization and the need to promote the control arm as a good alternative. Such attitudes may have fuelled disappointment and subsequent drop out. The second theme was 'An opportunity to address weight issues', this opportunity was predominantly seen as a positive aspect of the trial and practitioners anticipated this would generate great interest from participants. However, practitioners were somewhat surprised that participants did not seem to want to address weight, there was mention that fewer people at the time of this trial were seeking stop smoking services than expected, and high rates of drop out were also problematic. These comments led to the third theme 'Poor participant interest and attendance' which characterised experience of delivering the trial (Supplementary Table 1).

For peer review only

DISCUSSION

Summary of results and consistency with other findings

The aim of the study was to prevent cessation related weight gain – which as discussed in the introduction has both clinical and psychological consequences, the most harmful of which may be considered as relapse to smoking. The trajectory of cessation related weight gain was seen by a consistent weight increase in the control group. In comparison, the treatment group lost weight at each of these time points. Referral to the 12-week Slimming World programme plus usual stop smoking support achieved significantly less weight gain than usual stop smoking support alone. This adjusted difference (-2.3kg[-4.4, -0.1]) was apparent at 12 weeks post-quit and is slightly greater than that seen in other trials comparing personalised weight management to usual care (-1.1kg [-1.9, -0.3]) [14]. This was partially sustained at 26 weeks post-quit (-3.2kg [-6.9, 0.8]).

However, we were unable to recruit to our target sample size and these results lack precision.

Percentage quit was no worse in the treatment than the control group; it was slightly higher in the treatment group but the trial was underpowered to test superiority. MPSS craving scores dropped further in the treatment than the control group (>1point difference on 10point scale at 12 and 26 weeks). Hunger ratings were similar in both groups across all time points.

Comparing our findings with the meta-analyses of personalised weight management versus no weight management at 26 weeks [15], our study achieved a similar quit rate in our treatment group (22%) as in the control groups of other studies (21%). However the quit rates in our

control group was lower than in these other studies (13% vs 19%) which may account for the difference in quit rates we saw here.

Taken together these results suggest that referral to a commercial weight loss provider, such as Slimming World, at the time of a quit attempt can prevent cessation-related weight gain without negatively impacting a quit attempt. However, we cannot draw firm conclusions given our small sample size.

Limitations

The greatest limitation to this study was the difficulty in recruiting which led to the small sample size. Anecdotally stop smoking advisors often cite weight gain is raised as a concern in clinics and were anticipating a high uptake for this trial, however we found that when given the opportunity participation was poor and more than 50% of people who gave a reason for declining reported it was because they were 'uninterested in weight control'.

While we do not know the reasons for everyone who declined, these results suggest that weight gain may not be as much as a barrier to quitting smoking as is commonly believed, particularly in those who are not obese. However, all those who were invited to participate in the trial had already decided to quit smoking and were looking for support in that goal. In addition, people who were already losing weight or attending a commercial weight loss programme were ineligible to take part (9% the 44 screened for eligibility). The Office for National Statistics reports weight gain only deters 5% of people from quitting and only 3% of people cite it as a reason for returning to smoking [30]. In light of this, and in view of the evidence that the cardiovascular benefits of quitting are evident despite weight gain [9, 10], we can reassure quitters who are worried about gaining weight that they need not be unduly concerned. In fact, evidence from a randomised control trial in the US (350 quitters at 6 months) suggests that

For peer review only

counselling quitters to reduce their weight concerns (through CBT telephone counselling compared to standard quit advice) leads to similar rates of quitting and a significant difference in weight change in favour of the counselling group (-0.5kg vs 1.0kg). Such counselling involved identifying and addressing maladaptive thoughts about body shape and weight, discouraged restrictive dieting, educated quitters on the relationship between weight gain and smoking cessation and encouraged an acceptance of moderate weight gain after quitting [31].

The small sample size also means that we cannot assume effective randomisation, therefore we cannot account for any unmeasured of residual confounding which may have otherwise been explained the results we found.

The trial was also limited by a short-term follow-up at 6 months post-quit (3 months post treatment intervention), therefore the long term consequences of the effect on weight gain prevention are unknown. However, meta-analysis data from randomised controlled trials suggests that 17% of weight lost is maintained 5 years after the end of moderate weight management interventions [32] so we might expect a similar trajectory here. This intervention in stop smoking services may therefore have limited impact in the long-term prevention of weight gain associated with smoking cessation. It is also important to note that the magnitude of weight change is relatively small (mean reduction 1.1kg at 12 weeks) only 1.3% weight loss considering the mean weight of all participants at baseline was 86.9kg.

Another limitation is the high rate of loss to follow-up in both the control and treatment arms of the study. The provision of free weight management advice did not deter attrition. High attrition is common in smoking cessation trials and is usually due to participants returning to smoking and as such the standard approach in analysis is to assume this [21]. However, our trial showed higher loss to follow-up compared to other smoking cessation trials [33], this may

For peer review only

have been due to staff time pressures reported by practitioners which may have limited their continued attempts to make contact with those who did not attend.

Additionally, not all data was fully completed at baseline, there were disproportionally more missing values in the treatment than the control arm but we have no reason why this might be the case.

Our trial did show that a few people were interested in preventing weight gain by attending a behavioural weight loss programme, and those who wanted to take part in the trial were more likely to be female and more likely to be obese. This is consistent with other findings that suggest women who are obese are likely to be the most weight concerned [34]. Therefore, the generalisability of our findings are largely limited to these who are already overweight or obese.

For individuals who feel unable to make a quit attempt without concurrently controlling their weight, this trial suggests that referral to a behavioural weight loss programme may be a pragmatic option within stop smoking clinics.

Implications for future trials to prevent cessation related weight gain:

- Stop smoking service practitioners consider a relatively straightforward trial delivered with support to impose undue burdens on their time, limiting their willingness to approach potential participants. This is mostly due to the paperwork concerned with consent and follow-up. In low-intensity low-risk trials, we need to develop a method for achieving this that does not impose the same burdens as trials of much riskier interventions. In the meantime, triallists need to understand this and make fewer demands by redesigning the trials.

For peer review only

- Most people who are stopping smoking are uninterested in active behavioural support to prevent weight gain. However, where people already are overweight to the extent of being obese, most people were prepared to enter the trial and future trials may target this population.
- Practitioners and participants quickly become despondent if not randomised to their choice of intervention, almost always the intervention group. More intensive training for practitioners in how to present trials to create equipoise is likely to be needed. There was a suggestion that quit rates were higher in the intervention than the control group and it may be helpful to explore qualitatively whether unhappiness at weight gain explains this.

Conclusion

Referral to the 12-week Slimming World programme plus usual stop smoking support achieved weight loss compared with weight gain seen with usual smoking cessation support alone throughout the quit attempt as far as 26 weeks. Quit rates, cravings for cigarettes, feelings of hunger and quit rates did not seem to be adversely affected although the recruited sample was too small to confirm this.

Recruitment into a trial of stop smoking services referred into a behavioural weight loss programme was poor. They most common reason for declining to participate was no interest in controlling weight.

ABBREVIATIONS

BMI: body mass index; CI: confidence interval; CRF: case report form; CWMP: commercial weight management providers; FTND: Fagerström Test for Nicotine Dependence; GCP: Good Clinical Practice; ICH GCP: International Conference on Harmonisation Good Clinical

For peer review only

Practice; LES: local enhanced services; MPSS: mood and physical symptoms scale; NIHR: National Institute for Health Research; NHS: National Health Service; NICE: National Institute for Health and Care Excellence; PC-CRTU: Primary Care Clinical Research and Trials Unit; R&D: Research and Development; RCI: religious coping index; RCT: randomized controlled trial; REC: research ethics committee; SOP: standard operating procedure; SPCR: School for Primary Care Research; UKCTCS: UK Centre for Tobacco Control Studies.

COMPETING INTERESTS

DL has received hospitability from manufacturers of smoking cessation products, Pfizer Ltd, Tadworth, UK. DL's institution during the active phase of the trial has received smoking cessation products for use in a clinical trial from Johnson & Johnson Ltd, New Brunswick, NJ, USA. DL has received Slimming World membership vouchers for use in this trial. DL has received expenses and consultancy fees from the NHS and Universities for teaching about cessation-related weight gain. DL has received grant funding from UKCTCS and the NIHR-SPCR for research relating to cessation-related weight gain. PA is an NIHR senior investigator and is funded by NIHR Biomedical Research Centre and the CLAHRC, Oxford. AF is an NIHR Senior Investigator and receives funding from NIHR Oxford Biomedical Research Centre. AL has received hospitality from Weight Watchers. In the last 5 years, MM has received grant funding from Pfizer Ltd and varenicline, for research purposes.

For peer review only

AUTHOR CONTRIBUTIONS

DL, PA, AF, AL and MM contributed to the design of the trial and interpretation of the data. DL conducted the analysis and drafted the manuscript. DL, PA, AF, AL and MM provided critical review on the drafts and are accountable for the work.

FUNDING

This work was supported by funding of the salary for DL, provided by a fellowship from the NIHR-SPCR during the active trial period. Additional funds to set up the trial and buy equipment were provided by the NIHR-SPCR and UKCTCS. The UKCTCS is one of five UK Public Health Research Centres of Excellence. We also gratefully acknowledge funding from the British Heart Foundation, Cancer Research UK, the Economic and Social Research Council, Medical Research Council and the Department of Health, under the auspices of the UK Clinical Research Collaboration. AL's salary was paid by NIHR-SPCR during the trial period, and the salaries of AF, MM and PA were paid by their respective institutions. PA is an NIHR senior investigator and funded by NIHR Oxford Biomedical Research Centre and CLAHRC. All funding is gratefully acknowledged.

ACKNOWLEDGEMENTS

We thank Carolyn Pallister and her colleagues at Slimming World for their cooperation and provision of the intervention free of charge to trial participants. We thank the Stop Smoking Services for their participation.

DATA STATEMENT

Please contact the corresponding author if you would like to access the trial data.

Figure 1: Flow of Participants through trial

For peer review only

Supplementary Figure 1. Reasons for declining to take part in SWISSS (more than 1 choice possible)

REFERENCES

1. Klesges RC, Winders SE, Meyers AW, et al: **How much weight gain occurs following smoking cessation? A comparison of weight gain using both continuous and point prevalence abstinence.** *J Consult Clin Psychol* 1997, **65**(2):286–291.

2. Klesges RC, Winders SE, Meyers AW, et al: **Smoking, body weight and their effects on smoking behavior: a comprehensive review of the literature.** *Psychol Bull* 1989, **106**:204–230.

3. Aubin H-J, Farley A, Lycett D, Lahmek P, Aveyard P. Weight gain in smokers after quitting cigarettes: meta-analysis BMJ 2012; 345 :e4439

4. Wannamethee SG, Shaper AG, Perry IJ: **Smoking as a modifiable risk factor for type 2 diabetes in middle-aged men.** *Diabetes Care* 2001, **24**(9):1590–1595.

5. Davey-Smith G, Bracha Y, Svendsen KH, Neaton JD, Haffner SM, Kuller LH; Multiple Risk Factor Intervention Trial Research Group: **Incidence of type 2 diabetes in the randomized multiple risk factor intervention trial.** *Ann Intern Med* 2005, **142**(5):313–322.

6. Yeh HC, Duncan BB, Schmidt MI, Wang NY, Brancati FL: **Smoking, smoking cessation, and risk for type 2 diabetes mellitus.** *Ann Intern Med* 2010, **152**(1):10–17.

7. Gerace TA, Hollis JF, Ockene JK, Svendsen KH: **Smoking cessation and change in diastolic blood pressure, body weight, and plasma lipids. MRFIT Research Group.** *Prev Med* 1991, **20**(5):602–620.

For peer review only

8. Lycett, D., Nichols, L., Ryan, R., et al. **The association between smoking cessation and glycaemic control in patients with type 2 diabetes: a THIN database cohort study**. *Lancet Diabetes Endocrinol* 2015. *3*(6), pp.423-430.

9. Clair, C., Rigotti, N.A., Porneala, B., et al. **Association of smoking cessation and weight change with cardiovascular disease among adults with and without diabetes.** *JAMA* 2013, 309(10), pp.1014-1021.

10. Luo J, Rossouw J, Margolis KL. **Smoking Cessation, Weight Change, and Coronary Heart Disease Among Postmenopausal Women With and Without Diabetes.** *JAMA*. 2013;310(1):94–96. doi:10.1001/jama.2013.6871

11. Brouwer RJN, Pomerleau CS: **"Prequit attrition" among weight-concerned women smokers.** *Eat Behav* 2000, **1**(2):145–151.

12. Cheskin LJ, Hess JM, Henningfield J, Gorelick DA: **Calorie restriction increases cigarette use in adult smokers.** *Psychopharmacology (Berl)* 2005, **179**(2):430–436.

13. Leeman RF, O'Malley SS, White MA, McKee SA: **Nicotine and food deprivation decrease the ability to resist smoking.** *Psychopharmacology (Berl)* 2010, **212**(1):25–32.

14. Hall SM, Tunstall CD, Vila KL, Duffy J: **Weight gain prevention and smoking cessation: cautionary findings.** *Am J Public Health* 1992, **82**(6):799–803.

15. Farley AC, Hajek P, Lycett D, Aveyard P: **Interventions for preventing weight gain after smoking cessation.** *Cochrane Database Syst Rev* 2012, **Jan 18**;1:CD006219.

For peer review only

16. Jolly K, Lewis A, Beach J, et al.: **Comparison of range of commercial or primary care led weight reduction programmes with minimal intervention control for weight loss in obesity: Lighten Up randomised controlled trial.** *BMJ* 2011, **343**:d7243.

17. Lycett, D., Aveyard, P., Farmer, A., Lewis, A. and Munafò, M. **Slimming World in Stop Smoking Services (SWISSS): study protocol for a randomized controlled trial**. *Trials*, 2013. *14*(1), p.182.

18. Kozlowski LT, Porter CQ, Orleans CT, Pope MA, Heatherton T. **Predicting smoking cessation with self-reported measures of nicotine dependence: FTQ, FTND, and HSI.** Drug and alcohol dependence. 1994 Feb 1;34(3):211-6.

19. Herdman M, Gudex C, Lloyd A, Janssen MF, Kind P, Parkin D, Bonsel G, Badia X. **Development and preliminary testing of the new five-level version of EQ-5D (EQ-5D-5L).** Quality of life research. 2011 Dec 1;20(10):1727-36.

20. Lycett D, Munafò M, Johnstone E, Murphy M, Aveyard P: **Associations between weight change over 8 years and baseline body mass index in a cohort of continuing and quitting smokers.** Addiction. 2011, 106: 188-196. 10.1111/j.1360-0443.2010.03136.x.

21. West R, Hajek P, Stead L, Stapleton J: **Outcome criteria in smoking cessation trials: proposal for a common standard.** Addiction 2005, 100(3):299–303.

22. West R, Hajek P: **Evaluation of the mood and physical symptoms scale (MPSS) to assess cigarette withdrawal.** Psychopharmacology 2004, 177:195–199.

23. Puchalski C, Romer AL: **Taking a spiritual history allows clinicians to understand patients more fully.** J Palliat Med 2000, 3:129–137.

For peer review only

24. Koenig HG, Cohen HJ, Blazer DG, et al.: **Religious coping and depression among elderly, hospitalized medically ill men.** Am J Psychiatry 1992, 149(12):1693–1700.

25. Lancaster, K.J., Carter‐Edwards, L., Grilo, S., Shen, C. and Schoenthaler, A.M.. **Obesity interventions in African American faith‐based organizations: a systematic review.** Obesity Reviews 2014, 15(S4), pp.159-176.

26. Gonzales D, Redtomahawk D, Pizacani B, et al.: **Support for spirituality in smoking cessation: results form a pilot survey.** Nicotine Tob Res 2007, 9(2):299–303.

27. Lindson-Hawley, N., Banting, M., West, R., Michie, S., Shinkins, B. and Aveyard, P., 2016. **Gradual versus abrupt smoking cessation: a randomized, controlled noninferiority trial.** Annals of internal medicine, 164(9), pp.585-592.

28. Devlin N, Shah K, Feng Y, Mulhern B, van Hout B. **Valuing health-related quality of Life: An EQ-5D-5L Value Set for England.** Health Economics. 2017;1-16

29. Bengtsson M. **How to plan and perform a qualitative study using content analysis,** NursingPlus Open 2016, Volume 2, Pages 8-14, ISSN 2352-9008, https://doi.org/10.1016/j.npls.2016.01.001.

30. Lader D: **A report on research using the National Statistics Opinions Survey produced on behalf of the NHS Information Centre for health and social care**. Office for National Statistics 2009.

For peer review only

31. Bush T, Levine MD, Beebe LA, et al. **Addressing weight gain in smoking cessation treatment: a randomized controlled trial.** Am J Health Promot. 2012 Nov-Dec;27(2):94-102. doi: 10.4278/ajhp.110603-QUAN-238.

32. Anderson JW, Konz EC, Frederich RC, Wood CL. **Long-term weight-loss maintenance: a meta-analysis of US studies**. The American journal of clinical nutrition. 2001 Nov 1;74(5):579-84.

33. Preloading Investigators. **Effects on abstinence of nicotine patch treatment before quitting smoking: parallel, two arm, pragmatic randomised trial**. *BMJ*, 2018. *361*, p.k2164.

34. Levine MD, Bush T, Magnusson B, Cheng Y, Chen X; **Smoking-Related Weight Concerns and Obesity: Differences Among Normal Weight, Overweight, and Obese Smokers Using a Telephone Tobacco Quitline**, Nicotine Tob Res, 2013. Volume 15, Issue 6, 1 June 2013, Pages 1136–1140, https://doi.org/10.1093/ntr/nts226

For peer review only

Characteristics	Decliners (n=128)	Participants (n=76)
**Age**	45.3 (14.1)	46.7 (13.5)
**Ethnicity (%)**		
**White British**	93% (n=119)	88% (n=67)
**White Irish**	1% (n=1)	
**White other**	2% (n=3)	
**Indian**	2% (n=2)	1% (n=1)
**White & Asian**		3% (n=2)
**Black – other**	1% (n=1)	8% (n=6)
**Unknown**	1% (n=2)	
**Gender**		(n=71)
**Female**	55% (n=70)	65% (n=46)
**Male**	45% (n=58)	35% (n=25)
**BMI category**	(self-report) (n=123)	(measured) (n=73)
**Underweight**	7% (n=8)	0% (n=0)
**Healthy Weight**	58% (n=71)	12% (n=9)
**Overweight**	35% (n=43)	34% (n=25)
**Obese**	1% (n=1)	53% (n=39)

Table 1. Characteristics of those who declined to take part and those who participated in the SWISSS trial

For peer review only

Characteristic (mean (SD)/% frequency)	Control (n=39)	Treatment (n=37)
Age (years)	50 (15)*	44 (12)*
BMI (kg/m^2)	31.6 (6.3)*	29.3 (4.3)*
FTND (scores range: 1-10)	5.7 (2.2)	5.8 (2.1)
MPSSM (scores range 7-35)	12.7 (5.7)	12.8 (4.1)
MPSSC (scores range 0-10)	6.9 (1.7)	7.3 (1.1)
MPSSP (scores range 3-15)	4.2 (1.8)	3.9 (1.1)
MPSST (scores range 10-60)	23.8 (7.3)	24.1 (4.9)
EQ-5D Index	0.759 (0.268)	0.848 (0.105)
Value	61 (23)	67 (15)
VAS (100 point scale)		
Using religion to cope score (scores range 1-10)	(n=23)	(n=21)
	2.7 (2.6)	3.7 (2.9)
Gender	(n=38)	(n=33)
Male	29%	44%
Female	71%	56%
Ethnicity White	92%	84%
British	5%	
White & Asian		3%
Indian	3%	13%
Unknown		
Highest Qualification	(n=38)	(n=32)
No formal qualification	18%	25%
GCSEs/O levels	21%	28%
A levels	42%	31%
Degree	13%	9%
Higher Degree	6%	7%
Employment	(n=38)	(n=32)
Employed	55%	63%
Long term sick	11%	0%
Looking after others	3%	6%
Retired	21%	13%
Unemployed	8%	13%
Other	2%	5%
Religious	(n=37)	(n=30)
No	95%	87%
Yes	5%	13%
Supportiveness of religious community	(n=20)	(n=14)
A lot	5%	35%
A little	15%	25%
Not at all	80%	30%
Impact of religious belief	(n=11)	(n=10)
Comfort	91%	100%
Stress	9%	0%
Pharmacotherapy used	(n=16)	(n=15)
Nicotine Replacement Therapy	38%	40%
(NRT)	62%	60%
Varenicline		
Family history of diabetes	(n=36)	(n=31)
Yes	25%	26%
No	75%	74%
Impaired glucose tolerance	16%	19%
Yes	84%	81%
No		
Absolute Diabetes Prevalence	(n=32)	(n=32)
Yes	14%	13%
No	86%	87%

GCSE: General Certificate of Secondary Education, O level: Ordinary level exams, A level: Advanced level exams.
*p<0.1 between control and treatment groups

Table2. Baseline characteristics by randomisation

Change in weight (kg) from baseline in quitters at:	Control	Treatment	Difference in weight change (treatment – control) mean [95% CI] adjusted for baseline weight as covariate
**4 weeks post quit**	(n=13)<br>0.7 (1.5)	(n=14)<br>-0.7 (1.8)	-1.2 [-2.5, 0.1]
**12 weeks post quit**	(n=8)<br>2.0 (1.9)	(n=12)<br>-1.3 (3.3)	-2.3 [-4.4, -0.1]
**26 weeks post quit**	(n=5)<br>2.3 (2.2)	(n=8)<br>-0.9 (2.5)	-3.1 [-6.9, 0.8]*

*Also adjusted for age (p<0.1 between treatment and control in 26 week quitters)

Table 3. Change in weight in quitters at 4, 12 & 26 weeks post quit

For peer review only

Change in Outcome from baseline (ITT-BOCF i.e. assumed no change)	At 4 weeks post quit			At 12 weeks post quit			At 26 weeks post quit		
	**Control**	**Treatment**	**Difference in change (treatment – control) OR / mean [95% CI]***	**Control**	**Treatment**	**Difference in change (treatment – control) OR / mean [95% CI]***	**Control**	**Treatment**	**Difference in change (treatment – control) % / mean [95% CI]***
**Abstinence rate**	13/39 (34%)	14/37 (38%)	1.2 [0.5, 3.1][1]<br>1.3 [0.4, 3.7][2]	8/39 (21%)	12/37(32%)	1.9 [0.7, 5.3][1]<br>2.9 [0.8, 10.1][2]	5/39 (13%)	8/37 (22%)	1.9 [0.6, 6.4][1]<br>3.2 [0.7, 14.4][2]
**MPSS – C (range 0-10)**	-0.8 (1.5)	-1.5 (2.1)	-0.7 [-1.6, 0.2][1]<br>-0.8 [-1.8, 0.1][2]	-0.7 (1.8)	-2.2 (2.7)	-1.5 [-2.6, -0.4][1]<br>-1.6 [-2.7, -0.5][2]	-0.7 (1.5)	-1.9 (2.5)	-1.3 [-2.2, -0.3][1]<br>-1.5 [-2.5, -0.4][2]
**MPSS – P (range 3-15)**	0.2 (1.3)	0.1 (1.0)	-0.1 [-0.7, 0.4][1]<br><0.1 [-0.6, 0.7][2]	<0.1 (1.6)	-0.1 (1.2)	-0.1 [-0.8, 0.5][1]<br><0.1[-0.7, 0.7][2]-	<0.1 (1.4)	-0.1 (1.1)	0.2 [-1.0, 1.4][1]<br>0.4 [-0.9, 1.7][2]
**MPSS- M (range 7-35)**	1.1 (3.8)	0.9 (4.9)	-0.2 [-2.4, 1.9][1]<br>0.1 [-2.2, 2.4][2]	-0.5 (3.7)	-1.4 (4.8)	1.0 [-3.0, 1.1][1]<br>-0.5 [-2.7, 1.7][2]	-0.6 (3.6)	-1.7 (5.0)	-1.3 [-3.6, 0.9][1]<br>-1.3 [-3.7, 1.2][2]
**Hunger (range 1-5)**	0.2 (0.8)	0.1 (0.9)	-0.1 [-0.5, 0.3][1]<br><-0.1[-0.5, 0.4][2]	0.1 (0.6)	0.1 (0.9)	<-0.1[-0.4, 0.3][1]<br><-0.1[-0.4, 0.3][2]	0.1 (0.5)	0.1 (0.9)	<-0.1[-0.4, 0.3][1]<br><-0.1[-0.4, 0.4][2]
**MPSS- T (range 10-60)**	0.5 (4.4)	-0.6 (6.0)	-1.1 [-3.6, 1.4][1]<br>-0.8 [-3.5, 1.9][2]	-1.3 (5.0)	-3.7 (7.4)	-2.6 [-5.6, 0.4][1]<br>-2.1 [-5.2, 1.1][2]	-7.1 (13.4)	-7.8 (14.5)	-0.8 [-7.5, 5.8][1]<br>-2.5 [-9.4, 4.3][2]
**EQ-5D Index Value**	0.008(0.065)	0.009(0.112)	0.006 [-0.053, 0.041][1]<br>-0.014 [-0.065, 0.037][2]	0.0165(0.086)	0.023(0.099)	0.006 [-0.039, 0.052][1]<br>0.008 [-0.040, 0.057][2]-	0.001 (0.069)	0.023(0.078)	0.003 [-0.062, 0.071][1]<br>0.006 [-0.059, 0.071][2]
**VAS (100 point scale)**	5.5 (20.9)	4.4 (16.2)	-2.1 [-9.4, 5.3][1]<br>-3.2 [-11.2, 4.7][2]	4.8 (12.7)	5.6 (12.7)	0.5 [7.5, 6.6][1]<br>-0.9 [-8.5, 6.7][2]	5.7 (15.2)	4.8 (11.8)	-1.1 [-7.7, 5.5][1]<br>-2.0 [--9.0, 5.0][2]
**Using religion to cope score (range 1-10)**	-0.3 (1.2)	-0.8 (1.9)	-0.6 [-1.7, 0.6][1]<br>-1.0 [-2.4, 0.4][2]	0.2 (0.5)	-0.3 (0.9)	-1.3[-2.3, -0.4][1]<br>-1.5 [-2.8, -0.3][2]	0.5 (1.2)	<0.1 (1.8)	-0.2 [-0.6, 0.2][1]<br>-0.2 [-0.7, 0.2][2]

[1] Unadjusted regression analysis
[2]Regression analysis adjusted for baseline BMI and age

Table 4. Change in abstinence and exploratory outcomes within and between the control and intervention group

For peer review only - http://bmjopen.bmj.com/site/about/guidelines.xhtml

Figure 1. Flow of participants through the trial

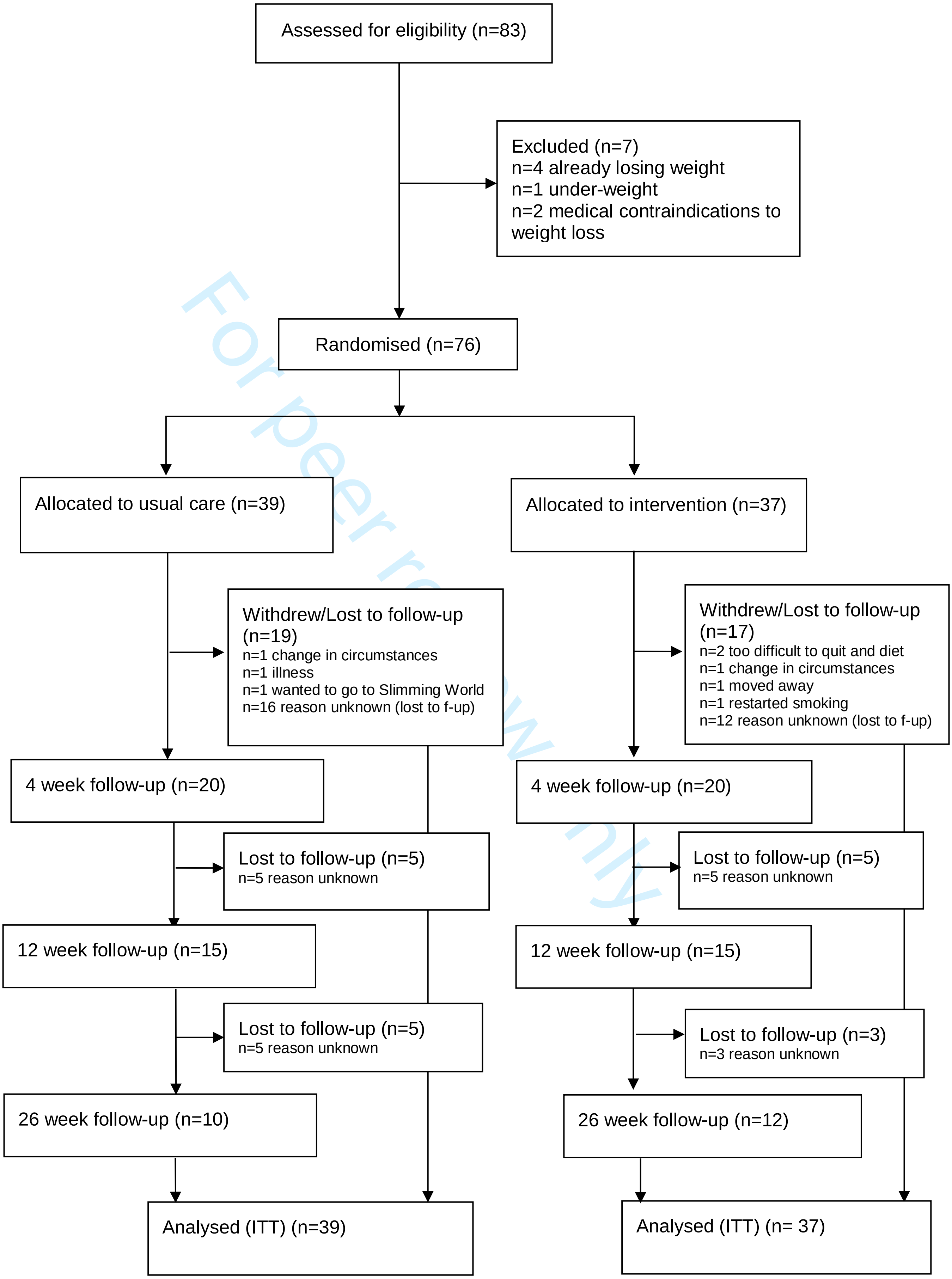

For peer review only

Code (number of times featured in data)	Sub theme	Theme
Good trial (2)	Positive aspects	Aspects related to research processes
Easy to implement (4)		
Additional [paper] work (3)	Time and additional effort on part of practitioner and patient	
Patient time (2)		
Practitioner time (7)		
Discussion of research viewed negatively (1)	Engaging in research was viewed positively and negatively	
Discussion of research viewed positively (1)		
Patient disappointment with randomisation (3)	Disappointment for participants and practitioners (and misunderstanding for practitioner) regarding randomisation	
Practitioner disappointment with randomisation (2)		
Slimming World vouchers seen as an positive incentive (2)		
Discussion of weight viewed negatively (1)	Discussing weight was seen as a positive and negative thing	An opportunity to address the weight issues
Discussion of weight viewed positively (5)		
Lack of patient interest in weight (4)	Patient interest and attendance	Poor participant interest and attendance
Patient drop out, transient population (3)		
Few people wanting to quit (2)		

Supplementary Table 1. Content analysis of open comments from practitioners at the end of the trial

For peer review only

Supplementary Figure 1. Reasons for declining to take part in SWISSS (more than 1 choice possible)

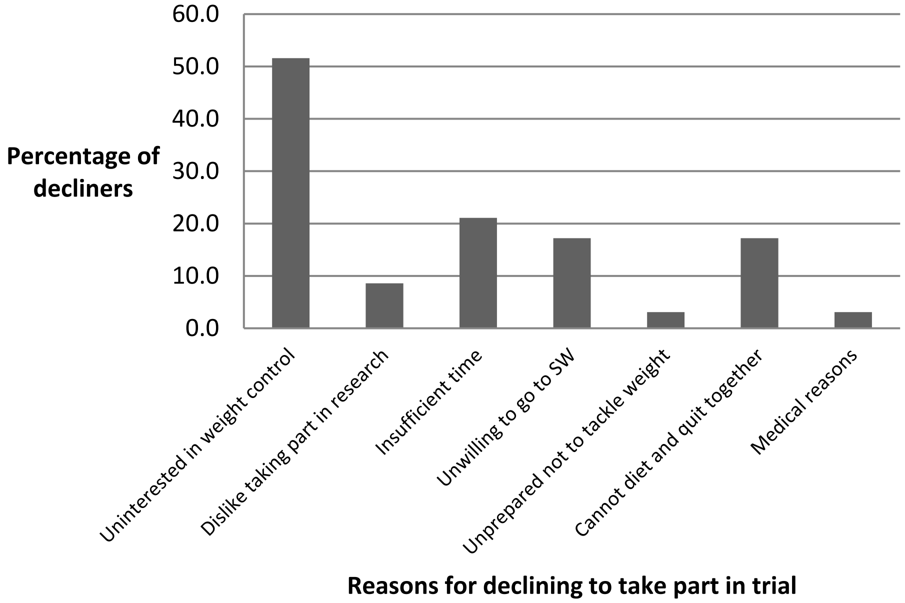

For peer review only

CONSORT 2010 checklist of information to include when reporting a randomised trial*

Section/Topic	Item No	Checklist item	Reported on page No
**Title and abstract**			
	1a	Identification as a randomised trial in the title	1
	1b	Structured summary of trial design, methods, results, and conclusions (for specific guidance see CONSORT for abstracts)	2-3
**Introduction**			
Background and objectives	2a	Scientific background and explanation of rationale	4
	2b	Specific objectives or hypotheses	5
**Methods**			
Trial design	3a	Description of trial design (such as parallel, factorial) including allocation ratio	5
	3b	Important changes to methods after trial commencement (such as eligibility criteria), with reasons	N/A
Participants	4a	Eligibility criteria for participants	6
	4b	Settings and locations where the data were collected	5
Interventions	5	The interventions for each group with sufficient details to allow replication, including how and when they were actually administered	6-7
Outcomes	6a	Completely defined pre-specified primary and secondary outcome measures, including how and when they were assessed	7
	6b	Any changes to trial outcomes after the trial commenced, with reasons	N/A
Sample size	7a	How sample size was determined	9
	7b	When applicable, explanation of any interim analyses and stopping guidelines	9
Randomisation:			
Sequence generation	8a	Method used to generate the random allocation sequence	7
	8b	Type of randomisation; details of any restriction (such as blocking and block size)	7
Allocation concealment mechanism	9	Mechanism used to implement the random allocation sequence (such as sequentially numbered containers), describing any steps taken to conceal the sequence until interventions were assigned	7
Implementation	10	Who generated the random allocation sequence, who enrolled participants, and who assigned participants to interventions	7
Blinding	11a	If done, who was blinded after assignment to interventions (for example, participants, care providers, those	N/A

For peer review only

For peer review only - http://bmjopen.bmj.com/site/about/guidelines.xhtml

		assessing outcomes) and how	
	11b	If relevant, description of the similarity of interventions	N/A
Statistical methods	12a	Statistical methods used to compare groups for primary and secondary outcomes	9
	12b	Methods for additional analyses, such as subgroup analyses and adjusted analyses	9
**Results**			
Participant flow (a diagram is strongly recommended)	13a	For each group, the numbers of participants who were randomly assigned, received intended treatment, and were analysed for the primary outcome	Figure 1
	13b	For each group, losses and exclusions after randomisation, together with reasons	Figure 1
Recruitment	14a	Dates defining the periods of recruitment and follow-up	8
	14b	Why the trial ended or was stopped	9
Baseline data	15	A table showing baseline demographic and clinical characteristics for each group	Table 2
Numbers analysed	16	For each group, number of participants (denominator) included in each analysis and whether the analysis was by original assigned groups	Figure 1
Outcomes and estimation	17a	For each primary and secondary outcome, results for each group, and the estimated effect size and its precision (such as 95% confidence interval)	Table 3 and table 4
	17b	For binary outcomes, presentation of both absolute and relative effect sizes is recommended	Table 4
Ancillary analyses	18	Results of any other analyses performed, including subgroup analyses and adjusted analyses, distinguishing pre-specified from exploratory	9
Harms	19	All important harms or unintended effects in each group (for specific guidance see CONSORT for harms)	3
**Discussion**			
Limitations	20	Trial limitations, addressing sources of potential bias, imprecision, and, if relevant, multiplicity of analyses	15
Generalisability	21	Generalisability (external validity, applicability) of the trial findings	16
Interpretation	22	Interpretation consistent with results, balancing benefits and harms, and considering other relevant evidence	16
**Other information**			
Registration	23	Registration number and name of trial registry	3
Protocol	24	Where the full trial protocol can be accessed, if available	5
Funding	25	Sources of funding and other support (such as supply of drugs), role of funders	18

*We strongly recommend reading this statement in conjunction with the CONSORT 2010 Explanation and Elaboration for important clarifications on all the items. If relevant, we also recommend reading CONSORT extensions for cluster randomised trials, non-inferiority and equivalence trials, non-pharmacological treatments, herbal interventions, and pragmatic trials. Additional extensions are forthcoming: for those and for up to date references relevant to this checklist, see www.consort-statement.org.

For peer review only - http://bmjopen.bmj.com/site/about/guidelines.xhtml
For peer review only
